# Independent evolution of oleate hydratase clades in Bacillales reflects molecular convergence

**DOI:** 10.3389/fmolb.2024.1485485

**Published:** 2024-12-12

**Authors:** Robert J. Neff, Priscilla C. Lages, Shannon K. Donworth, James D. Brien, Christopher D. Radka

**Affiliations:** Department of Microbiology, Immunology, and Molecular Genetics, University of Kentucky, Lexington, KY, United States

**Keywords:** oleate hydratase (OhyA), molecular evolution, Bacillales (Caryophanales), phylogenetics, protein family, *Staphylococcus aureus* (*S. aureus*)

## Abstract

Oleate hydratase (OhyA), a flavoenzyme that catalyzes the hydration of unsaturated fatty acids, has been identified in various Bacillales organisms, including those in the *Listeria*, *Lysinibacillus*, *Paenibacillus*, and *Staphylococcus* genera. In this study, we combine structural biology with molecular and phylogenetic analyses to investigate the evolutionary dynamics of the OhyA protein family within the Bacillales order. Our evolutionary analysis reveals two distinct OhyA clades (clade I and clade II) within Bacillales that, while sharing catalytic function, exhibit significant genomic and structural differences. Our findings suggest that these OhyA clades originated from independent evolutionary processes through convergent evolution rather than gene duplication. We also show that the evolutionary divergence in OhyA is likely due to intrinsic sequence variations rather than being strictly linked to functional domain changes. Furthermore, within the *Staphylococcus* genus, we observed that the evolution of the *ohyA* gene aligns with the species tree, supporting a common ancestral origin. This study enhances our understanding of the impact of evolutionary history on the structure and function of OhyA across the Bacillales order.

## 1 Introduction

Fatty acid biosynthesis is a critical cellular process that requires significant energy expenditure and the consumption of essential cellular intermediates such as acetyl-coenzyme A and NAD(P)H (Rock and Jackowski, 2002). This process is vital for maintaining membrane integrity and producing signaling molecules, yet it presents a significant metabolic burden for many organisms. To mitigate this burden, some bacteria have evolved specialized acquisition systems that enable them to utilize extracellular fatty acids directly for membrane biosynthesis. These systems, such as fatty acid kinase and acyl-acyl carrier protein synthetase, activate extracellular free fatty acids, allowing them to be incorporated into the bacterial lipid metabolic program (Radka, 2023). However, an alternative strategy employed by certain bacteria involves the utilization of oleate hydratase (OhyA, ENZYME entry EC 4.2.1.53), a flavoenzyme that catalyzes the hydration of unsaturated fatty acids, transforming them into hydroxylated fatty acids (Radka et al., 2021b).

In bacteria like *Staphylococcus aureus*, the product of OhyA activity, 10-hydroxystearic acid, is not utilized for membrane lipid biosynthesis but is instead secreted into the extracellular environment (Subramanian et al., 2019). This secretion serves a dual purpose: it reduces the toxicity of antimicrobial unsaturated fatty acids present in the environment and modulates the host immune response, thereby promoting bacterial survival and virulence (Radka et al., 2021a; Radka et al., 2024a). The role of OhyA in this context underscores its importance in bacterial adaptation and survival in hostile environments, particularly during host-pathogen interactions.

**FIGURE 1:**
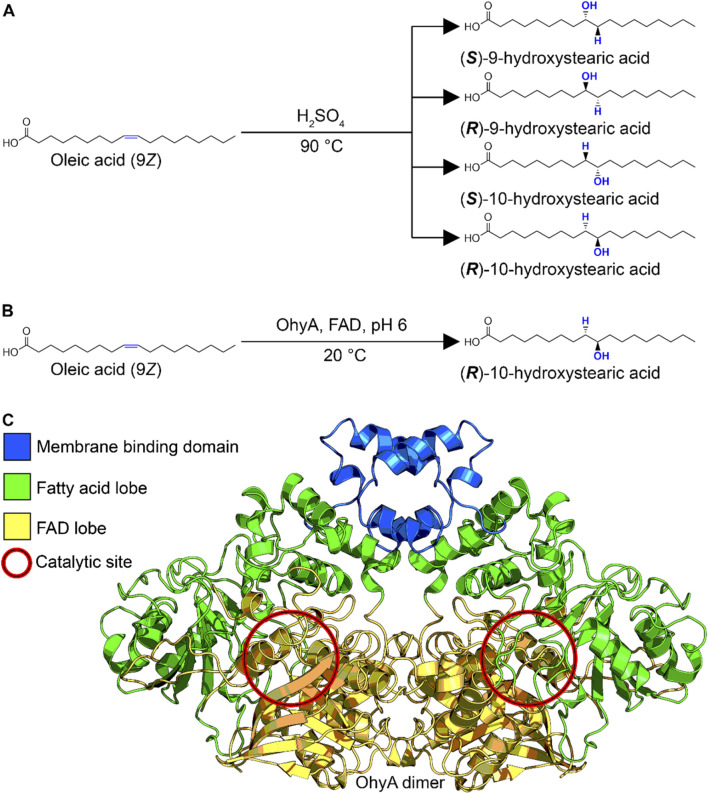
Biochemical production of hydroxylated fatty acid. **(A)** Reaction scheme for organic chemistry synthesis of hydroxylated fatty acid. The uncontrolled acid-catalyzed water addition reaction produces a racemic mixture. **(B)** Reaction scheme for oleate hydratase enzymological synthesis of hydroxylated fatty acid. The biological substrate-assisted acid-catalyzed water addition reaction produces a single stereoisomer. **(C)** Three-dimensional structure of *Staphylococcus aureus* oleate hydratase dimer (PDB ID: 7KAV), colored by functional domain.

Beyond their biological functions, hydroxylated fatty acids have considerable commercial value. They are used as key intermediates or end products in various industries, including cosmetics, food preservatives, chemical synthesis, and pharmaceuticals (Anadón et al., 2010; Cao and Zhang, 2013; Hagedoorn et al., 2021). The traditional chemical synthesis of hydroxylated fatty acids often involves high temperatures and harsh conditions, leading to racemic mixtures with limited application (Solomons and Fryhle, 2011) (Figure 1A). In contrast, OhyA catalyzes the stereospecific hydration of unsaturated fatty acids at ambient temperatures, producing a single stereoisomer with high specificity (Engleder and Pichler, 2018; Radka et al., 2021b) (Figure 1B). This enzymatic process offers a more sustainable and efficient alternative for industrial applications.

OhyA belongs to the myosin-cross-reactive antigen family of proteins (Pfam entry PF06100), which is part of the larger NADP Rossman protein clan (Pfam clan entry CL0063). The enzyme is characterized by a flavoenzyme structure with a Rossmann domain where the NAD + cofactor is replaced by FAD (Rosberg-Cody et al., 2011; Radka et al., 2021b). The crystal structure of *S. aureus* OhyA (PDB ID: 7KAV) provides significant insights into its functional domains. The enzyme functions as a homodimer, with each protomer containing three distinct domains: a lobe for dimerization and FAD binding, a lobe for unsaturated fatty acid binding, and a membrane binding domain for accessing membrane-bound fatty acids (Oldham et al., 2024; Radka et al., 2024b) (Figure 1C). The catalytic activity of OhyA occurs at the interface between the lobes, highlighting the intricate coordination required for its function.

The InterPro database classification of protein families (https://www.ebi.ac.uk/interpro/) reveals that OhyA is predominantly encoded by bacterial species (87.9%), with smaller representations in eukaryotes (9.8%) and archaea (2.2%). This distribution suggests that OhyA plays a crucial role in various metabolic pathways across different domains of life. OhyA activity is linked to over 50 metabolic pathways, indicating its versatility and functional significance beyond immune modulation. For instance, in lactic acid bacteria, OhyA catalyzes the first step in the production of conjugated linoleic acid, a bioactive lipid with health-promoting properties, in the intestinal tract (Yang et al., 2013). In the female genital tract, OhyA catalyzes the reversible hydration of oleic acid, biochemically sequestering the nutrient in a derivative form that is inaccessible to organisms lacking OhyA (Zhu et al., 2024).

A previous study classified 2,046 putative OhyA sequences into 11 homologous families (HFams) using a sequence similarity network (Schmid et al., 2016). This classification revealed significant sequence variability in key regions, such as the catalytic loop (Radka et al., 2021b) and the FAD binding motif (Hiseni et al., 2015), which are critical for enzyme function. However, the study lacked an evolutionary context and did not fully explore the biochemical significance of these variations. Understanding the evolutionary history of OhyA is essential for elucidating how gene evolution occurs within this protein family, as well as the potential range of biological functions this protein can complete.

**FIGURE 2:**
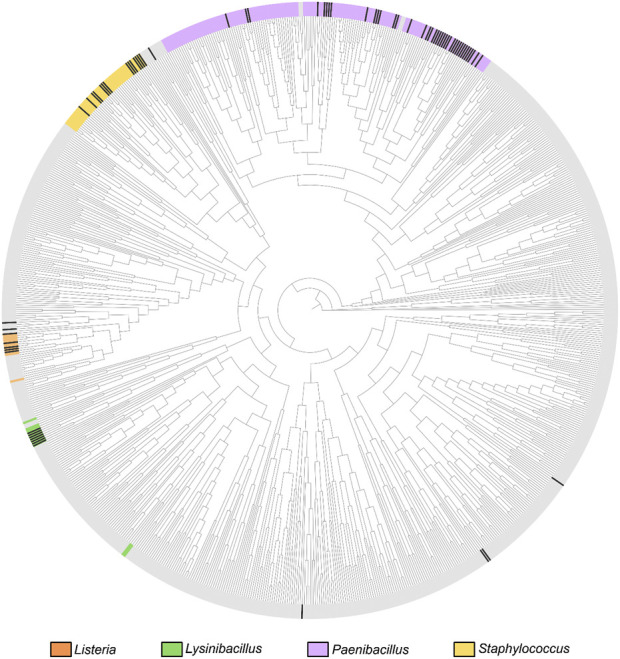
Bacillales phylogenetic relationships based on the TimeTree. Circular phylogenetic tree representation of the Bacillales TimeTree from TimeTree5 (https://timetree.org/home). The coloration on the crust of the tree indicates the distribution of species belonging to the four major OhyA genera, as labeled next to the tree. Gray crust indicates a species that does not belong to the four major genera. OhyA-producing species are marked with black ticks in the crust.

In this study, we focus on the evolutionary history of OhyA within the taxonomic order Bacillales, of which only 144/1,434 (10%) reference genomes encode *ohyA*. The genera *Staphylococcus*, *Bacillus*, *Lysinibacillus*, and *Paenibacillus* account for 82.6% of OhyA-encoding Bacillales reference genomes (Figure 2; Supplementary Tables S1, S2), making them a focal point for understanding the evolution of this protein family. The TimeTree of Life (https://timetree.org) synthesizes published molecular timetrees into a global timetree of life with estimated divergence times (Kumar et al., 2022). The TimeTree of Life estimates the adjusted molecular time of divergence of the four dominant OhyA-encoding genera to be one billion years ago at the transition between the Meso-Proterozoic and the Neo-Proterozoic eras, based on published molecular time trees (Moreno-Letelier et al., 2011; Marin et al., 2017). The phylogenetic distance between genera that encode OhyA suggests a convergent evolution scenario in which similar environmental selective pressures (e.g., the need to hydrate isolated double bonds in fatty acids) drove different bacterial lineages to evolve analogous enzymatic capabilities. However, OhyA is not encoded by all species of OhyA-encoding genera indicating there is not a strict metabolic requirement to retain OhyA in these organisms.

Not all species of a genus encode *ohyA*, and the loss of the *ohyA* gene in most of the Bacillales species can be attributed to several evolutionary pressures. One prominent scenario is the concept of genomic streamlining where bacterial adaptation to new environments with unique nutrient compositions leads to the loss of unnecessary metabolic pathways (Giovannoni et al., 2005; Simonsen, 2022). In cases where unsaturated fatty acids are not a prevalent metabolite, bacteria may lose the *ohyA* gene to conserve energy and resources. A similar scenario could be linked to a lifestyle that specifically relies heavily on fatty acids where bacteria that thrive in host environments may have retained the ability to produce hydroxy fatty acids because their survival strategy requires production of the metabolite. This gene loss reflects an adaptation to niche specialization, where the selective pressures favor the retention of genes that support the organism’s immediate needs (Gubry-Rangin et al., 2011; Baquero et al., 2021). The skewed pattern of minority gene retention and majority gene loss suggests that OhyA is primarily under negative selective pressures across different ecological contexts.

We classify OhyA sequences into two distinct phylogenetic groups, clade I and clade II, based on intrinsic sequence differences rather than differences in functional domains. This classification provides a framework for exploring the evolutionary pressures that shaped the diversification of OhyA. Within the *Staphylococcus* genus, a major representative of Bacillales, we found that 21 out of 69 species encode OhyA. Despite the enzyme’s role in *S. aureus* pathogenesis, OhyA is not conserved across all mammal-associated species of *Staphylococcus*, suggesting that its retention is influenced by factors other than mammalian association. The *ohyA* gene tree closely mirrors the *Staphylococcus* species tree, supporting a scenario of convergent evolution where independent events led to the rise of the two OhyA clades. Over evolutionary time, at least half of the OhyA genes were lost, with contemporary OhyA-encoding species primarily being non-mammal-associated. This pattern indicates that multiple selective pressures, beyond mammalian association, have played a role in maintaining OhyA in contemporary bacterial species.

## 2 Materials and methods

### 2.1 Phylogenetic analysis of Bacillales OhyA sequences

The Newick tree file from the Bacillales phylogenetic TimeTree, which was generated from ribosomal RNA sequences (Marin et al., 2017), was visualized in TreeViewer 2.2.0 (Bianchini and Sanchez-Baracaldo, 2024). A list of putative OhyA sequences were obtained from tBLASTn by querying the clade I *S. aureus* OhyA amino acid sequence (NCBI-ProteinID: BAB41321) or clade II *Listeria* monocytogenes OhyA amino acid sequence (NCBI-ProteinID: NP_464009) against RefSeq Representative genomes in Bacillales/*Caryophanales* (taxid:1385). Default algorithm parameters (e.g., expect threshold 0.05, BLOSUM62 matrix, low complexity filer yes) were used for the tBLASTn search (Supplementary Tables S1, S2). Ten *Paenibacillus* species contained multiple hits in the same genome and both sequences were included in the analysis. Partial sequences, identified by a maximum alignment score <300, or sequences containing premature stop codons, identified by manual inspection, were removed to yield a final OhyA dataset of 145 sequences.

Species encoding putative *ohyA* genes were mapped onto the Bacillales TimeTree. Multiple sequence alignment was performed using MUSCLE within Mega 11.0.13 (Tamura et al., 2021) (Supplementary Datasets S1 and S2). Amino acid alignments were generated and refined using the following parameters: gap open −2.9, extend 0, hydrophobicity multiplier 1.2, max iterations 16, cluster method UPGMA, and min diag length 24. The phylogenetic trees of Bacillales OhyA amino acid sequences were constructed using IQ-TREE 2.1.4-beta. The best substitution model for accurate phylogenetic estimates was determined by ModelFinder (Kalyaanamoorthy et al., 2017) in IQTree. The OhyA amino acid tree was constructed using LG + I + G4 as the model of substitution. The outgroup *Elizabethkingia meningoseptica* OhyA (Engleder et al., 2015) was used for rooting. The *Staphylococcus* 16S rRNA tree was constructed using TPM2+F + I + G4 as the model of substitution, and the *Staphylococcus ohyA* nucleotide tree was constructed using GTR + F + I + G4 as the model of substitution. The IQ-TREE phylogenetic trees were constructed with 5,000 ultrafast bootstrap alignments (Minh et al., 2013), 5,000 maximum iterations, 1,000 replicates of the single branch test, auto-detect threads, and a minimum correlation coefficient of 0.99. *Staphylococcus* phylogenetic trees were rooted at the midpoint using TreeViewer 2.2.0 (Bianchini and Sanchez-Baracaldo, 2024), and then re-rooted and re-ordered for analysis using the beta version of phylo.io (https://beta.phylo.io) (Robinson et al., 2016) in compare mode. Sequence logo and residue composition probability for each catalytic loop clade were generated using WebLogo 3.7.12 (https://weblogo.threeplusone.com/create.cgi), using units of probability. The amino acid sequence percent identity matrix was generated by Clustal Omega (https://www.ebi.ac.uk/jdispatcher/msa/clustalo).

### 2.2 Three-dimensional structure analysis of Bacillales OhyA sequences

Protomer models for all putative Bacillales OhyA sequences were generated using AlphaFold 2.3.0 (Jumper et al., 2021). The structural alignment, similarity statistics (e.g., Q-score, P-score, Z-score), and R.M.S.D. were calculated by submitting the AlphaFold-generated PDB files to the PDBeFOLD server (Krissinel and Henrick, 2004) (https://www.ebi.ac.uk/msd-srv/ssm/). Although it was not used for this study, we expect that using the testing phase of AlphaFold three would yield similar results and not affect the conclusions of the study.

### 2.3 Phylogenetic analysis of *Staphylococcus* OhyA sequences

The 16S rRNA nucleotide sequences of *Staphylococcus* species were obtained from the Kyoto Encyclopedia of Genes and Genomes (KEGG). 16S rRNA sequences for species that were not in KEGG were obtained from GenBank. Multiple sequence alignment was performed using MUSCLE within Mega 11.0.13. The multiple sequence alignment output was inspected to ensure the alignment was reasonable. The phylogenetic species tree was constructed from the 16S rRNA nucleotide sequences using IQ-TREE 2.1.4-beta, using GTR + F + G4 as the model of substitution. The *Staphylococcus ohyA* gene nucleotide sequences that were returned from the tBLASTn search described above were aligned and the phylogenetic *ohyA* gene tree was constructed using IQ-TREE 2.1.4-beta, using GTR + F + G4 as the model of substitution. The best substitution model for accurate phylogenetic estimates was determined by ModelFinder (Kalyaanamoorthy et al., 2017) in IQTree. Phylogenetic trees were constructed with 5,000 ultrafast bootstrap alignments (Minh et al., 2013), 5,000 maximum iterations, and a minimum correlation coefficient of 0.99. The *ohyA* gene context was determined by manual genome inspection and functional categorization. Gene length and orientation were used to scale graphics for accurate visualization.

### 2.4 Synteny around OhyA analysis

The *ohyA* gene position was manually identified in each *Staphylococcus* species, and ∼5 k base pairs upstream and downstream were analyzed for open reading frames and predicted functions (Supplementary Table S3). Functional annotations were used to group open reading frames into seven broad functional categories: protease, transcriptional regulation, transport, nucleic acid metabolism, stress response, carbon and nitrogen metabolism, and hypothetical protein. Open reading frames in the transcriptional regulation group were searched against *S. aureus subsp. aureus* NCTC 8325 (taxid: 158879) using BLASTn.

### 2.5 Bacteriology and quantitative real time PCR (qRT-PCR)

The *S. aureus* USA300 JE2 strains used in this study are from the Nebraska Transposon Mutant Library (BEI Resources, Cat. No. NR-48501): wild type/parent strain (NR46S43); Δ*srrA* knockout strain (NE1309, NE-47852, SAUSA300_1442); Δ*srrB* knockout strain (NE588, NR-47131, SAUSA300_1441). *Staphylococcus aureus* strains were grown on mannitol salt agar overnight at 37°C, and then single colonies were grown in tryptic soy broth (TSB) shaking overnight at 200 rpm at 37°C. Cultures were diluted to OD_600_ = 0.3 and treated for 2 h with 5 mM hydrogen peroxide (CVS Pharmacy, Inc, Cat. No. NDC 51316-268-16) or 5 mM diethylamine/nitric oxide complex sodium salt hydrate (MilliporeSigma, Cat. No. D184). Erythromycin (5 μg/mL) was used to maintain pressure on the knockout strains throughout the experiment. Cells were harvested (OD_600_ ˜ 0.6) using a phenol:chloroform 1:1 solution, and RNA was extracted using the SV Total RNA Isolation System (Promega, Cat. No. Z3100) according to the manufacturer’s instructions.

The qRT-PCR was performed on a 7500 Fast Dx Real-Time PCR Detection System (Thermo Fisher Scientific) using TaqMan™ Fast Virus 1-Step Master Mix (Thermo Fisher Scientific, Cat. No. 4444436).

The primers sequences for *ohyA* are*:*

Probe: 5′-/56FAM/TGGTAACTTTGCAGAAACAGAGCGAGA/36TAMp/-3′

Forward: 5′-CATGGCAGTACGAACCGAATA-3′

Reverse: 5′-CTTTAGTCGTCCCGCATCAA-3′

The primers sequences for the *gyrA* calibrator are:

Probe: 5′/56FAM/CGGTATCACTGATTTACGTGATGAAAC/36-TAMp/3′

Forward: 5′-CCTTAGCGACATCAATAACGACACGC-3′

Reverse: 5′-AATTGCAGAGCTCGTTCGTGACAAG-3′

Cycling conditions were reverse transcription for 5 min at 50°C and pre-denaturation for 20 s at 95°C, followed by 40 cycles of denaturation for 15 s at 95°C and annealing for 60 s at 60°C. Fluorescent signals were detected at the end of each cycle. The relative fold gene expression was calculated using the 2^−ΔΔCT^ method (Livak and Schmittgen, 2001).

### 2.6 Quantification and statistical analysis

Quantification and data analysis (i.e., calculation of mean, standard deviation, significance testing) were performed using PRISM 9.5.1 (GraphPad Software, https://www.graphpad.com/scientific-software/prism/).

## 3 Results

### 3.1 Evolutionary analysis of OhyA in the Bacillales order

**FIGURE 3:**
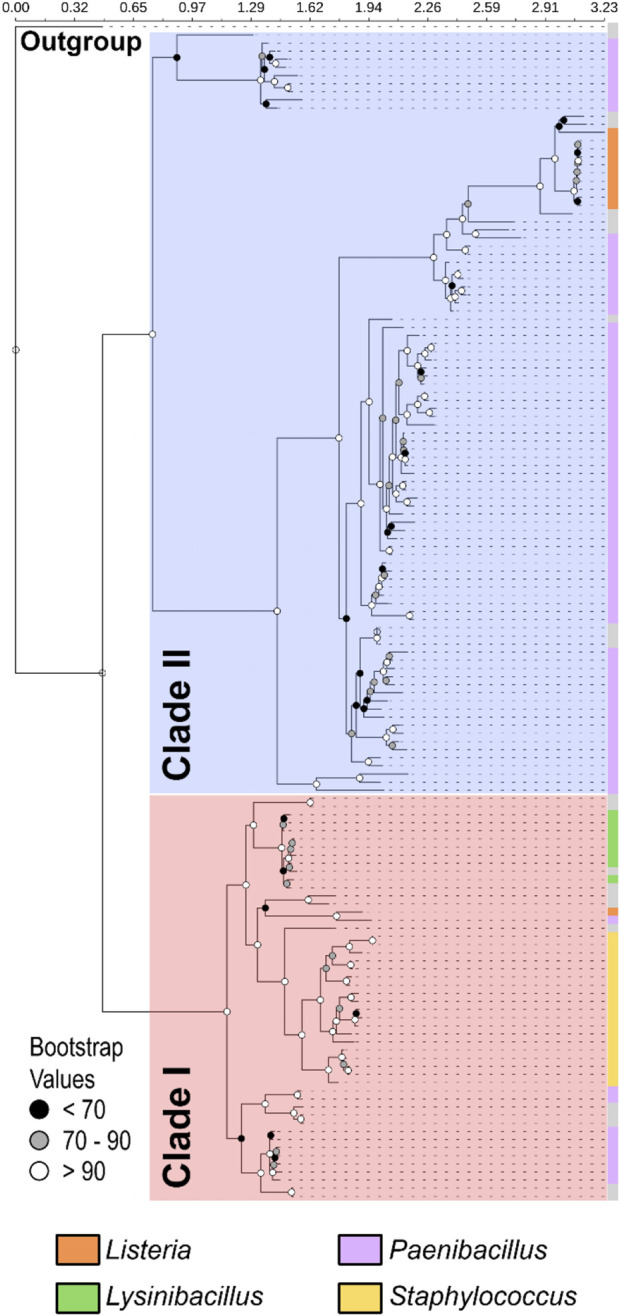
Phylogenetic analysis of OhyA identifies two clades. Phylogram constructed from 145 amino acid sequences with outgroup rooting reveals two distinct clades within Bacillales OhyA sequences: clade I (red shading) and clade II (blue shading). The total length of the multiple sequence alignment is 671 residues. Bootstrap values supporting the nodes of the consensus tree are represented by spheres. The outer coloring (crust) of the tree indicates the distribution of species from the four major *ohyA*-containing genera in Bacillales, with species of the same genera segregating together by clade. The coloration on the crust of the tree indicates the distribution of species belonging to the four major OhyA genera, as labeled next to the tree. Gray crust indicates a species that does not belong to the four major genera.

To investigate the evolutionary dynamics of OhyA, we performed a comprehensive evolutionary analysis of putative *ohyA* genes from Bacillales species. Using tBLASTn with *S. aureus* OhyA as the query sequence, we built an *ohyA* nucleotide sequence database, identifying 145 sequences with E-values ranging from 0.0 to 2.00E-91 and identity levels between 35.02% and 97.53% (Supplementary Tables S1, S2). The *ohyA* nucleotide phylogenetic tree, computed using a nucleotide model, revealed two distinct OhyA clades, which we named clade I and clade II; however, species from the same genera were not clustering together. Given the broad sequence diversity, including those with less than 40% identity to the query, we constructed a phylogenetic tree based on amino acid sequences (Figure 3). The amino acid tree, supported by similar bootstrap values, reproduced the two OhyA clades and sequences from species of the same genus clustered together. These data suggest that *ohyA* nucleotide variation is largely due to synonymous mutations that do not alter the encoded protein. Clade I OhyAs are present in *Staphylococcus*, *Lysinibacillus*, and 10 out of the 84 *Paenibacillus* species analyzed. Clade II OhyAs, on the other hand, are found in 10 out of 11 *Listeria* species and 74 out of 84 *Paenibacillus* species analyzed. Notably, the only *Listeria* species encoding a clade I OhyA is *Listeria grayi*, an avirulent species that has been proposed for reclassification under the genus *Murraya* (Stuart and Welshimer, 1974), through it is still categorized as *Listeria* for the time being (Rocourt et al., 1987). The phylogenetic distribution of OhyA reveals that it is found not only in pathogenic organisms but also in environmental microbes that inhabit diverse terrestrial ecosystems. This suggests that OhyA may offer niche-specific metabolic advantages beyond functioning as a defense mechanism against the immune system.

**FIGURE 4:**
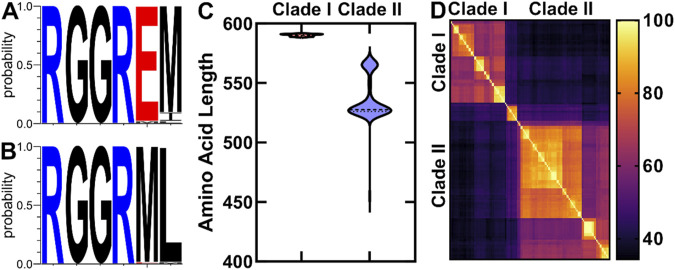
Comparative analysis of OhyA amino acid sequences. **(A, B)** Amino acid sequence logos of the conserved OhyA catalytic loop (residues R78–M83 in *Staphylococcus aureus* OhyA) from Clade I **(A)** and Clade II **(B)** OhyAs. **(C)** Violin plot of the amino acid length distribution for Clades I and II OhyAs. **(D)** Amino acid sequence percent identity matrix of Bacillales OhyAs.

We found that OhyA proteins from the two clades diverge in key features, including the amino acid sequence of the catalytic loop (Figures 4A, B), protein length (Figure 4C), and sequence conservation (Figure 4D). Clade I enzymes exhibit a narrow length range of 590.2 ± 0.8 amino acids, while clade II enzymes display greater variability, with an average length of 536.6 ± 18.4 amino acids. Within each clade, amino acid sequence similarity is 73.1% ± 9.4%, whereas similarity between the two clades is lower at 41.7% ± 7.5%. Notably, clade II enzymes lack a conserved glutamate residue, essential for catalysis in clade I OhyA (RGGREM) as described by Radka et al. (2021b).

**FIGURE 5:**
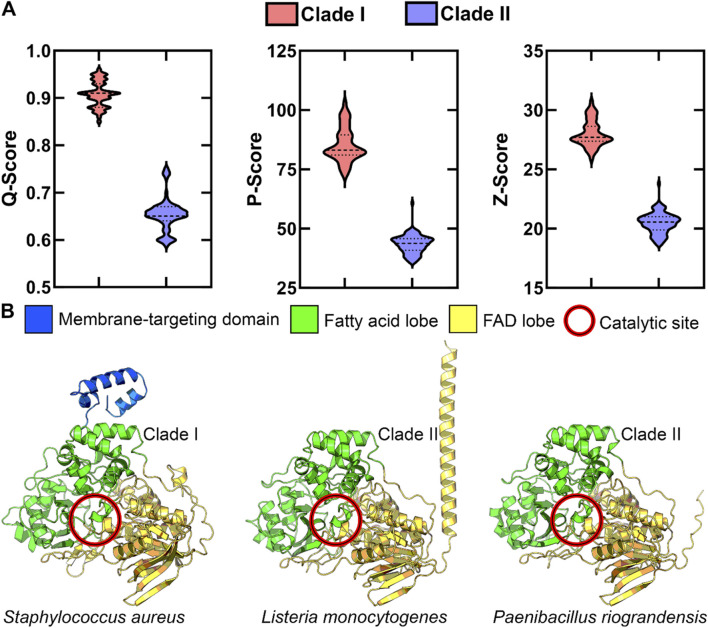
OhyA structure-based alignment supports two clades. **(A)** Statistical evaluation of alignment quality to clade I *Staphylococcus aureus* OhyA using PDBeFOLD. AlphaFold models for all Bacillales OhyA were employed for alignment. The distinct separation between clade I and clade II models suggests significant structural differences between the two clades. Dotted lines indicate the median and quartiles. **(B)** AlphaFold protomer models of clade I *Staphylococcus aureus,* clade II *Listeria monocytogenes*, and clade II *Paenibacillus riograndensis*, colored by functional domain.

To further explore structural diversity within the OhyA protein family, we used AlphaFold (Jumper et al., 2021) to predict the three-dimensional structures of Bacillales OhyA proteins. Pairwise structural comparisons, conducted using PDBeFOLD (Krissinel and Henrick, 2004), showed that the predicted structures cluster into two groups, corresponding to the clades identified in the phylogenetic analysis (Figure 5A). Despite low amino acid sequence similarity, the core catalytic domain structure is highly conserved across both clades. This is evidenced by a Root Mean Square Deviation (RMSD) of less than 2 Å in pairwise comparisons of Cα atoms in matched residues, with clade I enzymes having 569.6 ± 12.4 matched atoms and clade II enzymes having 486.6 ± 3.1 matched atoms.

Clade I enzymes typically feature a three-domain architecture consisting of a fatty acid lobe, FAD lobe, and a membrane binding domain. In contrast, clade II enzymes lack the characteristic membrane binding domain at the carboxy terminus, through some clade II enzymes feature a distinct alpha helix at the amino terminus (Figure 5B). This structural divergence suggests that clade II enzymes may have evolved alternative mechanisms for interacting with membrane-bound substrates, demonstrating the evolutionary flexibility in solving similar biochemical challenges. These structural and sequence variations indicate the presence of at least two distinct biochemical mechanisms for unsaturated fatty acid hydration within the OhyA family.

### 3.2 Molecular evolution of OhyA in *Staphylococcus*

**FIGURE 6:**
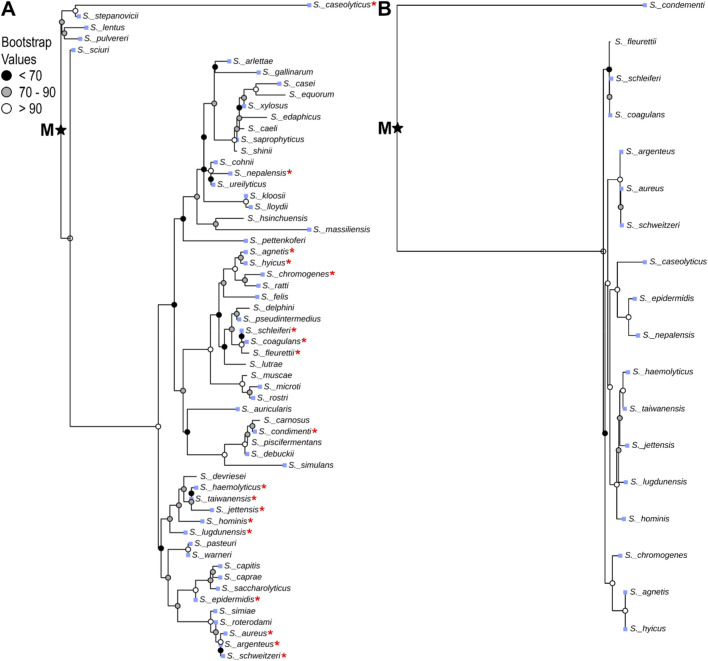
Species-level phylogenetic analysis of *Staphylococcus* OhyA. *Staphylococcus* species tree constructed from 16S rRNA nucleotide sequences **(A)**, and gene tree constructed from *ohyA* nucleotide sequences **(B)** with midpoint rooting (midpoint root, M). Red star indicates species that encode *ohyA* in the species tree. Blue squares indicate species that are associated with colonizing mammalian hosts.

Evolution and gene regulation in the *Staphylococcus* genus are well studied (Le and Otto, 2015; Coates-Brown et al., 2018; Raghuram et al., 2022), and *Staphylococcus* species exclusively encode clade I OhyA. Therefore, a deeper investigation into Staphylococcal OhyA could provide generalizable evolutionary and regulatory insights applicable to other, less-studied Bacillales genera. To investigate the molecular evolution of *ohyA* within the *Staphylococcus* genus, we employed a comparative genomics approach using phylogenetic and experimental bacteriology. We constructed a 16S rRNA phylogenetic tree for *Staphylococcus* species, revealing that 18/59 species encode genomic *ohyA* orthologs, with one species encoding an *ohyA* ortholog on a plasmid [*Staphylococcus condiment* plasmid pStO 2014-01 (Gabrielsen et al., 2017)] (Figure 6). BLAST E-values indicate all of the putative Staphylococcal *ohyA* genes are statistically homologous to the *S. aureus ohyA* gene (Supplementary Table S1), and the topology of the phylogenetic tree of *Staphylococcus* species that encode *ohyA* indicate a shared evolutionary origin. Further, there is good agreement between the Staphylococcal speciation nodes in 16S rRNA species tree and the *ohyA* gene tree (Figure 6; Supplementary Table S1).

Incongruence of phylogenetic trees or G + C content are traditional methods to identify candidate genes of horizontal gene transfer. The Horizontal Gene Transfer (HGT-DB) Database predicts the genomes of *S. aureus* strains contain 4.6%–5.8% horizontally transferred genes based on extraneous G + C content (Garcia-Vallve et al., 2003). The *S. aureus* strain N315 is a clinically relevant, methicillin-resistant reference strain that is commonly used in the laboratory setting Kuroda et al. (2001); (Gerlach et al., 2018). The representative genome from *S. aureus* strain N315 is 33.2% ± 0.3% G + C across 2,594 open reading frames and its *ohyA* is 35.8% G + C, which are congruent to each other. Although HGT-DB predicts *S. aureus* strain N315 encodes 105 horizontally transferred genes, *ohyA* is not one of them. The HGTree database identifies candidates for horizontally transferred genes by tree reconciliation between gene trees and 16S rRNA reference species trees (Jeong et al., 2016). HGTree predicts *S. aureus* strain N315 encodes 542 horizontal transfer events, *ohyA* is not one of them and is consistent with our observation of similar speciation between the *Staphylococcus* species tree and the *ohyA* gene tree. Thus, both methods agree that *ohyA* is not a Staphylococcal horizontally transferred gene.

The high amino acid sequence similarity among *ohyA* genes within *Staphylococcus* species, the congruence between *ohyA* G + C content and overall genomic G + C content, and the agreement between the *ohyA* gene tree and the *Staphylococcus* species tree all support an evolutionary scenario in which an ancestral *Staphylococcus* species encoded *ohyA*. During evolutionary processes, many contemporary *Staphylococcus* species lost the *ohyA* gene, leading to the current distribution of *ohyA*-encoding species (Figure 6). This pattern of gene loss is consistent with the concept of genetic streamlining, where genes that are not essential for survival in a given environment are selectively lost to reduce metabolic burden.

### 3.3 Genomic context and genetic regulation

**FIGURE 7:**
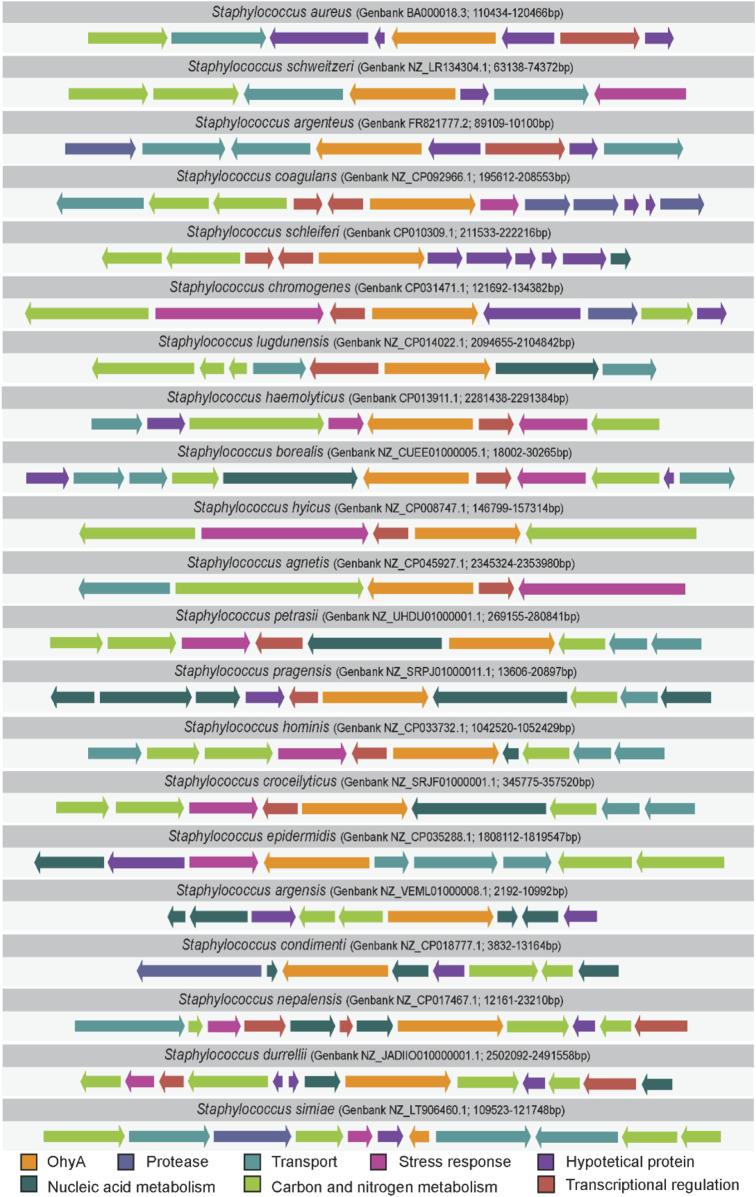
*Staphylococcus ohyA* synteny. Comparison of the genes that flank *ohyA* in *Staphylococcus* species. Arrows are scaled by nucleotide sequence length, indicate gene orientation, and are colored by functional category. The GenBank accession number and base pair range analyzed is provided for each *Staphylococcus* specie.

The synteny around *ohyA* provides further insights into its regulation and potential co-evolution with other genes. Alterations in the mechanisms of transcriptional regulation can lead to expression divergence, influencing the evolutionary path of a protein (Gu et al., 2002; Makova and Li, 2003). We analyzed the genetic loci flanking *ohyA* in *Staphylococcus* species to assess synteny and identify regulatory elements that may influence *ohyA* expression (Figure 7; Supplementary Table S3). In at least 16 of the 21 species analyzed, one or more transcriptional regulator genes were found in proximity to *ohyA*, suggesting a common regulatory adaptation to specific environmental conditions.

In *S. aureus*, the regulation of *ohyA* expression is more complex. While a transcriptional regulator gene is located near *ohyA*, it does not appear to modulate *ohyA* expression directly. The *S. aureus* transcriptional regulator that flanks *ohyA* is *norG* (SA0104/SAUSA300_0110), a regulator for several efflux pumps (Truong-Bolduc et al., 2011). A microarray screen of *S. aureus* genes regulated by *norG* did not find ohyA (Truong-Bolduc et al., 2011). Regulation by a tetracycline repressor family fatty acid efflux pump transcriptional regulator FarR (SA2340/SAUSA300_2490 and not encoded among the *ohyA* flanking loci) has also been considered, but the regulatory stimulus (unsaturated fatty acid) does not robustly impact *ohyA* expression (Subramanian et al., 2019). Instead, *ohyA* expression is controlled, at least in part, by the SrrAB two-component system, which is conserved across *Staphylococcus* species. A microarray screen of *S. aureus* genes found 8.3-fold higher levels of *ohyA* RNA transcripts in wildtype cells compared to a double knockout Δ*srrAB* strain under nitrosative stress conditions (Kinkel et al., 2013). The SrrAB system is known to regulate genes involved in anaerobic metabolism and oxidative stress response (Kinkel et al., 2013; Jenul and Horswill, 2019; Qiu et al., 2021), linking *ohyA* expression to broader stress response pathways.

**FIGURE 8:**
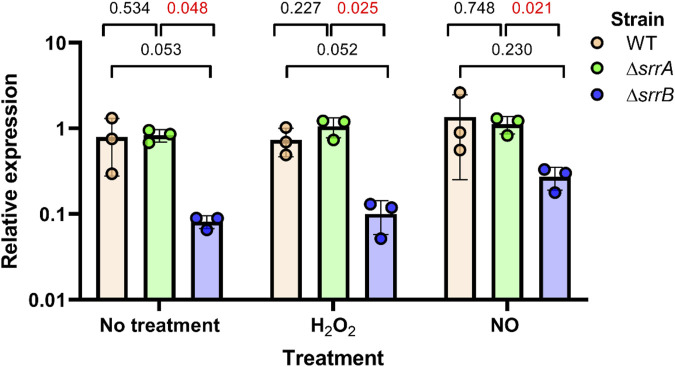
Impact of the SrrAB two component system on *ohyA* expression. The *ohyA* expression levels were quantified by qRT-PCR in the parent/wildtype *Staphylococcus aureus* strain (WT, *orange*), Δ*srrA* knockout strain (*green*), and Δ*srrB* knockout (*blue*). Strains were treated with 5 mM H_2_O_2_ (hydroxy peroxide), 5 mM NO (nitric oxide), or nothing (no treatment). Unpaired t tests with Welch’s correction determined if differences were statistically significant and determined the two-tailed *p*-value. Data are plotted as mean ± SD.

We used qRT-PCR to analyze *ohyA* gene expression in Δ*srrA* or Δ*srrB* single knockout strains to examine the impact of each component under oxidative (hydrogen peroxide, H_2_O_2_) and nitrosative (nitric oxide, NO) stress conditions (Figure 8). In untreated cells or under nitrosative stress, the Δ*srrB* strain showed 7.3-10.5-fold lower *ohyA* expression than the wildtype or Δ*srrA* strains, but was only significantly different from the expression level in the Δ*srrA* strain. When the cells were under oxidative stress, *ohyA* expression in the Δ*srrB* strain was significantly 4.1-5.0-fold lower than wildtype and Δ*srrA* levels. This regulatory network may reflect the importance of *ohyA* in helping *S. aureus* adapt to hostile environments, such as those encountered during infection, where oxidative stress and limited oxygen availability are common challenges.

The presence of conserved regulatory elements across *Staphylococcus* species suggests that *ohyA* regulation has been maintained despite the loss of the gene in many species. This conservation implies that *ohyA* plays a critical role in the species that retain it, possibly conferring a selective advantage in specific ecological niches. Understanding the regulatory mechanisms that control *ohyA* expression can provide valuable insights into how bacteria adapt to environmental stresses and how gene regulation evolves in response to changing selective pressures.

## 4 Discussion

The evolution of the OhyA protein family represents a fascinating case of molecular convergence and divergence, where enzymes that catalyze the same biochemical reaction have evolved different structural and functional features. The two distinct clades of OhyA identified in Bacillales reflect independent evolutionary paths that have led to the emergence of enzymes with unique domain architectures and catalytic mechanisms. This divergence highlights the plasticity of protein evolution, where different evolutionary solutions can arise to meet similar functional demands.

Convergent evolution is a powerful process that underscores the adaptability of biological systems, particularly when organisms face similar environmental challenges. The OhyA phylogenetic tree does not align with the species tree of Bacillales, indicating that the two clades evolved independently. This finding supports the idea of convergent evolution at the molecular level, where similar selective pressures led to the evolution of analogous biochemical functions in distinct evolutionary lineages.

Proteins evolve over time by acquiring and losing functions as mutations subtly alter the shape and electrostatics of their interaction interfaces. A widely accepted hypothesis suggests that ancestral proteins had broad substrate recognition capabilities, while modern proteins arose from gene duplications of these ancestors, followed by fine-tuning of substrate specificity over time (Siddiq et al., 2017). Oleate hydratase shares structural similarities with several flavoenzymes, including monoamine oxidase, tetracycline 7-halogenase, and tryptophan 5-halogenase, yet it uniquely binds unsaturated fatty acids (Hagedoorn et al., 2021). These homologous enzymes differ in substrate specificity due to numerous amino acid variations, and attempts to shift specificity by swapping a few key residues often fail (Gerlt and Babbitt, 2009). Rather than conferring new functions, such modifications tend to disrupt existing biochemical activities. This challenge is evident even within the oleate hydratase family: when catalytic residues of a clade II homolog were replaced with those from clade I, enzyme activity was diminished (Lorenzen et al., 2018). These findings suggest that the divergence in the active sites of clade I and II homologs is the result of long-term sequence changes and complex epistatic interactions. Despite these differences, both clades converged on the ability to catalyze the formation of hydroxy fatty acids.

Clade I enzymes, averaging 591 amino acids in length, feature a three-domain structure that includes a membrane binding domain (Figure 5), indicating a specialized adaptation for interacting with membrane-bound substrates. The presence of this domain suggests that these enzymes are specifically designed for direct membrane association. In contrast, clade II enzymes, which lack the membrane binding domain, likely use alternative mechanisms to access their substrates. The structural simplification of clade II enzymes could reflect an adaptation to environments where direct membrane association is less critical through additional binding partners or a fundamentally different mode of contacting the membrane.

Clade II enzymes averaging 566 amino acids in length possess an amino terminal alpha helix with two segments of positively charged residues flanked by hydrophobic residues. This structure may facilitate membrane interaction, at least in part. However, another subset of clade II enzymes, with an average length of 526 amino acids, lacks this amino terminal helix. The 40-amino acid difference between these two clade II groups likely accounts for the absence of the helix, consistent with predictions generated by AlphaFold.

The membrane binding domain does not determine the phylogenetic relationships among OhyAs, as the grouping of OhyAs into clades remains consistent even after removing the clade I membrane binding domain from the alignment (Supplementary Table S2A). Pairwise structural comparisons of AlphaFold-generated predictions, using sequences excluding the clade I membrane binding domain, were performed with PDBeFOLD. These comparisons demonstrated that the main clade populations are preserved and do not converge (Supplementary Table S2B).

The convergent evolution of OhyA in Bacillales is particularly striking given the low sequence similarity between clade I and clade II enzymes (Section 3.1). Despite these differences, the core catalytic domain structure is highly conserved, underscoring the functional constraints that have shaped the evolution of this protein family. The presence of similar catalytic residues and protein folds across clades suggests that certain structural features are essential for the enzyme’s activity, limiting the range of viable evolutionary solutions.

Our study also highlights the importance of gene regulation in the evolution of the OhyA protein family. The conserved regulatory systems across *Staphylococcus* species, coupled with the presence of additional regulatory elements in some species, indicate that gene regulation has played a key role in the evolutionary success of OhyA. The ability to fine-tune *ohyA* expression in response to environmental conditions likely provides a selective advantage, allowing bacteria to optimize their metabolic responses to varying nutrient availability.

Interestingly, 17/47 mammal-associated *Staphylococcus* species lost *ohyA*, suggesting that ecological niche is not the primary selective pressure favoring the retention of this gene. Furthermore, deletion of *ohyA* from the *S. aureus* genome does not produce an appreciable phenotype *in vitro* (Subramanian et al., 2019), indicating there is not a central metabolic need for OhyA in *Staphylococcus*. The loss of *ohyA* in mammal-associated species may reflect a reduced need for unsaturated fatty acid detoxification in the mammalian host environment, where only a few members of a polymicrobial community may need to carry out this function.

The evolutionary history of OhyA in Bacillales provides a valuable model for understanding the broader principles of protein evolution, particularly in the context of molecular convergent evolution. The independent emergence of similar enzymatic functions in distinct evolutionary lineages illustrates the power of natural selection to shape convergent evolutionary outcomes. At the same time, the divergence in domain architecture and regulatory mechanisms within the OhyA family demonstrates the flexibility of evolutionary processes, where multiple pathways can lead to similar functional results.

In conclusion, the study of OhyA evolution offers insights into the complex interplay between gene divergence and convergent evolution in shaping the diversity of protein functions. The multilevel analysis presented here—from sequence conservation and structural predictions to phylogenetic relationships and regulatory mechanisms—provides a comprehensive understanding of how gene evolution occurs within a protein family. As we continue to uncover the molecular underpinnings of convergent evolution, the OhyA protein family will remain a key example of how evolutionary processes drive the diversification and adaptation of biological systems.

## Data Availability

The original contributions presented in the study are included in the article/Supplementary Material, further inquiries can be directed to the corresponding author.

## Supplementary material

SUPPLEMENTARY FIGURE S1 Comparison of *Staphylococcal* phylogenetic trees using phylo.io. Phylogenetic tree based on *Staphylococcal* 16S rRNA sequences **(A)** compared phylogenetic tree based on *Staphylococcal ohyA* gene **(B)**. Topological similarity between the two trees is quantified on a scale from 0 to 1, where 0 indicates maximum similarity and 1 indicates minimum similarity. Tree branches are color-coded with a gradient to represent the similarity scores for each branch.

SUPPLEMENTARY FIGURE S2 The membrane binding sequence does not determine OhyA clade classification. *A*, Phylogram with outgroup rooting derived from 145 OhyA amino acid sequences, excluding the amino acids that comprise the membrane binding domain in clade I OhyAs. The total length of the multiple sequence alignment is 605 residues. The phylogram reveals two clades: clade I (red shading) and clade II (blue shading), consistent with those shown in Figure 3. Bootstrap values supporting the nodes of the consensus tree are represented by spheres. The coloration on the crust of the tree indicates the distribution of species belonging to the four major *ohyA* genera, as labeled next to the tree. *B*, Statistical evaluation of alignment quality to Clade I *Staphylococcus aureus* OhyA using PDBeFOLD. AlphaFold models for all Bacillales OhyA were employed for alignment. The distinct separation between clade I and Clade II models suggests significant structural differences between the two clades. Dotted lines indicate the median and quartiles.

## Abbreviations

OhyA, oleate hydratase; HFam, homologous family; NAD(P)H, nicotinamide adenine dinucleotide (phosphate); FAD, flavin adenine dinucleotide.
