# Supplementary material for: Independent evolution of oleate hydratase clades in Bacillales reflects molecular convergence
Source: Front Mol Biosci. 2024 Dec 12;11:1485485. doi: 10.3389/fmolb.2024.1485485 (PMC11669549; doi:10.3389/fmolb.2024.1485485)
Supplement: Supplementary file 4 [file DataSheet2.docx]

>Amphibacillus cookii-----------------------------------------MYYSNGNYEAFARPKKPKDVDKKSAYLIGSGLASLSAAAFLIRDGQMKGENIHIFEALDIGGGGLDGIYNNDRGFIIRGGREM-EDHFECLWDLFRSIPSLEIDGA-SVLDEFYWLNKEDPNYSLQRAIVNRGEDAHTDGKFTLSQTASMELIQLFFTPEDKLEKLKITDVLS--EDFFESNFWLYWQTMFAFEPWHSAMEMRRYLARFIHHIGGLPDFSALKFTKYNQYESLALPLITFLENHGVTFQYDTTVTQVEFAITHNKKVAKTIVYQQHN-EEKHLSLTEDDFVFITNGSNTESSTLGDNHTPAAI-----NHDL--GGSWELWKNIAKQDPA-FGSPEIFCGNIAESNW-ESATITTLDDRI-PTYIEKICKRDPFSGKVVTGGIVTIKDSNWLMSFTLNRQPHFKDQPADQLVVWVYSLYCDVPGNYIKKPMKDCTGIEITEEWLYHLGVPEVEIHDMATH-AANCIPCMMPFVTSYFMTRSAGDRPNVVPEGCVNAAFIGNFAETPRDTVFTTEYSVRTAMEAVYTLLNIDRGVPEVFASAYDIRVLLDSTAKMLDGQKLTDL-ALPDETKQIINKGLNRMSDTEVIRLLKEYQLL------------------------

>Amphibacillus jilinensis

-----------------------------------------MYYSNGNYEAFARPKKPKDVDKKSAYLIGSGLASLSAAAFLIRDGQMRGENIHIFEELDIGGGGLDGIYNGDRGFIIRGGREM-EDHFECLWDLFRSIPSLEIDGA-SVLDEFYWLNKEDPNYSLQRAIVNRGEDAHTDGKFTLSQTASMELIQLFFTPEDKLEKLKITDVLS--EDFFESNFWLYWQTMFAFEPWHSAMEMRRYVARFIHHIGGLPDFSALKFTKYNQYESLALPLITFLENHGVTFQYDTTVTQVEFAITHNKKVAKTIVYQQHN-EEKQLSLAEDDFVFITNGSNTESSTLGDNHTPATI-----NHDL--GGSWELWKNIAKQDPA-FGSPETFCGNIAESNW-ESATITTLDDRI-PAYIEKICKRDPFSGKVVTGGIVTIKDSNWLMSFTLNRQPHFKDQPADQLVVWVYSLYCDVPGNYIKKPMKDCTGIEITEEWLYHLGVPEVEIHDMATH-AANCIPCMMPFVTSYFMTRSAGDRPNVVPEGCVNAAFIGNFAETPRDTVFTTEYSVRTAMEAVYTLLNIDRGVPEVFASAYDIRVLLDSTAKMLDGQKLTDL-ALPDETKQIINKGLNRMSDTEVIRLLKEYQLL------------------------

>Bacillus marinisedimentorum

---------------------------------------------MGSYERI-NPVKPKGIENKKAYLIGGGIASLAAAEYLIRDGHMDGKQITILEQDHVIGGALDGSGNAEDGYLARGGREM-EEHYECVWDLFGGVPSLEDPER-TVLEEFRELNIVDPNYSNCRAIANRGE-KLDFSSLGLAEHHVKQLTKLFLATEESLGAATVEQFFD--DSFLETDMWLYWRSMFAFEPWHSVVEMKRYMHRFIHLMPGMSRMEGLVFTKYNQYDSMVLPLKKSLESQGVVFDLNTQVTDLDIDIAGNKKTVTGIHLTRDGKKQEVIKTTENDLVFFTNGSMTENSTLGSMDKAPVL-----DKSD--GGSWSLWKKIAEKDAS-FGNPEVFCGDIEKSKW-ESYTITARGPKM-RELVEKFAERKIVPHRTVTGGIITVKDSSWLLSVTVNRQPQFLDQPKDVIVLWAYGLFPDNEGDFIKKRMSDCTGRELLQELLYHLGIDEKDMQEYIDT--SIVIPSMMPYITSQFMPRVKGDRPQVVPEGSANLAFLGQFAEIEGDCVFTVEYSVRSAMIAVYTLLGLEKNPPEIYPSQYDIRVIANAVKTLNSGRPLP-----------AEPIIKKLLSNTSLEGLI------------------------------

>Bacillus massiliigorillae

-----------------MKKRTILLAAGTTLGAAYVVNKKRKQKAIEKEQQVDEEISSRYYGDKQVYFIGGGIASLAGAAYLIRDAHFNGENIHIIEGMHILGGSNDGAGTPETGFVVRGGRMLNEETYENFWELFGSIPSLDMPGQ-SVTEEILNFDHLHPTHAQARLVDKDRN-IINTHSMGFNNSDRLAMTRLLATPEEKLDDMTIQDWFE--PHFFETNFWYMWQTTFAFQKWSSLFEFRRYMNRMMLEFSRIDTLEGVTRTRFNQYESLILPLKAYLDNHNVDFTINQTVTDIDFK-DDSNITATAIHLSNGT----IIELGEEDVVIMTNACMTDSATLGDLHTPAPK-----PVER--PISGELWYKVAKKKPN-LGNPEPFFNHENETNW-QSFTVTCQGDAL-LKRIEQFTGNIPG-----SGALMTFKDSNWLMSTVVAAQPHFKAQDANTTIFWGYGLYPDRIGDYVKKPMKECSGEEILYELICHLKW-DEDWEEIKKD-VINVIPCYMPYIDAQFQPRKMSDRPKVVPEGSTNFAMISQFVEIPDDMVFTEEYSVRAARIAIYTLFNIDKEICPVTPYNRNPQVLAKATKTMFR----------------------------------------------------------------

>Bacillus massilioanorexius

----------------MKKRDIGVIAAGTAIGAAFAVKKVNERKHKNKQRLVDEDIQSRNYGNKQVYFIGGGIASLAGAAYLIRDAHFKGENIHIIEGMDILGGSNDGAGSGEKGFVCRGGRMLNEETYENFWDLFSSIPSLEMPEQ-SVTEEILNFDHLHPTHAQARLVDKNCK-IIDAHSMGFNNSDRLAMTRLLATPEEKLDDMTIQDWFD--SHFFETNFWFMWQTTFAFQKWSSLFEFRRYMNRMMLEFSRIDTLEGVTRTRYNQYESLILPLKAFLDCHHVDFTINKVVTDIDFK-EGDSITATVLHLFDEE----TIVLGEEDVVFMTNACMTDSATLGDLHTPAPQ-----PEER--PISGELWYKIAQKKPQ-LGNPEPFFGHENQTNW-QSFTVTCRGDRL-LKRIERFTGNIPG-----SGALMTFKDSNWLMSTVVAAQPHFKAQDANTTIFWGYGLYPNRIGDYVKKPMKECTGEEILYELICHLKW-ADDWEDIKKD-VINVIPCYMPFIDAQFQPRKMSDRPKVVPDGSTNFAMISQFVEIPQDMVFTEEYSVRAARIAVYTLFDIDKEICPVTPYHRDPKVIAKATKTMFR----------------------------------------------------------------

>Fontibacillus panacisegetis

-------------------------------------------------------MVKQENGKSQVYFVGGGIASLAGAAYLVRDCDFPGQNIHIIEEMKILGGSNDGAGDEEHGYVIRGGRMLNDETYENLWELLMTIPSLDHPDR-SVREEITEFDNANPTHSSARLVNRQGE-VVDVLSMGFDMADRLAMGKLIVTPEERLGKARINEWFG--PHFFTTNFWYMWATTFAFQPWHSAVEFKRYMIRFMHEFPRIQTLEGVTRTPYNQYDSIILPLHKYLESYGVDFAMKCTVTDLQFK-EGDGITVTQMNISQEG-EGSVIPVHEGDLVIVTNGSMTESSSLGSM-TEAPK-----MNGK--GSSWKLWDRISAKKTG-LGNPSSFDDHANESKW-ESFTVTFQDSVF-FDLMEKFSRNRAG-----TGALVTFKDSSWLMSIVLAFQPHFRNQPDHVKVFWGYGLYPDNVGDFVKKKMCDCTGEEIMAELLYHLHF-EEHKDAIMAT--ANCIPCMMPYITSQFMPRLGSDRPKVVPEGSTNLAFIGQFCEIPDDVVFTEEYSVRAARIAVYTLLGIDKSIAPINQYQYDVRTLLAGLVTSFR----------------------------------------------------------------

>Fontibacillus phaseoli

--------------------------------------------------------MTVDVKDRQVYFVGGGIASLAGAAFLVRDCDFPGHQIHIIEEMKILGGSNDGAGNEVQGYVIRGGRMLNDETYENTWDLLKCIPSLDDPQV-SVRDEIVAFDTAHPTHSNARLVNKEGQ-VEDVTSMGFDMADRLAMAKLIITPEDKMGKARINDWFG--PHFFETNFWFMWATTFAFQPWHSAVELKRYMIRFMHEFPRIHTLEGVTRTPYNQYDSIILPLHKYLEGHGVDFEMKCTVTDLDFK-DGDGITVTRMHYVKDG-AEKALDLSGNDLVIITNGSMTESSSLGSM-TTAPV-----LNGK--GSSWKLWDTIAAKKSG-LGNPSSFDDHIDGSKW-ESFTVTCQDSRF-FDLMEAFSRNKAG-----TGALVTFKESSWLMSIVLAHQPHFRNQPEHVKVFWGYGLYPDRVGDFVKKKMSDCTGEEILTELLHHLHF-EKDMDAIIAS--ANCIPCMMPFITAQFMPRAVSDRPKVVPEGSTNLAFISQFCEIPDDVVFTEEYSIRAARIAVYTLLGMDKPISPINQYHYDVRTLLQGFVTSFR----------------------------------------------------------------

>Fontibacillus solani

--------------------------------------------------------MTVDVKDRQVYFVGGGIASLAGAAFLVRDCDFPGHQIHIIEEMKILGGSNDGAGNEVQGYVIRGGRMLNDETYENTWDLLKCIPSLDDPQV-SVRDEIVAFDTAHPTHSNARLVNKEGQ-VEDVTSMGFDMADRLAMAKLIITPEDKMGKARINDWFG--PHFFETNFWFMWATTFAFQPWHSAVELKRYMIRFMHEFPRIHTLEGVTRTPYNQYDSIILPLHKYLEGHGVDFEMKCTVTDLDFK-DGDGITVTRMHYVKDG-AEKALDLSGNDLVIITNGSMTESSSLGSM-TTAPV-----LNGK--GSSWKLWDTIAAKKSG-LGNPSSFDDHIDGSKW-ESFTVTCQDSRF-FDLMEAFSRNKAG-----TGALVTFKESSWLMSIVLAHQPHFRNQPEHVKVFWGYGLYPDRVGDFVKKKMSDCTGEEILTELLHHLHF-EKDMDAIIAS--ANCIPCMMPFITAQFMPRAVSDRPKVVPEGSTNLAFISQFCEIPDDVVFTEEYSIRAARIAVYTLLGMDKPISPINQYHYDVRTLLQGFVTSFR----------------------------------------------------------------

>Gemella bergeri

--------------------------------------------------------MTVDVKDRQVYFVGGGIASLAGAAFLVRDCDFPGHQIHIIEEMKILGGSNDGAGNEVQGYVIRGGRMLNDETYENTWDLLKCIPSLDDPQV-SVRDEIVAFDTAHPTHSNARLVNKEGQ-VEDVTSMGFDMADRLAMAKLIITPEDKMGKARINDWFG--PHFFETNFWFMWATTFAFQPWHSAVELKRYMIRFMHEFPRIHTLEGVTRTPYNQYDSIILPLHKYLEGHGVDFEMKCTVTDLDFK-DGDGITVTRMHYVKDG-AEKALDLSGNDLVIITNGSMTESSSLGSM-TTAPV-----LNGK--GSSWKLWDTIAAKKSG-LGNPSSFDDHIDGSKW-ESFTVTCQDSRF-FDLMEAFSRNKAG-----TGALVTFKESSWLMSIVLAHQPHFRNQPEHVKVFWGYGLYPDRVGDFVKKKMSDCTGEEILTELLHHLHF-EKDMDAIIAS--ANCIPCMMPFITAQFMPRAVSDRPKVVPEGSTNLAFISQFCEIPDDVVFTEEYSIRAARIAVYTLLGMDKPISPINQYHYDVRTLLQGFVTSFR----------------------------------------------------------------

>Gemella morbillorum

-----------------------------------------MYYSNGNYEAFAKPKKPAGVDKKSAYLVGSGLASLAAAAFLVRDGQMKGERIHILEELPIAGGSLDGIMNPTRGFIIRGGREM-EDHFECLWDLFRSIPSLEVEDA-SVLDEFYWLNKEDPNFSKCRVIENRGQQIPNDGLFALSDKSSQELVKLYLTSESELQGMKITDFFS--SEFFESNFWTYWATMFAFEKWHSVAEMRRYMLRFIHHIKGLPDLSALKFTKYNQYESLVLPLVKYLESHGVTFEYNTVVKDIKVEKQGKDLVAKHLILEVNG-EPVVRELTEDDLVFVTNGSITASTTYGNNDTSAPV-----SKEL--GGAWQLWKNLAKQDER-FGRPEVFCENLPDQSWFVSATTTVTDKRI-AEYIEKICKRDPYAGKVVTGGIVTARDSNWMMSFTLNRQPHFKSQSKDELVVWIYGLYSNISGNYIKKPIEACTGIEIAEEWLYHIGVPEQFIHEFASK-GCSTVPCYMPYITSYFMPRHDGDRPLVIPEGSKNLAFIGNFSETPRDTVFTTEYSVRTAMEAVYTLLDVDRGVPEVFNSAYDLRVMTKSTNRLMDGKKLEEV-KLPFFAKAISKRVLKKIKGTYIEEILKDAGLI------------------------

>Gemella sanguinis

-----------------------------------------MYYSNGNYEAFAKPKKPAGVDKKSAYLVGSGLASLSAAAFLVRDGQMKGERIHILEELPIAGGSLDGIMNPTRGFIIRGGREM-EDHFECLWDLFRSIPSLEVEDA-SVLDEFYWLNKEDPNYSKCRVIENRGQQIPDDGLFALSDKSSQELVKLYLTSESELQGMKITDFFS--SEFFESNFWTYWATMFAFEKWHSVAEMRRYMLRFIHHIKGLPDLSALKFTKYNQYESLVLPLVKYLESHGVTFEYNTVVKDIKVEKQGKDLVAKHLILEVNG-EPVVRELTEDDLVFVTNGSITASTTYGNNDTSAPV-----SKEL--GGAWQLWKNLAKQDER-FGRPEVFCENLPDQSWFVSATTTVTDKRI-AEYIEKICKRDPYAGKVVTGGIVTARDSNWMLSFTLNRQPHFKSQSKDELVVWIYGLYSNISGNYIKKPIEACTGIEIAEEWLYHIGVPEQFIHEFASK-GCSTVPCYMPYITSYFMPRHDGDRPLVIPEGSKNLAFIGNFSETPRDTVFTTEYSVRTAMEAVYTLLDVDRGVPEVFNSAYDLRVMTKSTNRLMDGKKLEEV-KLPLFAKAISKRVLKKIKGTYIEEILKDAGLI------------------------

>Gorillibacterium timonense

--------------------------------------------------------MTHDRGTKQVYFVGGGLASLAGAVYLIRDGGMDGKSIHILEGLPIVGGSNDGIGTPQKGFVARGGRMLNEETYENFWELMYSIPSIHQPGK-SVSEEILEFDHAHPTHANARLIDKDGQ-VLDVTSMGFSMQDRMALLKLVRTPEEKLDNLKISDWFS--PHFFETNFWYMWQTTFAFQEWSSLFELRRYMNRMIFEFSRIHTLEGVTRTPYNQYDSVILPLKAYLDKHGVDFSLHCRVTDFDFA-EGEGITVTAIHYEDLN-GKGIIPLKEGDLCIATNASMTDSATLGDLHTSAPF-----LPER--PLSGDLWANIAAKKAPHLGDPKPFFGNPEETNW-TSFTVTMQGNKL-LKKIEQFSRNVPG-----SGALMTFKDSSWRMSIVVAAQPHFINQPADQTIFWGYGLYTDKLGDYVQKPMRDCTGEEMLIELLHHLHW-EDELDAILKD-VINVIPCNMPYIDAQFQPRKMTDRPKVVPEGSTNLALISQFVEIPEDMVFTEEYSVRAARIAVYTLMGIKRPIAPVTPYQHDVRVLLKAVNTSFR----------------------------------------------------------------

>Kurthia gibsonii

-----------------------------------------MYRSNGNYEAFARPRKPERADRVSAYLVGSGLASLAAAAFLVRDGQVPGERIHILEELSIPGGSLDGIDRPDAGFVIRGGREM-ESHFECLWDLFRSVPSLEVENA-SVLDEFYWLNKDDPNSSHCRIIHKKGERYPTDGQLTLSEKSVKEILHLCLQKEENLQNKKITDVFS--KEFFESNFWTYWCTMFAFEEWHSAMEMRRYLMRFIHHIDALADFSSLKFTKYNQYESLVLPLMNYLKEQGVHFQYDTVVKNVMMSKGGLEKVAKELHLTVKG-EEQVVQLSEDDLVFVTNGSITDSSTYGTQDEPAPP-----TYKT--SGGWDLWKNLAAQDAD-FGYPEKFYKNIPKASWFVSATVTTLDDKV-APYIERISKRDPYAGKVVTGGIVHAKDSNWKMSYTLNRQPHFKTQPKDELVVWVYALISDQPGNYIKKPITECTGMEIACEWLYHMGVPVNEIEDLARN-SCNTVPCYMPYITSYFMPRALGDRPLVVPKGSKNLAFIGNFAETERDTVFTTEYSVRTAMEAVYKFLDIERGVPEVFASSYDIRMLLRASYYLNDKKGIRDM-KVPILEGMLAKHELKKIKDTFIEELLEDSGLL------------------------

>Kurthia sibirica

-----------------------------------------MYRSNGNYEAFARPKKPERADKVSAYLVGSGLASLSAAVFLIRDGQVKGDKIHILEELPIAGGSLDGLSRPDLGFVIRGGREM-ESHFECLWDLFRSIPSIEVENA-SVLDEFYWLNKEDPNSSECRIIHKQGVQFPTDGKLMLSEKAIKEMLALCLHQEDQLADKKITDVFT--EEFFKSNFWTYWCTMFAFEEWHSAMEMRRYLMRFIHHIDALTDFSSLKFTKYNQYESLVLPLLKYLKEQGVQFQYDTVVENVIVD-SGPTKVAKELILKVKG-EAQTIALTEHDLVFVTNGSITDSSTYGSNEEPAPV-----STET--SGGWDLWENLAEQNAE-FGRPEKFYKNIPRASWFVSATVTTLDDKI-APYIERISKRDPYAGKVVTGGIVHAQDSNWKMSYTLNRQPHFKAQRKDELVVWVYALLSDKEGDYIKKPITQCSGNEIASEWLYHMGVPIELIEGLAVN-SCSTIPCYMPYITSYFMPRAVGDRPLVVPHGSKNLAFIGNFAETERDTVFTTEYSVRTAMEAVYTFLDIDRGVPEVFASSYDIRMLLKASYYLNDKKGIKDI-KVPMLAGIIERHELKKIQGTFIEELLKDSGLL------------------------

>Listeria cossartiae

---------------MKNKKRTLVALTGAAIGTGIAAKKISEQKAAEKERAVDEAIKARYYGDKQVYFVGGGIASLAGAVYLIRDANFDGKNIHIIEGMHILGGSNDGAGSVEHGFVCRGGRMLNEETYENFWDLFSSIPSLDMPNF-SVTEEILNFDHLHPTHAQARLVDKDRN-ILDAHSMGFNNNDRMLMTKLLATPEEKLDNLTIRDWFD--EHFFETNFWYMWQTTFAFQKWSSLFEFRRYMNRMMLEFSRIDTLEGVTRTPLNQYESLILPLKTFLDKHHVDFTINQTVEDIDFK-DAPGITATALHLSDGS----TIELGPDDDVIMTNACMTDSATLGDMNTPAPK-----PEEK--PISGELWYKVAQKKPN-LGNPEPFFAHEEETNW-QSFTVTCHGDKL-LKRIERFTGNIPG-----SGALMTFKDSNWLMSTVVAAQPHFKAQDANTTIFWGYGLYPDRVGDFVKKPMKECTGEEILYELMCHLNW-QDDFEEIKAD-IINVIPCYMPYIDAQFEPRAMSDRPAVVPEGSTNFAMISQFVEIPKDMVFTEEYSVRAARIAVYTLLDIDKKICPVTPHNRDPKVLAKATQTMFR----------------------------------------------------------------

>Listeria costaricensis

---------------LMMKKRNLVVAAGAAAGAFYTVKKVSENKAGVKAEKVDEAIQERFYGDKQVYFIGGGIASLAGAAFLIRDAHFPGKNIHVIEGLEVLGGSNDGSGTVQEGFLCRGGRMLNEETYENFWDLYGSIPSLEIPGQ-SVTEEILNFDHLHPTHAQARLVDKNQQ-IQNAHSMGFSTQDRYEMTKLLATPEERLDDMRINEWFN--DHFFETNFWYMWQTTFAFQKWSSLFEFRRYMNRMMLEFSRIDTLEGVTRTRFNQYESLILPLKAFLEKNDVDFMTNTVVSDIDFK-EGAGITASVLYFADGG----KLELKDGDQVIMTNACMTDSATIGDFSTPAPK-----PEER--PISGELWYKVAQKKPG-LGNPEPFFGHEDETNW-QSFTVTAKGDAL-LKRIERFSGNVPG-----SGALLTLKDSNWLMSSVVAAQPHFKSQPLDTTIFWGYGLYPDRIGDYVKKPMKDCTGEEILFEYISQLGW-QDDWEEIKKD-IVNVIPCYMPYIDAQFQPRKMSDRPSVVPEGSTNFAMISQFVEIPKDMVFTEEYSVRAARIAVYTLFDIDKEIKPVTPYNRDPKVLAKATQTMFR----------------------------------------------------------------

>Listeria farberi

---------------MKNKKRTLVALTGAAIGTGIAAKKISEQKAAEKERAVDEAIKARYYGDKQVYFVGGGIASLAGAVYLIRDANFDGKNIHIIEGMHILGGSNDGAGSVEHGFVCRGGRMLNEETYENFWDLFSSIPSLDMPNF-SVTEEILNFDHLHPTHAQARLVDKDRN-ILDAHSMGFNNNDRMLMTKLLATPEEKLDNLTIRDWFD--EHFFETNFWYMWQTTFAFQKWSSLFEFRRYMNRMMLEFSRIDTLEGVTRTPLNQYESLILPLKTFLDKHHVDFTINQTVEDIDFK-DAPGITATALHLSDGS----TIELGPDDDVIMTNACMTDSATLGDMNTPAPK-----PEEK--PISGELWYKVAQKKPN-LGNPEPFFGHEEETNW-QSFTVTCHGDKL-LKRIERFTGNIPG-----SGALMTFKDSNWLMSTVVAAQPHFKAQDANTTIFWGYGLYPDRVGDFVKKPMKECTGEEILYELMCHLNW-QDDFEEIKAD-IINVIPCYMPYIDAQFEPRAMSDRPAVVPEGSTNFAMISQFVEIPKDMVFTEEYSVRAARIAVYTLLDIDKKICPVTPHNRDPKVLAKATQTMFR----------------------------------------------------------------

>Listeria grayi

-----------------------------------------MRYTNGNYEAFARSRKPAGVEEKSAYIVGGGLAGLAAAAFLIRDGHMPGNQIRILEELPVAGGSLDGKVVEHEGLVTRGGREM-EAHFECLWDLFRSIPSLEVEDA-SVLDEFYWLDLDDPNSSNCRMIHNRGERVPDDGKFTLSKDAQKEIISLVMATEEKLEGKRIEDVFG--EAFFASNFWLYWCSMFAFEKWHSAIEMRRYLLRFIHHIEGLPDFTALKFTRYNQYESLVKPLLAYLKNHGVSFQYDVQVNDITVEVAAESKTARRLLITRNG-QGEEIPLTEKDLIFVTNGSITESSTQGNHHTPAPI-----SHDL--GGSWTLWKNLAKQSPE-FGHPEVFYENLPEESWFVSATITWENDAI-APYIERLTQRKLRTGKIVSGGIITIKDSNWLMSFATHRQPHFKEQKDNQTVTWVYGLLSNTPGNYIKKPIEACSGEEIVQELLYHLGVPEVEISHMANEDSCKVVPVYMPFITSYFMLRKQGDRPLVVPHGSKNLAFIGNFAETERDTVFTTEYSVRTAMEAVYQLLNVERGVPEVFASSYDIRKLTNAAYFLSDQKKLVDM-DLPFIERKVIERMAKKNKDTFIGEILRDAKLI------------------------

>Listeria innocua

---------------MKNKKRTLVALTGAAIGTGIAAKKISEQKAAEKERVVDEAIKARYYGDKQVYFVGGGIASLAGAVYLIRDANFDGKNIHIIEGMHILGGSNDGAGSVEHGFVCRGGRMLNEETYENFWDLFSSIPSLDMPNF-SVTEEILNFDHLHPTHAQARLVDKDRN-ILDAHSMGFNNNDRMLMTKLLATPEEKLDNLTIRDWFD--EHFFETNFWYMWQTTFAFQKWSSLFEFRRYMNRMMLEFSRIDTLEGVTRTPLNQYESLILPLKTFLDKHHVDFTINQTVEDIDFK-DAPGITATALHLSDGS----TIELGPDDDVIMTNACMTDSATLGDMNTPAPK-----PEEK--PISGELWYKVAQKKPN-LGNPEPFFGHEEETNW-QSFTVTCHGDKL-LKRIERFTGNIPG-----SGALMTFKDSNWLMSTVVAAQPHFKAQDANTTIFWGYGLYPDRVGDFVKKPMKECTGEEILYELMCHLNW-QDDFEEIKAD-IINVIPCYMPYIDAQFEPRAMSDRPAVVPEGSTNFAMISQFVEIPKDMVFTEEYSVRAARIAVYTLLDIDKKICPVTPHNRDPKVLAKATQTMFR----------------------------------------------------------------

>Listeria ivanovii

---------------MKNKKRTVVALTGAAIGASVVAKKISEQKAVEKERLVDEAIQARYYGDKQVYFVGGGIASLAGAVYLIRDANFDGKNIHIIEGMHILGGSNDGAGSVDKGFVCRGGRMLNEETYENFWDLFSSIPSLDMPNF-SVTEEILNFDHLHPTHAQARLVDKDRN-ILDAHSMGFNNNDRMLMTKLLATPEEKLDHLTIRDWFD--EHFFETNFWYMWQTTFAFQKWSSLFEFRRYMNRMMLEFSRIDTLEGVTRTPLNQYESLILPLKTFLDKHHVDFTINQTVEDIDFK-DAPGITATALHLSDGT----VINLGPDDDVIMTNACMTDSATLGDMNTPAPK-----PEEK--PISGELWYKVAQKKPN-LGNPEPFFGHEEETNW-QSFTVTCNGDKL-LNRIERFTGNIPG-----SGALMTFKDSNWLMSTVVAAQPHFKAQDANTTIFWGYGLYPDRVGDFVKKPMKECTGEEILYELICHLNW-QDDWEEIKAD-IVNVIPCYMPYIDAQFEPRAMSDRPAVVPEGSTNFAMISQFVEIPQDMVFTEEYSVRAARIAVYTLLDIDKKICPVTPHNRNPKVLAKATQTMFR----------------------------------------------------------------

>Listeria marthii

---------------MKNKKRTLVALTGAAIGTGIAAKKISEQKAVEKERAVDEAIKARYYGDKQVYFVGGGIASLAGAVYLIRDANFDGKNIHIIEGMHILGGSNDGAGSVENGFVCRGGRMLNEETYENFWDLFSSIPSLDMPNF-SVTEEILNFDHLHPTHAQARLVDKDRN-ILDAHSMGFNNNDRMLMTKLLATPEEKLDNLTIRDWFD--EHFFETNFWYMWQTTFAFQKWSSLFEFRRYMNRMMLEFSRIDTLEGVTRTPLNQYESLILPLKTFLDKHHVDFTINQTVEDIDFK-DAPGITATALHLSDGS----TIELGPDDDVIMTNACMTDSATLGDMNTPAPK-----PEEK--PISGELWYKVAQKKPN-LGNPEPFFGHEEETNW-QSFTVTCHGDEL-LKRIERFTGNIPG-----SGALMTFKDSNWLMSTVVAAQPHFKAQDANTTIFWGYGLYPDRVGDFVKKPMKECTGEEILYELMCHLNW-QDDFEEIKAD-IINVIPCYMPYIDAQFEPRAMSDRPAVVPEGSTNFAMISQFVEIPKDMVFTEEYSVRAARIAVYTLLDIDKKICPVTPHNRDPKVLAKATQTMFR----------------------------------------------------------------

>Listeria monocytogenes

---------------MKNKKRTLVALTGAAIGTGIAAKKISEQKAAEKERAVDEAIKARYYGDKQVYFVGGGIASLAGAVYLIRDANFDGKNIHIIEGMHILGGSNDGAGSVEHGFVCRGGRMLNEETYENFWDLFSSIPSLDMPNF-SVTEEILNFDHLHPTHAQARLVDKDRN-ILDAHSMGFNNNDRMLMTKLLATPEEKLDNLTIRDWFD--EHFFETNFWYMWQTTFAFQKWSSLFEFRRYMNRMMLEFSRIDTLEGVTRTPLNQYESLILPLKTFLDKHHVDFTINQTVEDIDFK-DAPGITATALHLSDES----TIELGPDDDVIMTNACMTDSATLGDMNTPAPK-----PEEK--PISGELWYKVAQKKPN-LGNPEPFFAHEEETNW-QSFTVTCHGDKL-LKRIERFTGNIPG-----SGALMTFKDSNWLMSTVVAAQPHFKAQDANTTIFWGYGLYPDRVGDFVKKPMKECTGEEILYELMCHLNW-QDDFEEIKAD-IINVIPCYMPYIDAQFEPRAMSDRPAVVPEGSTNFAMISQFVEIPKDMVFTEEYSVRAARIAVYTLLDIDKKICPVTPHNRDPKVLAKATQTMFR----------------------------------------------------------------

>Listeria seeligeri

---------------MKNKKRTVVALTGAAIGTRIAAKKISEQKATEKERLVEEAIQARYYGDKQVYFVGGGIASLAGAAYLIRDANFDGKNIHIIEGMHILGGSNDGAGSTDKGFVCRGGRMLNEETYENFWDLFRSIPSLDMPNF-SVTEEILNFDHLHPTHAQARLVDKDRN-ILDAHSMGFNNNDRMLMTKLLATPEEKLDHLTIRDWFD--EHFFETNFWYMWQTTFAFQKWSSLFEFRRYMNRMMLEFSRIDTLEGVTRTPLNQYESLILPLKTFLDKHHVDFTINQTVEDIDFK-DAPGITATALHLSDGT----VINLGPDDVVIMTNACMTDSATLGDMNTPAPK-----PEEK--PISGELWYKVAQKKPN-LGNPEPFFGHEEETNW-QSFTVTCNGDKL-LKRIERFTGNIPG-----SGALMTLKDSNWLMSTVVAAQPHFKAQDANTTIFWGYGLYPDRVGDFVKKPMKECTGEEILYELMCHLNW-QDDWEEIKAD-IVNVIPCYMPYIDAQFEPRAMSDRPAVVPEGSKNFAMISQFVEIPQDMVFTEEYSVRAARIAVYTLLDIDKKICPVTPHNRNPKVLAKATQTMFR----------------------------------------------------------------

>Listeria swaminathanii

---------------MKNKKRTLVALTGAAIGTGIAAKKISEQKAAEKERAVDEAIKARYYGDKQVYFVGGGIASLAGAVYLIRDANFDGKNIHIIEGMHILGGSNDGAGSVENGFVCRGGRMLNEETYENFWDLFSSIPSLDMPNF-SVTEEILNFDHLHPTHAQARLVDKDRN-ILDAHSMGFNNNDRMLMTKLLATPEEKLDNLTIRDWFD--EHFFETNFWYMWQTTFAFQKWSSLFEFRRYMNRMMLEFSRIDTLEGVTRTPLNQYESLILPLKTFLDKHHVDFTINQTVEDIDFK-DAPGITATALHLSDGS----TIELGPDDDVIMTNACMTDSATLGDMNTPAPK-----PEEK--PISGELWYKVAQKKPN-LGNPEPFFGHEEETNW-QSFTVTCHGDEL-LKRIERFTGNIPG-----SGALMTFKDSNWLMSTVVAAQPHFKAQDANTTIFWGYGLYPDRVGDFVKKPMKECTGEEILYELMCHLNW-QDDFEEIKAD-IINVIPCYMPYIDAQFEPRAMSDRPAVVPEGSTNFAMISQFVEIPKDMVFTEEYSVRAARIAVYTLLDIDKKICPVTPHNRDPKVLAKATQTMFR----------------------------------------------------------------

>Listeria welshimeri

---------------MKNKKRTLVALTGAAIGTGIAAKKISEQKNAEKERLVDEAIKARYYGDKQVYFVGGGIASLAGAAYLIRDANFDGKNIHIIEGMHILGGSNDGAGSVEHGFVCRGGRMLNEETYENFWDLFSSIPSLDMPNF-SVTEEILNFDHLHPTHAQARLVDKDRN-ILDAHSMGFNNNDRMLMTKLLATPEEKLDNLTIRDWFD--KHFFETNFWYMWQTTFAFQKWSSLFEFRRYMNRMMLEFSRIDTLEGVTRTPLNQYESLILPLKTFLDKHHVDFTINQTVEDIDFK-DAPGITATALHLSDGS----TIELGPDDDVIMTNACMTDSATLGDMNTPAPK-----PEEK--PISGELWYKVAQKKPN-LGNPEPFFGHEEETNW-QSFTVTCHGDKL-LKRIERFTGNIPG-----SGALMTFKDSNWLMSTVVAAQPHFKAQDANTTIFWGYGLYPDRVGDFVKKPMKECTGEEILYELMCHLNW-QDDFEEIKAD-IINVIPCYMPYIDAQFEPRAMSDRPAVVPEGSTNFAMISQFVEIPKDMVFTEEYSVRAARIAVYTLLDIDKKICPVTPHNRDPKVLAKATQTMFR----------------------------------------------------------------

>Lysinibacillus agricola

-----------------------------------------MYYSNGNYEAFARPKKPVGVDEKSAYLVGSGLASLSAACFLIRDGQMKGENIHILEELDISGGSLDGILNPTRGFIIRGGREM-EDHFECLWDLFRSIPSLEIENA-SVLDEFYWLNKEDPNYSKCRLMDNRGQRLEDDGKFTLSDKASEEMIKLFFTPEEKLDDKKITDVFS--EEFFESNFWLYWSTMFAFEKWHSAMEMRRYIMRFIHHVGGLPDLSALKFTKYNQYESLVLPMIKYLEGHNVDFQFNTVVENVLVDKVGDKKVAHTLVLRQNG-VKKNIELTENELVFVTNGSITESTTYGDNNTPAPI-----STDL--GGSWSLWKNIASQDSE-FGKPEKFCDNLPEESWFVSATLTTLDAKV-APYIEKISKRDPYAGKVVTGGIVTATDSNWMLSYTLNRQPHFKDQPKDQLVVWIYGLLSNKPGDFIKKSITECSGSEIAQEWLYHMGVPVDEIPDLAQN-SCNTIPCYMPYITSYFMPRAMGDRPLVVPNGSVNLAFMGNFSETERDTVFTTEYSVRTAMEAVYQLLDIDRGVPEVFASTYDIRTLLSSTARLLDGKKLTDV-DAPFILKQIGKFGIHKTKDTIVYDLLKESKLI------------------------

>Lysinibacillus boronitolerans

-----------------------------------------MYYSNGNYEAFARPKKPEGVDGKSAYLIGSGLASLSAACFLIRDGQMKGENIHILEELDISGGSLDGILNPTRGFIIRGGREM-EDHFECLWDLFRSIPSLEVDNA-SVLDEFYWLNKEDPNYSKCRLMKDRGQRLEDDGKFTLSDQSSEEMIKLFFTPEEKLEDKKITDVFS--DEFFESNFWLYWSTMFAFEKWHSAMEMRRYIMRFIHHIGGLPDLSALKFTKYNQYESLVLPMIKYLEGHDVDFQYNTVVENVLVDRVGDKKVAHTLVLRKDG-VKKNIELTENELVFVTNGSITESTTYGDNNTPAPI-----NKDL--GGSWSLWKNIAAQGEE-FGRPEKFCDNLPEESWFVSATLTTLDDRV-APYIEKISKRDPYAGKVVTGGIVTATDSNWMLSYTLNRQPHFKNQPKDQLVVWIYGLLSNKPGDFIKKSITECTGIEIAQEWLYHMGVPVDEIPDIAQN-SCNTIPCYMPYITSYFMPRAIGDRPLVVPEGSTNLAFIGNFSETARDTVFTTEYSVRTAMEAVYQLLNIDRGVPEVFASAFDVRTLLASTARLLDGKKLTDI-DAPFILKQIGKLGIHKTKDTIIYDLLKESKLI------------------------

>Lysinibacillus capsici

-----------------------------------------MYYSNGNYEAFARPKKPEGVDEKSAYLIGSGLASLSAACFLIRDGQMKGENIHILEELDISGGSLDGILNPTRGFIIRGGREM-EDHFECLWDLFRSIPSLEVENA-SVLDEFYWLNKEDPNYSKCRLMKDRGQRLEDDGKFTLSDKSSEEMIKLFFTPEEKLEDKKITDVFS--DEFFESNFWLYWSTMFAFEKWHSAMEMRRYIMRFIHHIGGLPDLSALKFTKYNQYESLVLPMIKYLESHDVDFQYNTVVENVLVDKVGDKKVAHTLVLRKDG-VKKNIELTENELVFVTNGSITESTTYGDNNTPAPI-----NKDL--GGSWSLWKNIAAQGEE-FGRPEKFCDNLPEESWFVSATLTTLDDRV-APYIEKISKRDPYAGKVVTGGIVTATDSNWMLSYTLNRQPHFKNQPKDQLVVWIYGLLSNKPGDFIKKSITECTGIEIAQEWLYHMGVPVDEIPDIAQN-SCNTIPCYMPYITSYFMPRAMGDRPLVVPEGSANLAFIGNFSETARDTVFTTEYSVRTAMEAVYQLLTIDRGVPEVFASAFDVRTLLASTARLLDGKKLTDI-EAPFILKQIGKLGIHKTKDTIIYDLLKESKLI------------------------

>Lysinibacillus fusiformis

-----------------------------------------MYYSNGNYEAFARPKKPEGVDKKSAYLIGSGLASLSAACFLIRDGQMKGENIHILEELDISGGSLDGILNPTRGFIIRGGREM-EDHFECLWDLFRSIPSLEVDNA-SVLDEFYWLNKEDPNYSKCRLIKDRGQRLEDDGKFTLSDQSSEEMIKLFFTPEEKLEDKKITDVFS--EEFFESNFWLYWSTMFAFEKWHSAMEMRRYIMRFIHHIGGLPDLSALKFTKYNQYESLVLPMIKYLEGHDVDFQYNTVVENVLVDKVGDKKVAHTLVLRKNG-EKKNIELTENELVFVTNGSITESTTYGDNYTPAPV-----NKEL--GGSWSLWKNIAAQDTD-FGRPEKFCDNLPEESWFVSATLTTLDDRV-APYIEKISKRDPYAGKVVTGGIVTATDSNWMLSYTLNRQPHFKHQPKDQLVVWIYGLLSNKPGDFIKKSITECSGIEIAQEWLYHMGVPVDEIPDIAEN-SCNTIPCYMPYITSYFMPRAMGDRPLVVPEGSANLAFIGNFSETVRDTVFTTEYSVRTAMEAVYQLLNIDRGVPEVFASSFDVRTLLASTARLLDGKKLTDI-DAPFILKQIGKLGIHKTKDTIIYDLLKESKLI------------------------

>Lysinibacillus irui

-----------------------------------------MYYSNGNYEAFARPKKPEGVDEKSAYLIGSGLASLSAACFLIRDGQMKGENIHILEELDISGGSLDGTLNPTRGFIIRGGREM-EDHFECLWDLFRSIPSLEVENA-SVLDEFYWLNKEDPNYSKCRLIKDRGQRLQDDGKFTLSDQSSEEMIKLFFTPEEKLEDKKITDVFS--EDFFESNFWLYWSTMFAFEKWHSAMEMRRYIMRFIHHIGGLPDLSALKFTKYNQYESLVLPMIKYLESHDVDFQYNTVVENVLVDKVGDKKVAHTLVLRKDG-VKKNIELTENELVFVTNGSITESTTYGDNNTPAPK-----STDL--GGSWSLWKNIAAQDSE-FGRPEKFCDNLPEESWFVSATLTTLDDRV-APYIEKISKRDPYAGKVVTGGIVTATDSNWMLSYTLNRQPHFKNQPKDQLVVWIYGLLSNKPGNFIKKSITECTGIEIAQEWLYHMGVPLDEIPDLAQN-SCNTIPCYMPYITSYFMPRAMGDRPFVVPKGSANLAFIGNFSETARDTVFTTEYSVRTAMEAVYQLLNIDRGVPEVFASAFDIRTLLASTARLLDGKKLTDI-DAPFILKQIGKLGIHKTKDTIIYDLLKESKLI------------------------

>Lysinibacillus pakistanensis

-----------------------------------------MYYSNGNYEAFARPKKPEGVDEKSAYLVGSGLASLSAACFLIRDGQMKGENIHILEELDISGGSLDGILNPTRGFIIRGGREM-EDHFECLWDLFRSIPSLEIENA-SVLDEFYWLNKEDPNYSKCRLIENRGQRLEDDGKFTLSDKASEEMIKLFFTPEEKLEDVKITDVFS--EEFFESNFWLYWSTMFAFEKWHSAMEMRRYIMRFIHHVGGLPDLSALKFTKYNQYESLVLPMIKYLESHHVDFQFNTVVENVLVDKVGDKKVAHTLVLKQNG-VKKNIELTENELVFVTNGSITESTTYGDNNTPAPV-----STDL--GGSWSLWKNIASQDSE-FGRPEKFCDNLPEESWFVSATLTTLDDRV-APYIEKISKRDPYAGKVVTGGIVTATDSNWMLSYTLNRQPHFKDQPKDQLVVWIYGLLSNKPGDFIKKSITECSGIEIAQEWLYHMGVPVDEIPDLAQN-SCNTIPCYMPYITSYFMPRAMGDRPLVVPKGSANLAFIGNFSETERDTVFTTEYSVRTAMEAVYQLLDIDRGVPEVFASTFDIRTLLSSTARLLDGKKLTDV-DAPFILKQIGKFGIHKTKDTIIYDLLKESKLI------------------------

>Lysinibacillus parviboronicapiens

-----------------------------------------MYYSNGNYEAFARPKKPEGVDEKSAYLIGSGLASLSAACFLIRDGQMKGENIHILEELDISGGSLDGILNPTRGFIIRGGREM-EDHFECLWDLFRSIPSLELENA-SVLDEFYWLNKEDPNYSKCRLIEKRGQRLEDDGKFTLSDKSSEEMIKLFFTPEEKLEDKKITDVFS--DEFFESNFWLYWSTMFAFEKWHSAMEMRRYIVRFIHHIGGLPDLSALKFTKYNQYESLVLPMINYLEKHNVDFQYNTVVENVLVDRVGDKKVAQTLVLRQNG-EKKNIELTENELVFVTNGSITESTTYGDNHTPAPI-----SSDL--GGSWSLWKNIASQDAE-FGRPEKFCDNLPKESWFVSATLTTLDARV-APYIEKISKRDPYAGKVVTGGIVSAKDSNWMLSYTLNRQPHFKNQPKDQLVVWIYGLLSNKPGNFIKKSITECSGIEIAQEWLYHMGVPVDEIPDLAQN-SCNTIPCYMPFITSYFMPRALGDRPLVVPNGSVNLAFIGNFSETARDTVFTTEYSVRTAMEAVYQLLNIDRGVPEVFASAFDIRTLLASTSRLLDGKKLTDI-DAPFILKQIGKLGIHKTKETIIYDLLKESELIE-----------------------

>Lysinibacillus xylanilyticus

-----------------------------------------MYYSNGNYEAFARPKKPVGVDEKSAYLVGSGLASLSAACFLIRDGQMKGENIHILEELDISGGSLDGILNPTRGFIIRGGREM-EDHFECLWDLFRSIPSLEIENA-SVLDEFYWLNKEDPNYSKCRLIENRGQRLEDDGKFTLSDKASEEMIKLFFTPEEKLEDVKITDVFS--EEFFESNFWLYWSTMFAFEKWHSAMEMRRYIMRFIHHVGGLPDLSALKFTKYNQYESLVLPMIKYLESHNVDFQFNTVVENVLVDKVGDKKVAHTLVLKQNG-VKKNIELTENELVFVTNGSITESTTYGDNNTPAPV-----STDL--GGSWSLWKNIASQDSE-FGKPEKFCDNLPEESWFVSATLTTLDDRV-APYIEKISKRDPYAGKVVTGGIVTATDSNWMLSYTLNRQPHFKNQPKDQLVVWIYGLLSNKPGDFIKKSITECSGIEIAQEWLYHMGVPVDEIPDIAQN-SCNTIPCYMPYITSYFMPRAMGDRPLVVPNGSANLAFIGNFSETERDTVFTTEYSVRTAMEAVYQLLDIDRGVPEVFASTFDIRTLLSSTARLLDGKKLTDV-DAPFILKQIGKFGIHKTKDTIIYDLLKESKLI------------------------

>Macrococcoides caseolyticum

-----------------------------------------MYYSNGNYEAFARPKKPEGVDNKSAYLVGSGLASLAAASFLIRDGKMKGENIHILEELDLPGGSLDGILNPERGYIMRGGREM-ENHFECLWDLFRSVPSLEVEDA-SVLDEFYWLNKEDPNYSKCRVIENRGQRLESDGKMTLTKKANKEIIQLCLMKEEQLNDVKISDVFS--KDFLDSNFWIYWKTMFAFEPWHSAMEMRRYLMRFIHHIGGLADFSALKFTKFNQFESLVMPLIEHLKAKNVTFEYGVTVKNIQVECSKESKVAKAIDIVRRG-NEESIPLTENDLVFVTNGSITESTTYGDNDTPAPP-----TTKP--GGAWQLWENLSTQCEE-FGNPAKFYKDLPEKSWFVSATATTNNKEV-IDYIQKICKRDPLSGRTVTGGIVTVDDSNWQLSFTLNRQQQFKNQPDDQVSVWIYALYSDERGERTNKTIVECSGKEICEEWLYHMGVPEEKISALAAE--CNTIPSYMPYITAYFMPRKEGDRPLVVPHGSKNIAFIGNFAETERDTVFTTEYSVRTAMEAVYKLLEVDRGVPEVFASVYDVRILLHALSVLNDGKKLDEI-DMPIYERLVEKRLLKKASGTFIEELLEEANLI------------------------

>Oikeobacillus pervagus

-----------------------------------------MYYSNGNFEAFARPKKPEGVDEKSAYLIGSGLASLSAACFLVRDGQMNGENIHILEELDIAGGSLDGILNPTRGFIIRGGREM-ENHFECLWDLFRSIPSLEVENA-SVLDEFYWLNKEDPNYSKCRLIKNRGERLEDDGKFTLSDQSSEEMIKLFFTPEEKLEDKKITDVFS--EEFFESNFWFYWSTMFAFEKWHSAMEMRRYIMRFIHHIGGLPDLSALKFTKYNQYESLVLPMIEYLKSHDVDFQFNTVVENVLVDQVGDKKVAHTLVLRKDG-VKKNIELTENELVFVTNGSITESTTYGDNNTPAPR-----STDL--GGSWSLWKNIAAQDGE-FGRPEKFCDNLPEESWFVSATLTTLDDRV-APYIEKISKRDPYAGKVVTGGIVTATDSNWMLSYTLNRQPHFKNQPKDQLVVWIYGLLSNKPGNFIKKSITECTGIEIAQEWLYHMGVPVDEIPDLAQN-SCNTIPCYMPYITSYFMPRAMGDRPLVVPKGSANLAFIGNFSETARDTVFTTEYSVRTAMEAVYQLLNIDRGVPEVFASVYDIRTLLASTSRLLDGKKLTDI-DAPFILKQLGKLGVHKTKDTIIYDLLKDSQLI------------------------

>Paenibacillus aceti

-----------------------------------------MYYSNGNYEAFARPKKPQGVDQKSAYLIGSGLAALSAAAFLIRDGQMKGENIHILEELDISGGGLDGIYDDGRGFIIRGGREM-ENHFECLWDLFRSIPSLEVEDA-SVLDEFYWLNKEDPNYSLQRAILNRGEDAHTDGKFTLTQTASMEIIKLFFTPEEALEDMRITDVFS--EDFFNSNFWLYWQTMFAFEPWHSAMEMRRYVARFIHHIGGLPDFSALKFTKYNQYESLALPLMKYLEDHGVTFQYATTVTNVEFEITGNKKVAKKLVYVQKG-QEKQIDLTENDLVFITNGSNTESSTLGDNNTPAVM-----NTSL--GGSWELWKKIAEQDPA-FGRPDKFCGNIEESNW-ESATITTLDNRI-PPYIEKICKRDPFSGRVVTGGIVTVKDSKWLMSFTLNRQPHFKSQPKDQLVVWVYSLYCDVPGDYIKKPMKDCTGIEITEEWLYHMGVPEAEIHDMAVN-SANCVPCMMPYVTSYFMPRAAGDRPNVVPDGCVNAAFIGNFAETPRDTVFTTEYSVRTAMEAVYTLLDIDRGVPEVFGSAYDVRVLMDSTAKMMDGNKLTDM-ELPDTAKLAAMTGVKKISNTRIMQLLKEYKLI------------------------

>Paenibacillus agri

--------------------------------------------------------VREEYRDKQVYFVGGGIASLAGASYLVRDCDFPGQNIHIIEEMKILGGSNDGAGDEENGYVIRGGRMLNDETYENTWELLTSIPSIDRPGL-SVRDEILAFDMANPTHSNARLVNSRGE-VEDVLSMGFDMADRLTLGKLIITPEEKLGKARINDLFG--PHFFTTNFWFMWATTFAFQPWHSAVEFKRYMVRFIHEFPRIQTLEGVTRTPYNQYDSIILPMHKYLEGFGVDFTLKCTVTDLEFK-DGDGITVTRMNVLRQG-VADVIDVKEGDLVIITNGSMTEGSSLGSM-TEAPS-----LNGK--GSSWKLWENIAAKKPG-LGNPSSFADHVDGSKW-ESFTVTFKDSKF-FDLMEKFTRNRAG-----TGALVTFKESSWLMSVVLAFQPHFRNQPEHVRVFWGYGLFPDKVGDFVKKRMCDCTGEEIMEELIGHLHF-EAHREEIMAT--ANCIPCMMPYITSQFMPRLHSDRPQVVPEGSTNLAFIGQYCEIADDVVFTEEYSVRAARIAVYTLLGINRPIEPITQYQYDVRSLFSSFITSFR----------------------------------------------------------------

>Paenibacillus albidus

-------------------------------------------IKGDYSNFWRRMIVNKEYSNRQVYFVGGGIASLAGAAYLVRDCGFPGQNIHIIEEMPILGGSNDGAGNPEQGYVIRGGRMLNDEAYENLWELLSSIPSIDHPGQ-SVREEITAFDDANPTHSNARLVNRDGL-VEDVLSMGFDMTDRLALGKLIVTPEEKMGKARINDWFG--PHFFETNFWYMWATTFAFQPWHSAVEFKRYMLRFFHEFPRIQTLEGVTRTPYNQYDSIILPMQKYLEPFGVDFTLKCTVTDLTFK-EGDDITVTRMHVRRQG-AEDVIEVNEGDLVIVTNGSMTEGASLGSM-NSAPS-----LNGK--GSSWKLWENIAPKKPG-LGNPSSFNDHVDESKW-ESFTVTFHDSTF-FDLMEKFTRNRAG-----TGALVTFKDSSWFMSVVLAFQPHFRNQPEHVKVFWGYGLYPDKVGDFVKKRMSDCTGEEIMEELIGHLHF-EEHKEAIMAT--ANCIPCMMPYITSQFMPRLNSDRPKVVPEGSTNLAFISQFCEIPDDVVFTEEYSVRAARIAVYTLLGINRPIEPINQYQYDVRTLFNSFVTSFR----------------------------------------------------------------

>Paenibacillus albiflavus

---------------------------------------------------MEVTSMKKEHGNRQVYFVGGGLASLAGASYLVRDCDFPGENIHILEGMHILGGSNDGAGDTTNGFVCRGGRMLNEETYENTWELLSSIPSIEQEGV-SVCDEILAFDHAHPTHANARLIDKDGE-VLDVMSMGFDTADRLALGRLMITPEEKLDNLRICDWFAETPHFFETNFWYMWQTTFAFQKWSSLFEFKRYMNRMMFEFSRIQTLEGVTRTPYNQYDSIILPIKTYLDGFGVDFSLKYTVTDLDFE-EGDGITVTAIHYEQEG-KPGIIQLREGDLCIITNGCMTDNATLGDLNTPAKM-----LPEN--PMSGDLWAKIAAKKEG-LGNPTPFFGNAEETNW-ESFTVTCKGNKL-LKMIEKYSRNKPG-----SGALMTFKDSSWLMSIVVAAQPHFKNQPADTTIFWGYGLYTDKVGDYVKKPMRDCTGKEMLIELLHHLHM-EEEMDEIMDS-VVNVIPCMMPYIDAQFQPRAMSDRPQVVPEGSTNLAMISQFVEIPEDMVFTEEYSIRAARIAVYTLMGLNKKICPVTPHRYDVRTLLRALNTSYR----------------------------------------------------------------

>Paenibacillus alvei

-----------------------------------------MYYSNGNYEAFARPKKPLGVDQKSAYLIGSGLASLSAAAFLIRDGQMKGENIHILEELDIAGGGLDGIYDDGRGFIIRGGREM-ENHFECLWDLFRSIPSLETEGA-SVLDEFYWLNKEDPNFSLQRAILNRGEDAHTDGKFTLTQTASMEIIKLFFTPEEALEDMKITDVFS--EDFFNSNFWLYWQTMFAFEPWHSAMEMRRYIARFIHHIGGLPDFSALKFTKYNQYESLALPLMKYLKGHGVTFQYATTVTNVEFDITENKKVANKLVYVQNG-QEKHIDLTENDLVFITNGSNTESSTLGDNNTPAIM-----NTSL--GGSWELWKKIAEQDPA-FGRPDKFCGNIAESNW-ESATITTLDGRI-PPYIEKICKRDPFSGRVVTGGIVTVKDSKWLMSFTLNRQPHFKSQPKDQLVVWVYSLYCDVPGDYIKKPMKDCTGIEITEEWLYHMGVPEAEIHDLAVN-SANCVPCMMPYVTSYFMPRAAGDRPNVVPDDCVNAAFIGNFAETPRDTVFTTEYSVRTAMEAVYTLLDIDRGVPEVFGSAYDVRVLLDSTAKMMDGKKLTDM-ELPPAIQLAAVTGVQKVSHTRIMQLLKEYKLI------------------------

>Paenibacillus amylolyticus

------------------------------------------------------MIVKKEHGNKQVYFVGGGIASLAGAAYLVRDCGFPGEHIHIIEEMPILGGSNDGAGNPDQGYIIRGGRMLNDEAYENLWELLASIPSIDRPGI-SVRQEITEFDDANPTHSNARLINRDGK-VEDVLSMGFDMADRLAMGKLIITPEDTLGKLRINDWFG--PHFFKTNFWYMWATTFAFQPWHSAVEFKRYMLRFFHEFPRIQTLEGVTRTPFNQYDSIILPLHNYLEPFGVDFTLKCTVTDLDFK-DGDGITVSRMHVLREG-EEEVIDILEGDLVIVTNGSMTEGADIGSM-TYAPK-----LNGK--GSSWKLWENIAAKKPL-LGNPSSFNDHVDESKW-ESFTVTFQDSVF-FDLMEKFTRNRAG-----TGALVTFKDSSWFMSVVLAFQPHFRGQPEHVNVFWGYGLSPDNVGDYVKKRMCDCTGEEIMQELIGHLHF-QEHQEDIMAT--ANCIPCMMPYITAQFMPRLNSDRPKVVPEGSTNLAFISQFCEIPDDVVFTEEYSVRAARIAVYTLLGENRPIEPINKYQYDVRSLFSSFVTSFR----------------------------------------------------------------

>Paenibacillus anaericanus

---------------------------------------------------MEANMMEQNKDNRQVYFVGGGLASLAGAAYLVRDCGFSGKNIHVLEGMNILGGSNDGAGSPVQGFVCRGGRMLNEETYENFWELFNSIPSIEQPDI-SVTDEILAFDHEHPTHANARLINKDGE-VLDVMSMGFNTADRLAIGKLMIAPEEKLDNLRICDWFAHTPHFFETNFWYMWQTTFAFQKWSSLFEFRRYMNRMMFEFSRIQTLEGVTRTRYNQYESVILPLKAYLDKFDVDFGLKCVVTDLDFD-ENEGITVTAIHYTQDGITDNLIQLHEGDLCIVTNGCMTDNATLGDLHTAAPL-----IADK--PMSGELWAKIAAKKEG-LGNPAPFFGRPEETNW-ESFTVTMQGNKL-LKMIEKYSRNVPG-----SGALMTFKDSSWLMSIVVAAQPHFKDQPADQTIFWGYGLYTDKVGDYVKKPMRDCTGEEMLIELLHHLHF-EDQLDEILSD-VINVIPCMMPYIDAQFQPRKMTDRPAVVPAGSTNLAMISQFVEIPEDMVFTEEYSVRAARIAIYTLLGLDKKICPVTPHQYDVRTLFKAFNTTYR----------------------------------------------------------------

>Paenibacillus antibioticophila

-----------------------------------------MYYSNGNYEAFARPEKPKNVERKSAYLVGSGLASLAAACFLVRDGQMKGEHIHVLEEMSLPGGACDGIKDAQKGFIIRGGREM-ENHFECLWDLFRSIPSIETEGL-SVLDEFYRLNKHDPNYSLMRASVNRGEDAHTDGKFTLSEKASMQIVKLFMTRDEDLYDITIDDVFD--EEFYASNFWLYWQTMFAFEKWHSALEMKLYLQRFIHHIGGLPDFSALKFTKYNQYESLILPMVKFLEAHGVHFQYNTRVTNVIFDIRDGKKTAKQLICIHNG-KEETIDLIEDDLVFVTNGSCTENSTLGDDDHAPVM-----NTQPGEGGCWQLWKNIAAQDPS-FGRPEKFCTNIPATNW-ESATVTTLDHRI-PRYIEKMSKRDPFSGKVVTGGIITVKDSSWLMSYTINRQPHFKEQPKDQLVVWIYGLYTDAPGDFVKKPMKECTGREIVEEWLYHMGVPEAEILELATT-GAHCIPCMMPYITAFFMPRTEGDRPKVVPEGSVNFAFIGQFADTVRDTVFTTEYSVRTAMEAVYTLLDVDRGVPEVFASCYDVRVLLDSTAKMLDGKKLTDL-KLPFITKLVEKQLLKKASGTIIGELLERYKLI------------------------

>Paenibacillus apiarius

-----------------------------------------MYYSNGNYEAFARPKKPLGVDQKSAYLIGSGLASLSAAAFLIRDGQMKGENIHILEELDIAGGGLDGIYDDGRGFIIRGGREM-ENHFECLWDLFRSIPSLETEGA-SVLDEFYWLNKEDPNFSLQRAILNRGEDAHTDGKFTLTQTASMEIIKLFFTPEEALEDMKITDVFS--EDFFNSNFWLYWQTMFAFEPWHSAMEMRRYIARFIHHIGGLPDFSALKFTKYNQYESLALPLMKYLKGHGVTFQYATTVTNVEFDITENKKVAKKLVYVQNG-QEKHIDLTENDLVFITNGSNTESSTLGDNNTPAIM-----NTSL--GGSWELWKKIAEQDPA-FGRPDKFCGNIAESNW-ESATITTLDDRI-PPYIEKICKRDPFSGKVVTGGIVTVKDSKWLMSFTLNRQPHFKSQPKDQLVVWVYSLYCDVPGDYIKKPMKDCTGIEITKEWLYHIGVPEAEIHDLAVN-SANCVPCMMPYVTSYFMPRAAGDRPNVVPDGCVNAAFIGNFAETPRDTVFTTEYSVRTAMEAVYTLLDIDRGVPEVFGSAYDVRVLMDSTAKMMDGKKLTDM-ELPPTIQLAAMTGVQKISHTRIMQLLKEYKLI------------------------

>Paenibacillus apii

--------------------------------------------------------VVQEYENKQVYFVGGGIASLAGAAFLIRDCDFPGSGIHIIEEMNILGGSNDGAGSEEQGYVIRGGRMLNDETYENLWDLLMSIPSLDHPGK-SVREEIIAFDNANPTHSNARLVNASGE-VVDVLSMGFDMADRLALGKLIITPEEQMGKARISDWFG--PHFFTTNFWYMWATTFAFQPWHSAVELKRYMIRFMHEFPRIQTLEGVTRTPYNQYDSIILPMKKYLEDHGVDFTLKCTVTDLDFK-EGDGITVTGLHVIRGG-AEEYIAVKEEDLVIITNGSMTESSSLGSM-TSAPR-----LNEK--GSSWKLWERIAVKKPG-LGTPSSFDDHIDGSKW-ESFTVTFSDTVF-FDLMEQFSRNRPG-----TGALVTFKDSSWLMSIVLAYQPHFRNQPEHVRVFWGYGLYPDKEGDFVKKKMSDCTGGEIMTELLGHLHL-EEHKEAIMST--ANCIPCMMPFITAQFMPRAIGDRPKVVPDGSTNLAFIGQFCEIPDDVVFTEEYSVRTARIAVYTLLGVNKPITPINHYQYDVRTLIASLVTSFR----------------------------------------------------------------

>Paenibacillus aquistagni

-----------------------------------------MYYSNGNYEAFARPKKPLGVDHKSAYLIGSGLASLSAAAFLVRDGQMKGENIHIFEELDIAGGGLDGIYDDGRGFIIRGGREM-ENHFECLWDLFRSIPSLEVEDA-SVLDEFYWLNKEDPNYSLQRAILNRGEDAHTDGKFTLSQTASMEIIKLFFTPEEALEDMKITDVFS--EDFFNSNFWLYWQTMFAFEPWHSAMEMRRYIARFIHHIGGLPDFSALKFTKYNQYESLALPLMHYLKDHGVSFQYATTVTNVEFEITELKKVAKKLVYVQKG-QQKHIDLTENDLVFITNGSNTESSTLGDNNKPAVM-----NPAL--GGSWELWKKIAEQDPA-FGRPDKFCGNIAESNW-ESATITTLDDRI-PPYIEKICKRDPFSGKVVTGGIVTVKDSKWLMSFTLNRQPHFKSQPNDQLVVWVYSLYCDVPGDYIKKPMKDCTGIEITQEWLYHIGVPEAQIHELAAN-AANCVPCMMPYVTSYFMPRAAGDRPNVVPDNCVNAAFIGNFAETPRDTVFTTEYSVRTAMEAVYTLLDIDRGVPEVFGSAYDVRVLLDSTAKMMDGKKLTEM-ELPPAVQLAAMTGVQKISHTRVMQLLKEYKLI------------------------

>Paenibacillus auburnensis

--------------------------------------------------------VKKEYGNQQVYFVGGGIASLAGAVYLVRDCDFPGQNIHIIEEMKILGGSNDGAGDVEHGYVIRGGRMLNDEAYENLWELLNTIPSIDHPGQ-SVREEIIAFDTANPTHSNARLVDSKGE-VQDVMSMGFDMADRLAMGKLIITPEEAMGKARINDWFG--PHFFKTNFWYMWATTFAFQPWHSAVEFKRYMLRFMHEFPRIQTLEGVTRTPYNQYDSIILPLHKYLEPFGVDFTLKCTVTDLQFK-DGDGITVTGMNVSRQG-VPDIIEVQEGDLVIVTNGSMTEGSSLGSM-TSAPR-----LNGK--GSSWKLWENIAAKKPG-LGNPSAFDDHVDESKW-ESFTVTFQDSDF-FDLMEKFTRNRAG-----TGALVTFKDSSWFMSVVLAFQPHFRGQPEHVKVFWGYGLYPDNVGDFVKKRMCDCTGEEIMQELIGHLHF-EAHKDEIMDT--ANCIPCMMPYITSQFMPRLNSDRPKVVPEGSTNLAFISQFCEIPDDVVFTEEYSVRAARIAVYTLMGVNRPVEPINQYQYDVRTLLSGLVTSFR----------------------------------------------------------------

>Paenibacillus azoreducens

-----------------------------------------MYYSNGNYEAFARPKKPHGVDQKSAYLIGAGLASLSAAAFLIRDGQMKGENIHILEELDISGGGLDGIYDDGRGFIIRGGREM-ENHFECLWDLFRSIPSLETEGA-SVLDEFYWLNKEDPNYSLQRAILNRGEDAHTDGKFTLSQSASMEIIKLFFTPEEALEDMKITDVFS--EDFFNSNFWLYWQTMFAFEPWHSAMEMRRYIARFIHHIGGLPDFSALKFTKYNQYESLALPLLEYLKSHGVTFQYATTVTNVEFDITENKKVAKKLVYVQKG-QEKHIDLTENDLVFITNGSNTECSSLGDNNTPAVM-----NTSL--GGSWELWKKIAEQDPA-FGRPDKFCGNIAESNW-ESATITTLDGRI-PPYIEKICKRDPFSGKVVTGGIVTVKDSKWLMSFTLNRQPHFKSQPKDQLVVWVYSLYCDVPGDYIKKPMKDCTGIEITEEWLYHMGVPEAEIHELAVN-SANCVPCMMPYVTSYFMPRAAGDRPNVVPDGCVNAAFIGNFAETPRDTVFTTEYSVRTAMEAVYTLLDVDRGVPEVFGSAYDVRVLMDSTAKMMDGKKLTEM-ELPPTVQLAAMTGLEKISRTRIMQLLKEYKLI------------------------

>Paenibacillus barcinonensis

--------------------------------------------------------VKKEHDSKQVYFVGGGIASLAGAAYLVRDCDFPGENIHIIEEMPILGGSNDGAGNPEQGYVIRGGRMLNDEAYENLWELLGTIPSIDHPGM-SVREEITAFDNANPTHSNARLINRDGQ-VEDVLSMGFDMADRLAMGKLIITPEDTLGKLRINDWFA--PHFFQTNFWYMWATTFAFQPWHSAVEFKRYMLRFFHEFPRIQTLEGVTRTPYNQYDSIILPLHRYLEPFGVDFTLKCTVTDLDFK-EGDGITVTRMHVRRNG-ADEVIEIREGDLVIVTNGSMTEGADLGSM-TCAPK-----LNGK--GSSWHLWENIAAKKPR-LGNPSSFNEHVDESKW-ESFTVTFQDSVF-FDLMEKFTRNRAG-----TGALVTFKDSSWFMSVVLAFQPHFRNQPEHVKVFWGYGLYPDNVGDYVKKRMCDCTGEEIMQELIGHLHF-QEHQESIMAT--ANCIPCMMPYITAQFMPRLNSDRPQVVPEGSTNLAFISQFCEIPDDVVFTEEYSVRAARIAIYTLLGINRPVEPIHQYQYDVRTLFSSFVTSFR----------------------------------------------------------------

>Paenibacillus barengoltzii

--------------------------------------------------------MSADRHTHHVYFVGGGIASLAGAAFLIRDCDFPGQNIHVLEEMKVLGGSNDGAGDSERGYVIRGGRMLNDETYENTWDLLRSIPSIDHPGL-SVRDEIIAFDTAHQTHSNARLVNRDGQ-VVDVTSMGFDMTDRMAMVKLIMAPEESLGTARINDWFG--PHFFKTNFWYMWATTFAFQPWHSAVEFKRYMLRFMHEFPRIHTLEGVTRTPYNQYDSLILPLQKYLEKHGVDFSLKCTVTDLDFR-EGDEITVTRIHYLKDG-EPQVLDLGEDDLVIVTNGSMTEGYSLGSM-STAPK-----LNGK--GSSWQLWERIAAKKPG-LGRPSVFADHIEESKW-ESFTVTCQDSRF-FDRMEAFSRNKAG-----TGALVTFKDSSWFMSIVLAHQPHFRNQPEHVKVFWGYGLFPDRAGDYVPKKMSECTGEEILTELLHHLHF-EQDMEAIIST--ANCIPCMMPFITSQFMPRAIGDRPKVIPDGSINLAFIGQFCEIPDDVVFTEEYSVRAARMAVYGLLGLNKPIAPINQYQYDVRTLLQSLVTSFR----------------------------------------------------------------

>Paenibacillus borealis

------------------------------MIPSITGLYYTRVRYSNQANLWRRQSVKKEYGNEQVYFVGGGIASLAGAAYLVRDCGFPGQNIHIIEEMKILGGSNDGAGSEEHGYVIRGGRMLNDETYENLWELLNTIPSIDQPGL-TLREEITQFDDANPTRSKARLVDSKGE-IQDVNSMGFDMADRLALGKLIITPEAKMGKARINDWFG--PHFFTTNFWYMWATTFAFQPWHSAVELKRYMIRFIHEFPRIQTLEGVTRTPYNQYDSIILPLHQYLEPFGVDFTLKCTVTDLEFK-EGDGITVTKMKVVRQG-VEDVIEINEGDRVIITNGSMTEGSSLGSM-TSAPR-----LNGK--GSSWKLWENIAAKKPG-LGNPSSFDDHVDESKW-ESFTVTFQDSKF-FDLMEDFTRNRAG-----TGALVTFKDSSWLMSVVLAFQPHFRNQPEHVKVFWGYGLYPDKVGDYVHKKMCDCTGEEIMQELIGHLHF-EAHKEEIMAT--ANCIPCMMPYITSQFMPRLNSDRPQVVPEGSTNLAFVGQYCEIPDDVVFTEEYSVRAARIAVYTLFGINRPVEPIKEYQHDVRTLLSSLVTSFR----------------------------------------------------------------

>Paenibacillus bouchesdurhonensis

----------------------------------------------------------MGAYSNQVYFIGGGIASLAGAAFLIRDCGLPGQQIHIIEDLKVLGGSNDGAGDQEHGYVIRGGRMLNDETYENLWDLLSSIPSVDDPSQ-SVREEIVAFDTAHPTHSRARLVNKNGE-IEDVASMGFDMGDRIAMAKLIIAPEAQLGRLRINEWFG--PHFFQTNFWFMWATTFAFQPWHSAVELKRYMIRFMHEFPRIHTLEGVTRTPYNQYDSLIVPLHQYLQEHGVDFEMKCTVTDLDFK-EGDGITVTRMHYVKDG-KKKVLDLAEEDLVIMTNGSMTDSSSLGSM-TTPPR-----LNGK--GSSWQLWDRIAAKKPGRLGNPSSFDDHIDESKW-ESFTVTCEGSRF-FELMESFTRNKAG-----TGALVTFKDSNWFMSIVLAHQPHFRNQPDDVKVFWGYGLYPDQVGDYVKKRMCDCTGEEILTELLQHLKF-ENDKDEIIRS--ANCIPCMMPYITAQFMPRTLGDRPQVIPEGSTNLAFIGQFCEIPDDVVFTEEYSVRSARIAVYQLLGIDKPIAPINQYQYDVRTLLQSLATSFR----------------------------------------------------------------

>Paenibacillus bovis

-----------------------------------------MRYTNGNYEAFVRSRKPEGVDSKSAYIIGGGLAGLAAAAFLIRDGYMKGEHVHILEELAVSGGSLDGTLMPHDGFVTRGGREM-EAHFECLWDLFRSIPSLEEENA-SVLDEFYWLNYDDPNFSNCRIIHQRGERTPDDGQFTLSKTAQKELISLFMTSEDQLENKRIEDVFG--EDFFASNFWLYWCSMFAFEKWHSAIEMRRYVMRFVHHIEGLPDFTALKFTRYNQYESLIKPLLSYLHNHNVDFQYNTQVNNVIVDIAGETKTARKLLLTRSG-QAEEVALSENDLVFVTNGSITESSTQGNHHTPAPI-----TRDL--GGSWNLWKNISKQSPE-FGNPAVFCENLPDESWYVSATITWENEDI-APYLEQITKRKLHTGKIVSGGIVTIKDSNWLMSFATHRQPHFKEQQDHQTITWVYGLLSNIPGNYVQKPIENCTGEEIVQELLYHLGVPEEDIPRIAGQ-SCHSVPVYMPFITSYFMLRKADDRPLVVPNGSQNLAFIGNFAETERDTVFTTEYSVRTAMEAVYQLLKVERGVPEVFASAYDLRTLAKAVYYLADQTPLAEM-ELPFIERKMMEGLVHKTKNTYVSDVLKDAHLI------------------------

>Paenibacillus camerounensis

--------------------------------------------------------VTKEYGNRQVYFVGGGIASLAGAAYLVRDCNFAGSDIHIIEEMHILGGSNDGIGDVDQGYVIRGGRMLNDEVYENLWELLSTIPSIDHPGQ-SVREEIIAFDTANPTHSNARLIDRSGQ-VQDVLSMGFDMADRLAMGKLIITPEEAMGKARINDWFG--PHFFETNFWYMWATTFAFQPWHSAVEFKRYMLRFMHEFPRIQTLEGVTRTPYNQYDSIILPLQKYLEPLGVDFSLKCTVTDLQFK-DGDGITVTQMNVLREG-VADVIEVKEDDLVIVTNGSMTEGSSLGSM-TAAPE-----LNGK--GSSWKLWENIAAKKPG-LGNPSPFADCVDESKW-ESFTVTFQDSVF-FDLMEQFTRNRAG-----TGALVTFKDSSWFMSVVLAFQPHFRNQPEHVKVFWGYGLYPDKVGDFVNKRMCDCTGEEIMQELIGHLHF-EAHRDEIMAT--ANCIPCMMPYITSQFMPRLGTDRPQVVPAGSTNLAFISQFCEIPDDVVFTEEYSVRAARTAVYTLLGMGRPVEPIRQYHYDVRTLFASFVTSFR----------------------------------------------------------------

>Paenibacillus dokdonensis

----------------------------------------------------MLNQEKCNAHERKAYFIGGGIASLAGAAFLIRDGGMEGRNIHILEDLGINGGALDGIGSGNQDYVIRGGRMLNEPTYECTWNLLADIPSIDRPGK-SVKDEIHDFTAQYPTHAKARLIDRTRQ-VVDVKHMGFSYEDRMDMSRLLIKSEEDLGAKRINEWFS--EHFFSTNFWYMWATMFAFQPWHSAVEFRRYMIRFMHEFHRIDTLAGVARTPYNQYDSIVLPIQKWLEKQGVQYSLNTTVTDIDFAGEGSKRTAERIHLRQAN-HTGEILVSPEDLVFFTNGSMTENSDLGGM-DRAPV-----LKDK--GPSFGLWDKLSAKQPG-FGNPAAFSNNITQSKW-ESFTVTCRNPLF-FRRMEEFTGNKTG-----TGALVTFKDSNWLMSVVLAHQPHFRNQPDDIQVFWGYSLAGDQKGNYVDKTMAECTGAEIPTELCGHFRF-TDDLPLLLET--SDVIPCMMPFITSQFMPRVKGDRPDVVPEGSTNFALLGQYTEIPEDVVFTVEYSVRSAMTAVYKLLNIEKDIPQPYKGQHSPKVLFESLVTALR----------------------------------------------------------------

>Paenibacillus donghaensis

--------------------------------------------------------VNKEISDQQVYFVGGGLASLAGAVYLIRDCGFTGPQIHIIEEMPILGGSNDGAGSGKEGYVIRGGRMLNDEVYENLWELLKTIPSIDHPEL-SVREEIIAFDTANPTHGNARLIDRNGE-VQDVLSMGFDTADRLAMGKLIITPEDKMGKARINDWFG--PHFFTTNFWYMWATTFAFQPWHSAVEFKRYMLRFMHEFPRIQTLEGVTRTPYNQYDSIILPLHRYLEPHGVDFTLKCTVTDLQFK-EGEGITVTALNVLRQG-KESVIEVQEKDLVIVTNGSMTEGSSLGSM-TSAPK-----LGGK--GSSWKLWENIAAKKPG-LGNPSSFDDHVDGSKW-ESFTVTFQDSKF-FDLMEKFTRNRAG-----TGALVTFKDSSWFMSVVLAFQPHFRNQPENVTVFWGYGLYTDNVGDYVKKRMCDCTGEEIMEELIGHLHF-EEHREEIMAT--ANCIPCMMPYITSQFMPRLGSDRPQVVPAGSTNLAFVGQYCEIPDDVVFTEEYSVRAARIAVYTLLGSNRPIEPINQYQYDVRTLFSSFVTSYR----------------------------------------------------------------

>Paenibacillus durus

--------------------------------------------------------VIQEYENKQVYFVGGGIASLAGAAFLIRDCGFPGSGIHIIEEMNILGGSNDGAGSEEHGYVIRGGRMLNDETYENLWDLLMSIPSLDHPGK-SVREEIIAFDNANPTHSNARLVNASGE-VVDVLSMGFDMADRLAMGKLIITPEEQMGKARISDWFG--PHFFTTNFWYMWATTFAFQPWHSAVELKRYMIRFMHEFPRIQTLEGVTRTPYNQYDSIILPMKKYLEDHGVDFTLKCTVTDLDFK-EGDGITVTGLHVIRGG-AEEYIAVKEEDLVIITNGSMTESSSLGSM-TSAPR-----LNEK--GSSWKLWERIAAKKPG-LGNPSSFDDHIDGSKW-ESFTVTFSDTVF-FDLMEQFSRNRPG-----TGALVTFKDSSWLMSIVLAYQPHFRNQPEHVQVFWGYGLYPDKEGDFVKKKMSDCTGEEIMTELLGHLHF-EEHKEAIMAT--ANCIPCMMPFITAQFMPRAIGDRPKVVPDGSTNLAFIGQFCEIPDDVVFTEEYSVRTARIAVYTLLGVNKPIAPINHYQYDVRTLFASLVTSFR----------------------------------------------------------------

>Paenibacillus etheri

--------------------------------------------------------VKKEYGNKQVYFVGGGIASLAGAAYLVRDCDFPGQNIHIIEEMKILGGSNDGAGDAEHGYVIRGGRMLNDETYENTWDLLMSIPSLDHPEK-SVREEIIEFDTANPTHGNARLVNRNGE-VEDVLSMGFDMADRLAMGKLIITPEEKMGKARINDWFG--PHFFTTNFWYMWATTFAFQPWHSAVELKRYMIRFVHEFPRIQTLEGVTRTPYNQYDSIILPMHKYLEGFGVDFTLKCTVTDLQFK-DGDGITVTQMNVLRQG-APDVINISEDDIVIVTNGSMTEGSSLGSM-TSAPR-----LNGK--GSSWKLWENIAAKKPG-LGNPSSFDDHVDGSKW-ESFTVTFQDSKF-FDLMEKFTRNRAG-----TGALVTFKDSNWLMSVVLAFQPHFRNQPEHVKVFWGYGLYPDNVGDFVKKKMSECTGEEIMEELIGHLHF-EEHKDEIMAT--ANCIPCMMPYITSQFMPRLNSDRPKVVPEGSTNLAFVGQYCEIPDDVVFTEEYSIRAARTAVYTLLGINRPIEPINQHQYDVRTLFTSFITSFR----------------------------------------------------------------

>Paenibacillus faecis

--------------------------------------------------------MAENGNGRQVYFVGGGIASLAGAAFLVRDCGFPGEQIHVLEEMKILGGSNDGAGNEQQGYVIRGGRMLNDETYENLWDLLSSIPSLDQPGL-SVREEIVAFDTAHPTHSNARLVNKEGQ-VEDVTSMGFDMADRLAMAKLIVTPEEKLGTARINDWFG--PHFFETNFWFMWATTFAFQPWHSAVELKRYMIRFMHEYPRIHTLEGVTRTPYNQYDSIILPLHKYLEEHGVDFTLKCTVTDLDFK-EGDGITVTRLHYMKDG-AEQVLDLGEGDLVIVTNGSMTESSSLGSM-TSAPE-----LLGK--GSSWKLWERIAAKKPG-LGNPSPFADHIEGSKW-ESFTVTCQDSRF-FDLMEAFSRNKAG-----TGALVTFKDSSWFMSIVLAHQPHFRNQPEHVKVFWGYGLFPDRVGDYVKKKMSDCTGEEILTELLHHLHF-EKDRDAIIST--ANCIPCMMPFITAQFMPRALGDRPKVVPDGSTNLAFISQFCEIPDDVVFTEEYSVRAARIAVYTLLGLDKPIAPINQYQYDVRTLLQGFVTSFR----------------------------------------------------------------

>Paenibacillus fonticola

---------------------------------------------------------MMGTYSNQVYFVGGGIASLAGAAFLIRDCGFPGQQIHIIEELKVLGGSNDGAGDREQGYVIRGGRMLNDETYENLWDLLSSIPSLDNPDQ-SVRDEIMAFDTAHPTHSRARLVNKDGE-VEDVASMGFDMDDRIAMAKLIITPEAQLGRLRINEWFG--AHFFKTNFWYMWATTFAFQPWHSTVELKRYMIRFMHEFPRIHTLEGVTRTPYNQYDSLIVPLHRYLQEHGVDFEMKCTVTDLDFK-AGDGITVTSMHYVKDG-VEKALDLAEEDLVIVTNGSMTESSSLGSM-TAPPQ-----LNGK--GCSWQLWDRIAAKKPGLLGNPSSFDDHIDESKW-ESFTVTCEDSRF-FDLMEKFSRNKAG-----SGALVTFKDSSWFMSIVLAHQPHFRNQPEDVKVFWGYGLYPDQVGDYVKKRMCDCTGEKILTELLHHLKF-ENELDDIIRT--ANCIPCMMPYITAQFMPRTLGDRPQVVPEGSTNLAFIGQFCEIPDDVVFTEEYSVRSARTAVYQLLGVDKPIAPINQYQYDVRTLLQSVATSFR----------------------------------------------------------------

>Paenibacillus glacialis

--------------------------------------------------------MKKEYENRQVYFIGGGLGSLAGASYLVRDCNFPGENIHILEGMHILGGSNDGAGDTTNGFVCRGGRMLNEETYENTWELLSSIPSIEQEGM-SVCDEILAFDHAHPTHANARLINKDGE-VLDVMSMGFNTADRLALGRLMITPEEKLDNLRICDWFAETPHFFETNFWYMWQTTFAFQKWSSLFEFKRYMNRMIFEFPRIQTLEGVTRTPYNQYDSIILPIKTYLDGFGVDFSLKYTVTDLDFE-EGDGITVTAIHYEQDG-KSGMIQLHNGDLCIMTNGCMTDNATLGDLNTPAKM-----VSEN--PMSGNLWAKIALKKEG-LGNPAPFFGNAEETNW-ASFTVTCKGDKL-LKMIEKYSRNKPG-----SGALMTFKDSNWLMSIVVAAQPHFKNQPADTTIFWGYGLYTDREGDYVKKPMRDCTGKEMLIELLHHLHM-EEDMDEIMDS-VVNVIPCMMPYIDAQFQPRAMSDRPKVVPDGSTNLAMISQFVEIPEDMVFTEEYSIRAARIAVYTLLGLNKKVCPVTPHMYDVRTLMKALNTSYR----------------------------------------------------------------

>Paenibacillus graminis

--------------------------------------------------------VKKEYGNSQVYFVGGGIASLAGAAYLVRDCDFPGQNIHIIEEMKILGGSNDGAGSGEQGYVIRGGRMLNDEAYENLWELLSTIPSIDHPGQ-SVREEITAFDDANPTHANARLINKNGE-VEDVLSMGFDMADRLAMGKLIITPEDKMGKARINDWFA--PHFFQTNFWYMWATTFAFQPWHSAVEFKRYMLRFIHEFPRIQTLEGVTRTPYNQYDSIILPLHKYLEPFGVDFTLKCTVTDLDFK-DGDGITVTKMHVLRQG-VPDVIEIAEGDRVIVTNGSMTEGSSLGSM-TTAPR-----LNGK--GSSWKLWENIAAKKPG-LGNPSSFDDHVDGSKW-ESFTVTFQDSAF-FDLMEKFTRNRAG-----TGALVTFKDSSWFMSVVLAFQPHFRNQPEHVKVFWGYGLYPDKVGDFVKKRMCDCTGEEIMQELIGHLHF-EAHKEEIMAT--ANCIPCMMPYITAQFMPRLNSDRPKVVPEGSTNLAFISQFCEIPDDVVFTEEYSVRAARIAVYTLMGVNRPVEPIKQYQYDVRTLFSSFVTSFR----------------------------------------------------------------

>Paenibacillus guangzhouensis

--------------------------------------------------------MKKDHGNRQVYFVGGGLASLAGATYLIRDCDFPGENIHILEGMHILGGSNDGAGDKMQGFVCRGGRMLNEETYENTWELLSSIPSIEQEGV-SVCDEILAFDHAHPTHANARLINKDGE-VLDVMSMGFNTADRLALGRLMITPEEKLDNLRICDWFAETPHFFETNFWYMWQTTFAFQKWSSLFEFRRYMNRMMFEFSRIQTLEGVTRTPYNQYDSIILPIKTYLDGYGVDFSLKYTVTDLDFA-EGDGITVTAIHYEQDG-KSGVIQLQKDDLCIVTNGCMTDNATLGDLNTPAKM-----VPEN--PMSGNLWAKIAAKKEG-LGNPEPFFGKVEETNW-ESFTVTCKGNKL-LKMIEKYSRNKPG-----SGALMTFKDSNWLMSIVVAAQPHFKNQPADTTIFWGYGLYTDQEGNYVKKPMRDCTGKEMLIELIHHLHM-EEDMDEIMDS-VVNVIPCMMPYIVSQFQPRAMSDRPKVVPEGSTNLGLISQFVEIPEDMVFTEEYSIRAARIAVYTLMGLNKKVCPVTPHMYDVRTLLKALNTSYR----------------------------------------------------------------

>Paenibacillus helianthi

---------------------------------------------MGTYQRI-NPLVQEGIASKKAYLVGGGIGSLSAAAFLIRDGHMPGRNIHILEQSAVYGGSMDGAGNAKDGYSARGGREI-EEHFECFMELFGFIPSLTNPDR-TVLEEFRELNLAEPIESHCRLVEKAGQ-KADFSSLGLSTAHALQLGKLVMVTEEKLGAVTIEQFFD--PSFLDTNFWYFWRSMFAFENWHSVVEVKRYMERFMHLISGMNQLKGILHTEYNQFDSLILPLMKWLESEGVHFDKGHQVTDLDVDFTGGEKTVTAIHVRVNG-TPKTISTSRSDLVMVTNGSMTENSTLGDMDHPAVL-----NRSVTERGCWSLWEKLAAKSPD-FGHPEVFCGDIDKTKW-LSFTMTFTDDEIVFPYLLELTGDLPG-----MGGVVTIKDSNWMMSWTAPKQPHFINQPENVKVLWAYGLFPDAEGNYIKKKMSDCTGRELLEELCYHIGL-KDRIPEILEH-TTNVIPCMMPYITSQFMPRVAGDRPQVVPQGSTNLAFLGQFAEVPDDCVFTVEYSVRSAMMAVYSLLALEKEVIPVHPSKFDVRVLLTAVRTCLGNKPLP-----------LDRTLGQLLSGTVISKLL------------------------------

>Paenibacillus jilunlii

--------------------------------------------------------VKKEYGNSQVYFVGGGIASLAGAAYLVRDCDFPGQNIHIIEEMKILGGSNDGAGSGEQGYVIRGGRMLNDEAYENLWELLSTIPSIDHPGQ-SVREEITAFDDANPTHANARLINKNGE-VEDVLSMGFDMADRLAMGKLIITPEDKMGKARINDWFA--PHFFQTNFWYMWATTFAFQPWHSAVEFKRYMLRFMHEFPRIQTLEGVTRTPYNQYDSIILPLHKYLEPFGVDFTLKCTVTDLDFK-DGDGITVTKMHVLRQG-VPDVIEIAEGDRVIVTNGSMTEGSSLGSM-TTAPR-----LNGK--GSSWKLWENIAAKKPG-LGNPSSFDDHVDGSKW-ESFTVTFQDSVF-FDLMEKFTRNRAG-----TGALVTFKDSSWFMSVVLAFQPHFRNQPEHVKVFWGYGLYPDKVGDFVKKRMCDCTGEEIMQELIGHLHF-EAHKEEIMAT--ANCIPCMMPYITAQFMPRLNSDRPKVVPEGSTNLAFISQFCEIPDDVVFTEEYSVRAARIAVYTLMGVNRPVEPINQYQYDVRTLFSSFVTSFR----------------------------------------------------------------

>Paenibacillus lentus

--------------------------------------------------------MDMGTYSNQVYFIGGGIASLAGAAFLIRDCGFPGQQIHIIEELKVLGGSNDGAGDRKHGYVIRGGRMLNDETYENLWDLLSSIPSVDDPSQ-SVREEIVAFDTAHPTHSRARLVNKNGE-IEDVSSMGFDMGDRMAMAKLILAPETQIGRMRINEWFG--PHFFQTNFWYMWATTFAFQPWHSAVELKRYMLRFMHEFPRIHTLEGVTRTPYNQYDSLIVPLQQYLQKHSVDFELKCTVTDLDFK-EGDDITVTRMHYVKDG-KQKILDLAEEDLVIITNGSMTESSDLGSM-TTPPR-----LNGK--GSSWQLWDRIAAKKPGRLGNPSSFDDHIDESKW-ESFTVTCKESRF-FDLMESFTRNKAG-----TGALVTFKDSSWFMSIVLAHQPHFRNQPEDIKVFWGYGLYPDQVGDYVKKRMCDCTGEEILTELLHHLKF-EDDKDEIIRS--ANCIPCMMPYITAQFMPRTLGDRPEVVPEGSTNLAFIGQFCEIPDDVVFTEEYSVRTARIAVYQLLGIDKPIVPINQYQYDVRTLLQSLATSFR----------------------------------------------------------------

>Paenibacillus motobuensis

-----------------------------------------MYYSNGNYEAFARPKKPHGVDQKSAYLIGSGLASLSAAAFLIRDGQMKGENIHILEELDISGGGLDGIYDDGRGFIIRGGREM-ENHFECLWDLFRSIPSLETEGA-SVLDEFYWLNKEDPNYSLQRAILNRGEDAHTDGKFTLTQTASMEIIKLFFTPEEALEDLKITDVFS--EDFFNSNFWLYWQTMFAFEPWHSAMEMRRYVARFIHHIGGLPDFSALKFTKYNQYESLALPLMKYLEDQGVTFQYATTVTNVEFEITGTKKVAKKLVYVQKG-QEKHIELTENDLVFITNGSNTESSTLGDNHTPAVM-----NTSL--GGSWELWKKIAEQDPA-FGRPDKFCGNIAESNW-ESATVTTLDDRI-PPYIEKICKRDPFSGKVVTGGIVTVKDSKWLMSFTLNRQPHFKSQPKDQLVVWVYSLYCDVPGDYIKKPMKDCTGIEITEEWLYHMGVPEAEIHDMAVN-SANCVPCMMPYVTSYFMPRAAGDRPNVVPDGCVNAAFIGNFAETPRDTVFTTEYSVRTAMEAVYTLLDIDRGVPEVFGSAYDVRVLLDSTAKMMDGNKLTDM-ELPDTVKLAAMTGVKKISNTRIMQLLKEYKLI------------------------

>Paenibacillus macquariensis

--------------------------------------------------------MKKEYENKQVYFIGGGLGSLAGASYLVRDCNFPGENIHILEGMHILGGSNDGAGNTTNGFVCRGGRMLNEETYENTWELLSSIPSIEQEGV-SVCDEILAFDHTHPTHANARLINKDGE-VLDVMSMGFNTADRLALGRLMITPEEKLDNLRISDWFAETPHFFETNFWYMWQTTFAFQKWSSLFEFKRYMSRMIFEFPRIQTLEGVTRTPYNQYDSIILPIKTYLDGFGVDFSLKYKVTDLDFE-EGDGITVTAIHYEQDG-KLGMIQLQEGDLCIMTNGCMTDNATLGDLNTPAKI-----VSEN--PMSGNLWAKIAAKKEG-LGNPAPFFSNAEETNW-ASFTVTCKGDKL-LKMIEKYSRNKPG-----SGALMTFKDSNWLMSIVVAAQPHFKNQPADTTILWGYGLYTDKEGDYVKKPMRDCTGKEMLVELLHHLHM-EEDMDEVMDS-VVNVIPCMMPYIDAQFQPRAMSDRPKVVPDGSTNLAMISQFVEIPEDMVFTEEYSIRAARIAVYTLLGLNKKVCPVTPHMYDVRTLMKALNTSYR----------------------------------------------------------------

>Paenibacillus mellifer

--------------------------------------------------------MSAELNTRQVYLVGGGIASLAGAAFLVRDCDFPGQNIHVLEEMKVLGGSNDGAGNGEQGYVIRGGRMLNDETYENTWDLLRSIPSLDHPGL-SVRDEIIAFDTAHPTHSNARLVNREGQ-VEDVTSMGFDMADRLAMVKLIMTPEEKMGKARINDWFG--PHFFETNFWYMWATTFAFQPWHSAVEFKRYMLRFMHEFPRIHTLEGVTRTPYNQYDSIILPLQKYLEEHGVDFSLKCTVTDLDFR-EGDEITVTRMHYLKDG-EPRVLDLAKDDLVIVTNGSMTEGYSLGSM-TTAPK-----LNGK--GSSWQLWDRIAAKKPG-LGHPSSFDDHIDGSKW-ESFTVTCQDSRF-FDRMEAFSRNKAG-----TGALVTFKDSSWLMSIVLAHQPHFRNQPERVKVFWGYGLFPDRVGDYVPKKMSECTGEEILTELLHHLHF-EQDMEAIIST--ANCIPCMMPFITAQFMPRTIDDRPKVIPDGSTNLALISQFCEIPDDVVFTEEYSVRAARMAVYGLLGLNKPIAPINQYQYDVRTLLQGLVTSFR----------------------------------------------------------------

>Paenibacillus monticola

--------------------------------------------------------VKKEYGNQQVYFVGGGIASLAGAAYLVRDCDFPGQNIHIIEEMKILGGSNDGSGNGEQGYVIRGGRMLNDEAYENLWELLQSIPSIDHPEK-SVREEIIEFDNANPTHANARLVNRKGE-IEDVLSMGFDMADRLALGKLIITPEDKMGKARINDWFG--PHFFQTNFWYMWATTFAFQPWHSAVEFKRYIIRFMHEFPRIQTLEGVTRTPYNQYDSIILPMHKYLEPHGIDFTLKCTVTDLQFK-DGDGITVTKMNVLRQG-VADVIEVNEGDLVIVTNGSMTEGSSIGSM-TKAPL-----LNGK--GSSWKLWENIASKKPG-LGNPSSFDDHVDESKW-ESFTVTFQDSKF-FDRMEKFSRNRAG-----TGALVTFKDSSWFMSIVLAFQPHFRNQPENVTVFWGYGLYPDKVGDFVKKRMSDCTGEEIMEELIGHLHF-EESKEEIMAS--ANCIPCMMPYITSQFMPRLNSDRPKVVPEGSTNLAFISQYCEIPDDVVFTEEYSVRAARTAVYTLLGMNRPIEPINQYQYDVRTLFTSFVTSFR----------------------------------------------------------------

>Paenibacillus odorifer

--------------------------------------------------------VKKEYGNRQVYFVGGGIASLAGAAYLVRDCDFPGQNIHIIEEMKILGGSNDGAGDAEHGYVVRGGRMLNDEAYENLWELLTTIPSVDRPGL-SVRDEIIAFDTANPTHSNARLVNKDGK-VEDVLSMGFDMADRLAMGKLIITPEDKMGKARINDWFA--PHFFTTNFWYMWATTFAFQPWHSAVEFKRYMLRFFHEFPRIQTLEGVTRTPYNQYDSIILPLHKYLEPFGVDFTLKCTVTDLQFK-KGDGITVTQMNVVREG-VKDVIDVKEDDLVIITNGSMTEGSSLGSM-TSAPR-----LNGK--GSSWKLWENIASKKPS-LGNPSSFDDHVDGSKW-ESFTVTFHDSKF-FDLMEKFTRNRAG-----TGALVTFKDSSWFMSVVLAYQPHFRNQPEHVKVFWGYGLYPDKVGDFVKKRMCDCTGEEIMQELIGHLHF-EEHKDEIMAT--ANCIPCMMPYITSQFMPRLNSDRPKVVPEGSTNLAFIGQFCEIPDDVVFTEEYSVRAARIAVYTLLGIERPIEPINQYQYDVRTLFSSFVTSFR----------------------------------------------------------------

>Paenibacillus oralis

---------------------------------------------------MEATVMSADYSGRQVYFVGGGIASLAGAAFLIRDCGFPGQQIHILEEMKVLGGSNDGAGNVEQGYVIRGGRMLNDETYENTWDLLWSIPSLDNPDL-SLREEIIAFDTAHPTHSNARLVNKHGE-VVDVSSMGFDMADRLAMVKLIMTPEEALGKARINDWFG--PHFFETNFWYMWATTFAFQPWHSAVELKRYMIRFMHEFPRIHTLEGVTRTPYNQYDSIILPMHKYLEEHGVDFTLKCTVTDLDFK-DGDGITVTRMHYVKDG-AETALDLTEGDLVIVTNGSMTEGSSLGSM-TTAPK-----LGGK--GSSWKLWENIAAKKAG-LGNPSSFADHIDGSKW-ESFTVTFRDSRF-FELMEAFSRNKPG-----TGALVTFKDSSWLMSIVLAYQPHFRNQPEHVKVFWGYGLFPDRIGDYVNKKMSDCTGEEILTELLHHLKF-EKDMDEIIAS--ANCIPCMMPYITAQFMPRAIGDRPKVIPDGSTNLAFISQFCEISDDVVFTEEYSVRAARMAVYGLLGINKPIAPINQYQYDVRTLLQGLVTSFR----------------------------------------------------------------

>Paenibacillus phocaensis

--------------------------------------------------------MSAEHNTRQVYFVGGGIASLAGAAFLIRDCDFPGRNIHVLEEMKVLGGSNDGAGDGKQGYVIRGGRMLNDETYENTWDLLRSIPSIDQPGL-SVRDEIIAFDTAHPTHSNARLVNREGQ-VEDVTSMGFDMADRLAMMKLIMTPEEKMGKMRINDWFG--AHFFETNFWYMWATTFAFQPWHSAVELKRYMIRFMHEFPRIHTLEGVTRTPYNQYDSIILPLQKYLEKYGVDFSLKCTVTDLDFK-EGDGITVSRMHYIKDG-VAGVLDLAEGDLVIITNGSMTEGYSLGSM-TTAPK-----LNGK--GSSWQLWDRIAAKKTG-LGNPSSFDDHIDGSKW-ESFTVTCQDSRF-FDLMEAFSRNKAG-----TGALVTFKDSSWFMSIVLAHQPHFRNQPEQVKVFWGYGLFPDRIGDYVPKKMSECTGEEILTELLHHLHF-EQDMEAILST--ANCIPCMMPFITAQFMPRAIDDRPKVIPDGSTNLAFISQFCEIPDDVVFTEEYSVRAARMAVYGLLGLDKPIAPINQYQYDVRTLLQGLVTSFR----------------------------------------------------------------

>Paenibacillus phytohabitans

---------------------------------------------MGTYQRI-HPLVQEGIASRKAYLVGGGIGSLSAAAFLIRDGHMPGRNIHILEQSAVYGGSMDGAGNAKDGYSARGGREI-EEHFECFMELFGFIPSLTNPDR-TVLDEFRELNLAEPIESHCRLVEKEGQ-PADFSTLGLSTAHALQLGKLTLATEERLGAVTIEQFFD--PTFLETNFWYFWRSMFAFENWHSVVEVKRYMERFMHLISGMNQLKGILHTEYNQFDSLILPLMKWLESQGVNFDKGHQVTDLELDFTGGEKTVTAIQLLVNG-EPKTLSTARGDLVMVTNGSMTENSTLGDLDHPAIL-----NRSVTERGCWSLWEKLAAKSPD-FGHPEVFCGDIDKSKW-LSFTMTFTDDDIVFPYLLELTGDDPG-----MGGVVTIKDSSWMMSWTAPKQPHFINQPENVKVLWAYGLFPDAEGDYIKKKMSDCSGRELLEELMYHIGL-KNRIPEILEH-TTNVIPCMMPYITSQFMPRVAGDRPQVVPQGSTNLAFLGQFAEVPDDCVFTVEYSVRSAMMAVYNLLALEKEVIPVHPSKYDVRVLLTALRTCLGNKPLP-----------LDKTLGQLLAGTVISKLL------------------------------

>Paenibacillus pini

------------------------------------------------------MAQASKNSEKQAYFIGGGIASLAGAAFLIRDGEMKGHNIHILEDLNINGGALDGAKSEQDGFVMRGGRMLNKPTYECTWNLLADIPSVDTPGI-SVKDEIHAFTDQYPTHAQARLVDKDRQ-IVDVKHMGFSNQDRLHMSKLLIESEEDLGAKRINEWFT--AHFFQTNFWYMWATMFAFQPWHSAVEFKRYMIRFMHEFHQIDTLAGVARTPFNQYDSIVVPIQQWLEQHGVQYSLNTTVTDIDFVNNGTERTAERIHFNRDG-LNGQIHVSPNDLVFFTNGSMTEHSDLGSM-HSAPT-----LRDK--GPSFGLWEKIAAKQPG-FGNPSAFDDHIAESKW-ESFTVTCNTPLF-FERMEEFSGNEAG-----TGALVTFKDSNWLMSIVLAHQPHFRNQPDNVQVFWGYALSGDQQGNYVNKPMSECTGAEILTELCGHLRF-TEELPHLLET--SNVIPCMMPFITSQFMPRIQGDRPQVIPEGSTNFAFLGQYTEIPEDVVFTVEYSVRSAMTAVYHLLNIDKEIPKPYKGQHHVKVLFESLVTAIR----------------------------------------------------------------

>Paenibacillus piscarius

---------------------------------------------MGTYQRI-HPRVQEGIASRKAYLVGGGIGSLSAAAFLIRDGHMPGRNIHILEQSAVYGGSMDGAGNAKDGYSARGGREI-EEHFECFMELFGFIPSLTNPDR-TVLDEFRELNLAEPIESHCRLVEKQGT-PADFSTLGLTTAHSLQLGKLALATEERLGAVTIEQFFD--PSFLETNFWYFWRSMFAFENWHSVVEVKRYMERFMHLISGMNQLKGILHTEYNQFDSLILPLMKWLEGEGVHFDKSHQVTDLTLELAGGEQAVTAIELLVNG-TPKRIAVGRGDLVMVTNGSMTENSTLGDLDHAAVL-----NRSVTERGCWSLWEKLAVKSPD-FGRPEVFCGDIDKSKW-LSFTITLTDDEIVFPHLLELTGDAPG-----MGGVVTIKDSSWMMSWTAPKQPHFINQPENVKVLWAYGLFPDAAGDYIHKKMSDCTGRELLEELCYHMGL-ADRIPEILEH-TVNVIPCMMPYITAQFMPRVAGDRPQVVPKGSVNLAFLGQFAEVPDDCVFTVEYSIRSAMMAVYKLLQLEKEVIPVHPSKYDVRVLLTAVRTCLGNKPLP-----------PDKTLGELLAGTVISKLI------------------------------

>Paenibacillus polysaccharolyticus

--------------------------------------------------------VKKEHGSKQVYFVGGGIASLAGAAYLVRDCDFPGENIHIIEEMPILGGSNDGAGNPEQGYVIRGGRMLNDEAYENLWELLATIPSIERPDM-SVREEITAFDNANPTHSNARLINRDGQ-VEDVLSMGFDMADRLAMGKLIITPEDTLGKLRINDWFA--PHFFHTNFWYMWATTFAFQPWHSAVEFKRYMLRFFHEFPRIQTLEGVTRTPYNQYDSIILPLHRYLEPYGVDFTLKCTVTDLDFK-EGDGITVTGMHVRRNG-AEEVIDILEGDLVIVTNGSMTEGADLGSM-TSAPK-----LNGK--GSSWKLWENIAAKKPM-LGNPSSFDDHVDESKW-ESFTVTFQDSVF-FDLMEKFTRNRAG-----TGALVTFKDSSWFMSVVLAFQPHFRDQPDHVKVFWGYGLYPDNVGDFVKKRMCDCTGEEIMQELIGHLHF-QEHQEEIMAT--ANCIPCMMPYITAQFMPRLNSDRPQVVPKGSTNLAFISQFCEIPDDVVFTEEYSVRAARIAVYTLLGINRPVEPIHQYQYDVRTLFSSFVTSFR----------------------------------------------------------------

>Paenibacillus profundus

--------------------------------------------------------MNKEYNNKQVYFVGGGIASLAGAAFLVRDCDFPGQNIHIIEETKILGGSNDGSGNGEQGYVIRGGRMLNDETYENTWDLLKSIPSIDDPAK-SVRDEIIEFDSAHPTHANARLVNKNAE-VQDVTSMGFDMVDRIAMSKLIITPEEKMGKARIQDWFG--PHFFETNFWYMWATTFAFQPWHSVVELKRYMIRFMHEFPRIQTLEGVTRTPFNQYDSLTLPLHKYLAQYGVDFDMKCTVTDLDFK-DSSDITVTKIHYVKEG-TAGTIDLNEGDLVIVTNGSMTEGYSLGSM-TKAPS-----LNGK--GSSWKLWDRLAAKKSG-LGNPSSFDDNIDGSKW-ESFTVTCQDSRF-FDLMEAFSRNKAG-----TGALVTFKESSWLMSIVLAHQPHFRNQPEHVKVFWGYGLFPDNTGDYVKKKMSDCTGEEILTELLHHLKF-DKDMDAIIQS--ANCIPCMMPYITAQFMPRAVGDRPQVVPQGSTNLAFVGQFCEIPDDVVFTEEYSVRAARIAVYTLLGLNKPIAPINQYQYDVRTLLNGLVTSFR----------------------------------------------------------------

>Paenibacillus pseudetheri

--------------------------------------------------------VKKEYGNKQVYFVGGGIASLAGAAYLVRDCDFPGQNIHIIEEMKILGGSNDGAGDAEHGYVIRGGRMLNDETYENTWDLLMSIPSLDHPEK-SVREEIIEFDTSNPTHANARLVNQSGE-VEDVLSMGFDMADRLAMGKLIITPEEKMGKARINDWFG--PHFFTTNFWYMWATTFAFQPWHSAVELKRYMIRFVHEFPRIQTLEGVTRTPYNQYDSIILPMHKYLEGFGVDFTLKCTVTDLQFK-DGDGITVTQMNVLRQG-APDVINISEDDIVIVTNGSMTEGSSLGSM-TSAPR-----LNGK--GSSWKLWENIAAKKTG-LGNPSSFDDHVDGSKW-ESFTVTFQDSKF-FDLMEKFTRNRAG-----TGALVTFKDSSWLMSVVLAFQPHFRNQPEHVKVFWGYGLYPDNVGDFVKKKMSECTGEEIMEELIGHLHF-EAHKDEIMAT--ANCIPCMMPYITSQFMPRLNSDRPKVVPEGSTNLAFVGQYCEIPDDVVFTEEYSIRAARTAVYTLLGINRPIEPINQHQYDVRTLFTSFITSFR----------------------------------------------------------------

>Paenibacillus rhizolycopersici

--------------------------------------------------------MSAERNTRQVYLVGGGIASLAGAAFLVRDCDFPGQNIHVLEEMKVLGGSNDGAGNGEQGYVIRGGRMLNDETYENTWDLLRSIPSIDHPGL-SVRDEIITFDNAHPTHSNARLVNREGQ-VEDVTSMGFDMADRLAMAKLILTPEEKMGKAQINDWFG--PHFFETNFWYMWATTFAFQPWHSAVELKRYMLRFMHEFPRIHTLEGVTRTPYNQYDSIILPLQQYLEEHGVDFSLKCTVTDLDFR-EGDGITVTRMHYLKDG-VPGVLDLTEGDLVIVTNGSMTEGYSLGSM-TSAPK-----LNGK--GSSWQLWERMAAKKPG-LGHPSSFDDHIDGSKW-ESFTVTCQDSRF-FDRMEAFSRNKAG-----SGALVTFKESSWLMSIVLAHQPHFRNQPDQVKVFWGYGLFPDRVGDYVPKKMSECTGEEILTELLHHLHF-DQDMEAIIST--ANCIPCMMPFITAQFMPRAIDDRPKVIPDGSTNLALISQFCEIPDDVVFTEEYSVRAARMAVYGLLGLNKPIAPINQYQYDVRTLLQGLITSFR----------------------------------------------------------------

>Paenibacillus rhizophilus

---------------------------------------------------MEAIVVKEEDKNKQVYFVGGGIASLAGAAFLVRDCDFPGIGIHIIEEMNILGGSNDGAGSEEHGYVIRGGRMLNDETYENTWDLLMSIPSLDHPGK-SVREEIIAFDDANPTHSNARLVNGNGE-VVDVMSMGFNMADRLAMGKLIITPEEKMGKARISDWFG--PHFFTTNFWYMWATTFAFQPWHSAVELKRYMIRFMHEFPRIQTLEGVTRTPYNQYDSIILPMKKYLEDHGVDFTLKCTVTDLDFK-EGDGITVTCMHVVRCG-AEELIAINEEDLVIITNGSMTEGSSLGSM-TSAPR-----LKEK--GSSWKLWDRIAAKRPG-FGNPSSFDDHIEESKW-ESFTVTFSDTVF-FDLMEQFSRNRAG-----TGALVTFKDSSWLMSVVLAYQPHFRNQPEHIRVFWGYGLYPDKEGDFVKKKMSDCTGEEIMTELLGHLHF-DEHKEAIMAT--ANCIPCMMPFITAQFMPRAARDRPKVVPDGSTNLAFIGQFCEIPDDVVFTEEYSVRAARIAVYTLLGVNKPIAPINKYQYDVRTLFTSFVTSFR----------------------------------------------------------------

>Paenibacillus riograndensis

---------------------------------------------------MEATRVKKEYGNSQVYFVGGGIASLAGAAYLVRDCDFPGQNIHIIEEMKILGGSNDGAGNSEQGYVIRGGRMLNDEAYENLWELLSTIPSIDHPGQ-SVREEITAFDDANPTHANARLINKNGE-VEDVLSMGFDMADRLAMGKLIITPEDKMGKARINDWFA--PHFFQTNFWYMWATTFAFQPWHSAVEFKRYMLRFMHEFPRIQTLEGVTRTPYNQYDSIILPLHKYLEPFGVDFTLKCTVTDLDFK-DGDGITVTKMHVLRQG-VPDVIEIAEGDRVIVTNGSMTEGSSLGSM-TTAPR-----LNGK--GSSWKLWENIAAKKPG-LGNPSSFDDHVDGSKW-ESFTVTFQDSVF-FDLMEKFTRNRAG-----TGALVTFKDSSWFMSVVLAFQPHFRNQPEHVKVFWGYGLYPDKVGDFVKKRMCDCTGEEIMQELIGHLHF-EAHKKEIMAT--ANCIPCMMPYITAQFMPRLNSDRPKVVPEGSTNLAFISQFCEIPDDVVFTEEYSVRAARIAVYTLMGVNRPVEPINQYQYDVRTLFSSFVTSFR----------------------------------------------------------------

>Paenibacillus rubinfantis

---------------------------------------------------MEATRVKKEYGNSQVYFVGGGIASLAGAAYLVRDCDFPGQNIHIIEEMKILGGSNDGAGNSEQGYVIRGGRMLNDEAYENLWELLSTIPSIDHPGQ-SVREEITAFDDANPTHANARLINKNGE-VEDVLSMGFDMADRLAMGKLIITPEDKMGKARINDWFA--PHFFQTNFWYMWATTFAFQPWHSAVEFKRYMLRFMHEFPRIQTLEGVTRTPYNQYDSIILPLHKYLEPFGVDFTLKCTVTDLDFK-DGDGITVTKMHVLRQG-VPDVIEIAEGDRVIVTNGSMTEGSSLGSM-TTAPR-----LNGK--GSSWKLWENIAAKKPG-LGNPSSFDDHVDGSKW-ESFTVTFQDSVF-FDLMEKFTRNRAG-----TGALVTFKDSSWFMSVVLAFQPHFRNQPEHVKVFWGYGLYPDKVGDFVKKRMCDCTGEEIMQELIGHLHF-EAHKKEIMAT--ANCIPCMMPYITAQFMPRLNSDRPKVVPEGSTNLAFISQFCEIPDDVVFTEEYSVRAARIAVYTLMGVNRPVEPINQYQYDVRTLFSSFVTSFR----------------------------------------------------------------

>Paenibacillus sabinae

--------------------------------------------------------VIQEYENKQVYFVGGGIASLAGAAFLIRDCGFPGSGIHIIEEMSILGGSNDGAGSEEQGYVIRGGRMLNDETYENLWDLLMSIPSLDHPGK-SVREEIIAFDNANPTHSNTRLVNASGE-VADVLSMGFDMADRLAMGKLIITPEEQMGKARISDWFG--PHFFTTNFWYMWATTFAFQPWHSAVELKRYMIRFMHEFPRIQTLEGVTRTPYNQYDSIILPIKKYLEDHGVDFTLRCTVSDLDFK-EGDDITVTGLHVVRDG-AEEHIAVKEEDLVIITNGSMTESSSLGSM-TSAPR-----LNEK--GSSWKLWERIAAKKPG-FGNPSSFDDHIDGSKW-ESFTVTFGDTVF-FDLMEQFSRNRPG-----TGALVTFKDSSWLMSIVLAYQPHFRNQPEHVRVFWGYGLYPDKEGDFVKKKMSDCTGEEIMTELLGHLHF-EAHKEAIMAT--ANCIPCMMPFITAQFMPRAIGDRPKVVPDGSTNLAFIGQFCEIPEDVVFTEEYSVRAARIAVYTLLGVNKPITPINHYQYDVRTLLASLVTSFR----------------------------------------------------------------

>Paenibacillus segetis

--------------------------------------------------------MEQNKDNRQVYFVGGGLASLAGAAYLIRDCGFSGKNIHVLEGMNILGGSNDGAGSPIQGFVCRGGRMLNEETYENFWELFNSIPSIEQPDI-SVTDEILAFDHAHPTHANARLINKDGE-VLDVMSMGFNTADRLALGKLMIAPEEKLDNLRICDWFAHTPHFFETNFWYMWQTTFAFQKWSSLFEFRRYMNRMMFEFSRIQTLEGVTRTRYNQYESVILPLKAYLDKFDVDFGLKCTVTDLDFD-ENEGITVTAIHYTQDGTKDNIIQLHEGDLCIVTNGCMTDNATLGDLHTAAPL-----IADK--PMSGELWAKIAAKKEG-LGNPTPFFGRPEETNW-ESFTVTLQGNKL-LKMIEKYSRNVPG-----SGALMTFKDSSWLMSIVVAAQPHFKDQPADQTIFWGYGLYTDKVGDYVKKPMRDCTGEEMLIELLHHLHF-EDQLDEILSD-VINVIPCMMPYIDAQFQPRKMTDRPAVVPAGSTNLAMISQFVEIPEDMVFTEEYSVRAARIAVYTLLGLDKKVCPVTPHQYDVRTLFKALNTTYR----------------------------------------------------------------

>Paenibacillus selenitireducens

-------------------------------------MCSILGSRTTINEHMEVTSMKKEYGNRQVYFVGGGLASLAGASYLIRDCDFPGENIHILEGMHILGGSNDGAGDKMQGFVCRGGRMLNEETYENTWELLASIPSIEQEGV-SVCDEILAFDHEHPTHANARLINKDGE-VLDVMSMGFNTADRLALGRLMITPEEKLDNLRICDWFAETPHFFETNFWYMWQTTFAFQKWSSLFEFRRYMNRMMFEFSRIQTLEGVTRTPYNQYDSIILPIKTYLDGYGVDFSLKYTVTDLDFE-EGDGITVTAIHYEQDG-KSGMIQLRNGDLCIVTNGCMTDNATLGDLNTPAKM-----LPED--PMSGNLWAKIAAKKEG-LGNPTPFFGNAEETNW-ESFTVTCKGDKL-LKMIEKYSRNKPG-----SGALMTFKDSNWLMSIVVAAQPHFKNQPADTTIFWGYGLYTDKEGDYVKKPMRDCTGKEMLVELIHHLHM-EEDMDEIMDS-VVNVIPCMMPYIDAQFQPRAMSDRPKVVPDGSTNLAMISQFVEIPEDMVFTEEYSIRAARIAVYTLLGLNKKVCPVTPHMYDVRTLLKALNTSYR----------------------------------------------------------------

>Paenibacillus senegalimassiliensis

--------------------------------------------------------MSTDYSGKQVYFVGGGLASLAGAAFLVRDCDFPGEQIHVLEEMHVLGGSNDGAGDERQGYVIRGGRMLNDEAYENTWDLLSSIPSLDHPGQ-SVRDEIVAFDTAHPTHSNARLVNKHGQ-VEDVTSMGFDTADRLAMAKLVMTPEEKLGKARIHDWFG--PHFFETNFWYMWATTFAFQPWHSAVELKRYMLRFMHEFPRIQTLEGVTRTPYNQYDSIIVPLYRYLEDHGVDFSLKCTVTDLDFK-EGDGITVTRMHMIQDG-TPATLDIAEGDLVLITNGSMTESSSLGSM-TTPPQ-----LLGK--GSSWELWERIAAKQPG-VGQPATFADHIDESKW-ESFTVTCQDSRF-FDLMEAFSRNKAG-----SGALVTFKDSSWLMSIVLAHQPHFRNQPEHVKVFWGYGLFPDKVGDYVPKRMSDCSGEEILTELLHHLHF-EKDLDAILST--ANCIPCMMPYITAQFLPRLIDDRPKVIPDGSTNLAFISQFCEIPDDVVFTEEYSIRAARMAVYGLLDVQKPIAPIHQYQYDVRILLQGLVTSFR----------------------------------------------------------------

>Paenibacillus shirakamiensis

------------------------------------------------------MTNTKENEHRKAYLIGGGIASLSAAVFMIRDGHVKGKNITILEERRVNGGSLDGSGTEEMGYVIRGGRMLNEPTYECTWSMLDCIPSLDRPGI-SVKQEIQQFSSEHPTHSKARVVDASRH-IVDVEHMQFSNQDRLDIGKLLVASEESLGTERIKDWFE--PEFFETNFWYMWATMFAFQPWHSLVEFKRYMIRFMHEFPRINTLEGVARTPYNQYDSIVLPVQTWLQEQGVRYDNNIRVTDIVFRAGIAEKTVERIIYERDG-VEASMEVAAEDLVFFTNGSMTEASDLGTM-QQAPK-----LGQK--GSSFRLWEKLASTQTD-FGNPSSFADHIEGSKW-ESFTVTCTDPLF-FDFMESFTDNVAG-----SGGLVTFKDSNWLMSIVLAHQPHFRNQPEHIQVFWGYGLFPDRVGNYVDKKMSDCTGEEVLQELMGHLKFNAEQVQKMLST--SNCIPCMMPFITSQFMPRAIGDRPQVVPQGSTNFAFLGQYTELPEDVVFTVEYSVRSAQTAVYQLLHLDMEIPAVYHGRHHVKVLWDSLTTMIR----------------------------------------------------------------

>Paenibacillus silagei

--------------------------------------------------------VKKEYGNEQVYFVGGGIASLAGAAYLVRDCGFPGQNIHIIEEMKILGGSNDGAGDAEHGYVIRGGRMLNDETYENLWELLSTIPSIDQPGL-TLREEIIQFDESNPTRGQARLINSKGE-VLDVTSMGFDMADRLAMGKLIMTPEPAMGTLRINDWFG--PHFFTTNFWYMWATTFAFQPWHSAVEFKRYMLRFMHEFPRIQTLEGVTRTPYNQYDSIILPLHQYLEPLGVDFTLKCTVTDLEFK-DGDGITVTAMKVVRNG-VEDTIQVHEGDRVIVTNGSMTEGSSLGSM-TSAPG-----MNGK--GSSWKLWETIAAKKPG-LGNPSSFDDHVDESKW-ESFTVTFKDSKF-FDLMEKFTRNRAG-----TGALVTFKDSSWFMSVVLAFQPHFRNQPEHVRVFWGYGLYPDKVGDYVHKKMCNCTGEEIMKELIGHLHF-EAHKEEIMAT--ANCIPCMMPYITSQFMPRLNSDRPQVVPEGSTNLAFIGQYCEIPDDIVFTEEYSVRAARIAVYTLFGLNRPVEPINAYQNDVRTLFSSLVTSFR----------------------------------------------------------------

>Paenibacillus silvae

--------------------------------------------------------VKKEHGSKQVYFVGGGIASLAGAAYLVRDCDFPGENIHIIEEMPVLGGSNDGAGNAEQGYVIRGGRMLNDEAYENLWELLATIPSIDHPGM-SVREEITAFDNANPTHSNARLINRDGQ-VEDVLSMGFDMADRLAMGKLIITPEDTLGKLRINDWFA--PHFFQTNFWYMWATTFAFQPWHSAVEFKRYMLRFFHEFPRIQTLEGVTRTPYNQYDSIILPLHQYLEPFGVDFTLKCTVTDLDFK-EGDRITVTRMYVRRNG-AEEVIDIHEGDLVIVTNGSMTEGADLGSM-TSAPK-----LNGK--GSSWKLWENIAAKKPL-LGNPSSFNDHVDESKW-ESFTVTFQDSVF-FDLMEKFTRNRAG-----TGALVTFKDSSWFMSVVLAFQPHFRNQPEHVNVFWGYGLYPDNVGDYVKKRMCDCTGEEIMQELIGHLHF-QEHQEAIMAT--ANCIPCMMPYITAQFMPRLNSDRPQVVPEGSTNLAFISQFCEIPDDVVFTEEYSVRAARIAVYTLLGINRPVEPIHQYQYDVRTLFSSFVTSFR----------------------------------------------------------------

>Paenibacillus sinensis

--------------------------------------------------------MRQEYEGRQVYLVGGGIASLAAAAFLVRDCDFPGSGIHVIEELHILGGSNDGAGSEEHGYVIRGGRMLNDETYENLWDLLSSIPSLNHPEI-SVREEITSFDNANPTHSRARLVNAAGE-IVNSSSMGFDMADRLAMGKLILTPEDKMGTARINDWFA--PHFFETNFWYMWATTFAFQPWHSAVELKRYMIRFMHEFPRIHTLEGVTRTPYNQYDSVILPLKKYLEKQSVDFSLKCTVTDLDFK-EGEGITVTGMHVLKGD-VKDRIAVGDRDLVIVTNGSMTESSSLGSM-TAPPV-----LNGK--GSSWELWDRLAAKKPA-LGNPSPFDDHIDGSKW-ESFTVTFSDSVF-FDLMEKFSGNRAG-----TGALVTFKDSSWLMSIVLAYQPHFRNQPEHVRVFWGYGLNVDKEGDFVKKKMSECTGEEIMTELIGHLHF-EQHRDAIMAT--ANCIPCMMPFITSQFMPRALGDRPKVVPDGSTNLAMVGQFCEIPEDVVFTEEYSVRAARIAVYKLLGINKPVAPVHHYQYDVRTLVSSLATSLH----------------------------------------------------------------

>Paenibacillus sonchi

---------------------------------------------------MEATRVKKEYGNSQVYFVGGGIASLAGAAYLVRDCDFPGQNIHIIEEMKILGGSNDGAGNVEQGYVIRGGRMLNDEAYENLWELLSTIPSIDHPGQ-SVREEITAFDNANPTHGNARLINKNGE-VEDVLSMGFDMADRLAMGKLIITPEDKMGKARINDWFA--PHFFQTNFWYMWATTFAFQPWHSAVEFKRYMLRFMHEFPRIQTLEGVTRTPYNQYDSIILPLHKYLEPFGVDFNLKCTVTDLDFK-DGDGITVTKMHVLRQG-VPDVIKIAEGDRVIVTNGSMTEGSSLGSM-TTAPR-----LNGK--GSSWKLWENIAAKKPG-LGNPSSFDDHVDGSKW-ESFTVTFQDSVF-FDLMEKFTRNRAG-----TGALVTFKDSSWFMSVVLAFQPHFRNQPEHVKVFWGYGLYPDKVGDFVKKRMCDCTGEEIMQELIGHLHF-EAHKKEIMAT--ANCIPCMMPYITAQFMPRLNSDRPKVVPEGSTNLAFISQFCEIPDDVVFTEEYSVRAARIAVYTLMGVNRPVEPINQYQYDVRTLFSSFVTSFR----------------------------------------------------------------

>Paenibacillus sophorae

--------------------------------------------------------VKQEYENKQVYFVGGGIASLAGAAFLVRDCDFPGSGIHIIEEMNILGGSNDGAGSEEHGYVIRGGRMLNDETYENTWDLLMSIPSLDHPGQ-SVREEIIAFDNANPTHSNARLVNASGE-VVDVLSMGFDMADRLSMGKLIITPEEQMGKARISDWFG--PHFFTTNFWYMWATTFAFQPWHSAVELKRYMIRFMHEFPRIQTLEGVTRTPYNQYDSIILPMKKYLEDHGVDFTLKCTVTDLEFK-EDSGITVTGLHVVQGG-AEELIEVKEEDLVIITNGSMTESSSLGSM-TSAPR-----LNEK--GSSWKLWERIAAKRPG-LGNPSSFDDHIEGSKW-ESFTVTFSDTVF-FDLMEQFSRNRAG-----TGALVTFKDSSWLMSIVLAYQPHFRNQPEHVRVFWGYGLYPDKEGDFVKKKMSDCTGEEIMTELLGHLHF-EEHKEAIMAT--ANCIPCMMPFITAQFMPRAIGDRPKVVPDGSTNLAFIGQFCEIPDDVVFTEEYSVRTARIAVYTLLGLNKPITPINHYQYDVRTLLASLVTSFR----------------------------------------------------------------

>Paenibacillus stellifer

--------------------------------------------------------MRQDYEGRQVYLVGGGIASLAAAAFLVRDCDFPGSGIHVIEELHILGGSNDGAGSEEHGYVIRGGRMLNDETYENLWDLLASIPSLDHPEI-SVREEITAFDNANPTHSRARLVNAAGE-IINSSSMGFDMADRLAMGKLILTPEDKMGTAHINDWFA--PHFFETNFWYMWATTFAFQPWHSAVELKRYMIRFMHEFPRIHTLEGVTRTPYNQYDSVILPLKKYLEKQSVDFSLKCTVTDLDFK-EGEGITVTGIHVLKGD-VKDRIAVGDRDLVIVTNGSMTESSSLGSM-TAPPV-----LNGK--GSSWELWDRLAAKKPG-LGNPSPFDDHIDGSKW-ESFTVTFSDSVF-FDLMEKFSGNRAG-----TGALVTFKDSSWLMSIVLAYQPHFRNQPEHVRVFWGYGLNVDKEGDFVKKKMSECTGEEIMTELIGHLHF-GQHRDTIMAT--ANCIPCMMPFITSQFMPRALGDRPKVVPDGSTNLAMVGQFCEIPEDVVFTEEYSVRAARIAVYKLLGINKPVAPVHHYQYDVRTLVSSLATSLH----------------------------------------------------------------

>Paenibacillus taiwanensis

-----------------------------------------MYYSNGNYEAFARPKKPLGVDHKSAYLIGSGLASLSAAAFLIRDGQMKGENIHILEELDIAGGGLDGIYDDGRGFIIRGGREM-ENHFECLWDLFRSIPSLETEGA-SVLDEFYWLNKEDPNFSLQRAILNRGEDAHTDGKFTLSQTASLEIIKLFFTPEEALEDMKITDVFS--EDFFNSNFWLYWQTMFAFEPWHSAMEMRRYIARFIHHIGGLPDFSALKFTKYNQYESLALPLMNYLKEHGVTFQYATTVTNVEFEITAHKKVARKLVYVQRG-QEKHIDLTENDLVFITNGSNTESSTLGDNNTPAIM-----NTSL--GGSWELWKKIAEQDAA-FGRPDKFCGNIAESNW-ESATITTLDNRI-PPYIEKICKRDPFNGKVVTGGIVTVKDSKWLMSFTLNRQPHFKSQPKDQLVVWVYSLYCDVPGDYIKKPMKDCTGIEITEEWLYHMGVPEAEIHDLAVN-SANCVPCMMPYVTSYFMPRAAGDRPNVVPDGCVNAAFIGNFAETPRDTVFTTEYSVRTAMEAVYTLLDVDRGVPEVFGSAYDVRVLLDSTAKMMDGKKLTDM-ELPPAIQLAAMTGVQKISHTRIMQLLKEYQLV------------------------

>Paenibacillus tengchongensis

--------------------------------------------------------VNKEYGNRQVYFVGGGIAALAGAAYLVRDCGFPGQHIHIIEEMKVLGGSNDGAGSAEHGYVIRGGRMLNDETYENLWELLRTIPSLDQPGK-SVREEITGFDNANPTHSNARLVDKNGE-VVDVLSMGFDMADRLALGKLIITPEAAMGKARINDWFG--PHFFTTNFWYMWATTFAFQPWHSAAELKRYMLRFMHEFPRIQTLEGVTRTPYNQYDSIILPLQSYLEPFGVDFSLKCTVTDLEFK-EGDSITVTKLKVVRQG-VEGMIELGGDDLVIVTNGSMTESSSLGSM-TAAPR-----LNGK--GSSWKLWENIAAKKPG-LGNPEPFADHIDGSKW-ESFTVTFQDSRF-FDLMEKFTRNRAG-----TGALVTFKDSGWLMSVVLAFQPHFRNQPEHVRVFWGYGLYPDNVGDYVPKKMSECTGAEIMEELIGHLHF-GEHRDEIMAT--ANCIPCMMPFITSQFMPRLASDRPKVVPDGSTNLAFISQFCEIPDDVVFTEEYSVRAARLAVYTLLGVNRPVEPIRQYQYDVRTLLSGLVTSFR----------------------------------------------------------------

>Paenibacillus terrigena

----------------------------------------ILDSQTNTIEHMEVTSMKKEFGNRQVYFVGGGLASLAGATYLIRDCDFPGENIHILEGMHILGGSNDGAGDKMQGFVCRGGRMLNEETYENTWELLSSIPSMEQEGV-SVCDEILAFDHAHPTHANARLINKDGE-VLDVMSMGFNTADRLALGRLMITPEEKLDNLRICDWFADTPHFFETNFWYMWQTTFAFQKWSSLFEFRRYMNRMMFEFSRIQTLEGVTRTPYNQYDSIILPIKTYLDGYGVDFSLKYTVTDLDFA-EGDGITVTAIHYEQDG-KSGVIQLEKDDLCIVTNGCMTDNATLGDLNTPATM-----VYEN--PMSGNLWAKIAAKKEG-LGNPTPFFGNPEETNW-ESFTVTCKGDKL-LKMIEKYSRNKPG-----SGALMTFKDSNWLMSIVVAAQPHFKNQPADKTIFWGYGLYTDKEGNYVKKPMRDCTGKEMLIELLHHLHM-EEDMEEIMDS-VVNVIPCMMPYIVSQFQPRAMSDRPKVVPDGSTNLGLISQFVEIPEDMVFTEEYSIRAARIAVYTLMGLNKKVCPVTPHMYDVRTLLKALNTSYR----------------------------------------------------------------

>Paenibacillus thiaminolyticus

--------------------------------------------------------MSTEYNTKQVYFVGGGIASLAGAAFLVRDCDFPGSHIHIMEEMKILGGSNDGSGNGEQGYVIRGGRMLNDETYENTWDLLSSIPSIDDPAK-SVRDEIIEFDTAHPTHSNARLVNKHAE-VQDVTSMGFDMADRIAMSKLIMTPEEKLGTARIQDWFG--PHFFETNFWYMWATTFAFQPWHSAAELKRYMIRFMHEFPRIHTLEGVTRTPYNQYDSLTVPLHHYLTQHGVDFNMKCTVTDLDFK-EGRGITVTKIHYVKEG-SPGTIELNDGDLVIVTNGSMTEGYSLGSM-TSAPS-----LNGK--GSSWKLWDRIAAKKPG-LGNPSSFDDHIEGSKW-ESFTVTCQDSRF-FDRMEAFSRNKAG-----TGALVTFKESSWLMSIVLAHQPHFRNQPEHVRVFWGYGLFPDNEGDYVKKKMSDCTGEEILTELLNHLHF-HEDMDAMIQT--ANCIPCMMPYITAQFMPRAIGDRPQVVPQGSTNLAFVGQFCEIPNDVVFTEEYSVRTARIAVYTLLGINKPIAPINEYQYDIRTLLNGLVTSFR----------------------------------------------------------------

>Paenibacillus tianjinensis

--------------------------------------------------------VKKEYGNQQVYFVGGGIASLAGAVYLVRDCDFPGQNIHIIEEMPILGGSNDGAGDVEHGYVIRGGRMLNDEAYENLWELLNTIPSIDHPGQ-SVREDIIAFDTANPTHSNARLIDSKGE-VQDVLSMGFDMADRLAMGKLIITPEEAMGKARINDWFG--PHFFKTNFWYMWATTFAFQPWHSAVEFKRYMLRFMHEFPRIQTLEGVTRTPYNQYDSIILPLHKYLEPFGVDFTLKCTVTDLQFK-EGDGLTVTGMNVSRQG-VADVIEVNEGDLVIVTNGSMTEGSSLGSM-TTAPR-----LNGK--GSSWKLWENIAAKKPG-LGNPSAFDDHVDESKW-ESFTVTFQDSAF-FDLMEKFTRNRAG-----TGALVTFKDSSWFMSVVLAFQPHFRNQPEHVKVFWGYGLYPDNVGDFVKKRMCDCTGEEIMQELIGHLHF-DAHKDEIMAT--ANCIPCMMPYITSQFMPRLNSDRPKVVPEGSTNLAFISQFCEIPDDVVFTEEYSVRAARIAVYTLMGVNRPVEPINQYQYDVRTLLSGLVTSFR----------------------------------------------------------------

>Paenibacillus timonensis

--------------------------------------------------------MSAEHNTRQVYFVGGGIASLAGAAFLVRDCDFPGRNIHVLEEMKVLGGSNDGAGDGEHGYVIRGGRMLNDETYENTWDLLRSIPSIDQPGL-SVRDEIIAFDTAHPTHSNARLVNKQGQ-VEDVTSMGFDMADRLAMVKLIVTPEEKMGKARINDWFS--PHFFETNFWYMWATTFAFQPWHSAVELKRYMIRFMHEFPRIHTLEGVTRTPYNQYDSIILPLHKYLEERGVDFTLKCTVTDLDFK-EGDEITATRMHYIKDG-VAGTLDLAEGDLVIVTNGSMTEGYSLGSM-STAPK-----LNGK--GSSWQLWDRIAAKKTG-LGNPSSFDDHIDGSKW-ESFTVTCQDSRF-FDLMEAFSRNKAG-----TGALVTFKDSSWLMSIVLAHQPHFRNQPEHVKVFWGYGLFPDRVGDYVPKKMSECNGEEILTELLHHLHF-DQDREAIISS--ANCIPCMMPFITAQFMPRAIDDRPKVIPEGSTNLAFISQFCEIPDDVVFTEEYSVRAARMAVYGLLGLNKPIAPINHYQYDVRTLLQGFVTSFR----------------------------------------------------------------

>Paenibacillus tritici

--------------------------------------------------------VNKEYGNEQVYFVGGGIASLAGAAYLVRDCNFPGQNIHIIEEMKILGGSNDGAGDAQHGYVIRGGRMLNDETYENLWELLSTIPSIDQPGL-TLREEIVQFDESNPTRGQARLIDSAGV-VQDVTSMGFDMADRLTLGKLIITPEPALGTLRINDWFG--PHFFTTNFWYMWATTFAFQPWHSAVEFKRYMLRFMHEFPRIQTLEGVTRTPYNQYDSIILPLHQYLEPLGVDFTLKCTVTDLEFK-EGDGITVTAMKVVRAG-VEDMIQVHEGDRVIVTNGSMTEGSSLGSM-TSAPG-----MNGK--GSSWKLWETIAAKKPG-LGNPSSFDDHVDESKW-ESFTVTFQDSKF-FDLMEKFTRNRAG-----TGALVTFKDSSWLMSVVLAFQPHFRNQPEHVRVFWGYGLYPDRVGDYVHKKMCDCTGEEIMEELIGHLHF-EAHKEEIMAT--ANCIPCMMPYITSQFMPRLNSDRPQVVPEGSTNLAFIGQYCEIPDDIVFTEEYSVRAARIAVYTLFGLNRPVEPINAYQHDVRTLFSSLVTSFR----------------------------------------------------------------

>Paenibacillus tuaregi

--------------------------------------------------------MTADYRGRQVYFVGGGIASLAGAAFLVRDCGFPGEQIHIIEEMKILGGSNDGAGNGNEGYVIRGGRMLNDETYENTWNLLSSIPSLEHPGL-SVREEIIAFDTAHPTHSNARLVNKDGQ-VEDVTSMGFDMADRMAMLKLIMTPEAKMGKARINDWFD--PHFFETNFWYMWATTFAFQPWHSAVELKRYMIRFMHEFPRIHTLEGVTRTPYNQYDSIILPLHKYLEGFGVDFDMKCTVTDLDFK-EGEGITVTRMHIVKAG-AEMVVDIADGDLVIVTNGSMTESSSLGSM-TSAPE-----LNGK--GSSWKLWERIAAKKPG-LGNPSSFDDHIEGSKW-ESFTVTCQDSRF-FDLMEAFSRNKAG-----SGALVTFKDSSWLMSIVLAHQPHFRNQPEHVKVFWGYGLFPDRVGDYVKKPMAECTGEEILTELLHHLKF-EKDMDAIIAT--ANCIPCMMPFITAQFMPREITDRPEVVPAGSTNLAFISQFCEIPDDVVFTEEYSVRAARMAVYKLLGINKPVEPIHHYHYDVRTLLQGFVTSFR----------------------------------------------------------------

>Paenibacillus tundrae

--------------------------------------------------------VNNEYGNKQVYFVGGGIASLAGAAYLVRDCGFPGEHIHIIEEMNILGGSNDGAGNPDQGYIIRGGRMLNDEAYENLWELLASIPSIDRPGL-SVRQEITEFDHANPTHSNARLINRDGE-VEDVLSMGFDMADRLAMGKLIITPEDTLDKLRISDWFG--PHFFKTNFWYMWATTFAFQPWHSAVEFKRYMLRFFHEFPRIQTLEGVTRTPFNQYDSIILPLQKYLEPFGVDFTLKCTVTDLDFK-EGDGITVERMHVRRNG-NEDVIDIHEGDLVIVTNGSMTEGADIGSM-SSAPK-----LNGK--GSSWKLWENIAAKKPL-LGNPSSFNDHVDESKW-ESFTVTFQDSVF-FDLMEKFTRNRAG-----TGALVTFKDSSWFMSVVLAFQPHFRGQPEHVNVFWGYGLYPDNVGDYVKKRMCDCTGEEIMQELIGHLHF-QEHQEAIMAT--ANCIPCMMPYITAQFMPRLNSDRPKVVPDGSTNLAFISQFCEIPDDVVFTEEYSVRAARIAVYTLLGVNRPIEPIHQYQYDVRTLFSSFVTSFR----------------------------------------------------------------

>Paenibacillus turicensis

---------------------------------------------MGNYQRI-NPVVQEGISSKKAYLVGGGIASLAAAAFLIRDAHMPAGNIHILEQSDIYGGSMDGAGNAKDGYSARGGREI-EEHFECFMDLFGFIPSLTNPHR-TVLDEFRELNLEEPIESHCRLVEKAGK-TADFSTLGLSTSHALQLGKLAMLTEEKLGATTIEQFFD--PTFLETNFWYFWRSMFAFENWQSVVEVKRYMERFMHLISGMNQLKGILHTEYNQYDSLILPLLKWLEEQGVHFDLGHEVVDFDFDFSNGETTATAIHLKVNG-KPSTIKTTKNDIVMFTNGSMTQNSTRGDMDHPAII-----NDSVTDRGCWSVWEKIAPRSSQ-FGNPEVFCGDIDKTKW-LSFTITLTDDKILFPYLLDWTGDLPG-----MGGVVTIKDSSWMMSWTAPKQPHFINQPDNVKVLWAYGLLVDEEGDYIKKKMSDCTGQELFEELLYHMGL-KESIPDILKT-TTNVIPCMMPYITSQFMPRVLGDRPNVVPEGSTNFAFLGQFAEAPDDCVFTVEYSVRTAMMAVYSLLHLEKEVIPVHPSKFDVRVLLTAVRTCLGKKDLP-----------LDKTLGEIIGGTVLSKLL------------------------------

>Paenibacillus typhae

--------------------------------------------------------VAKEYGNRQVYFVGGGIASLAGAAYLVRDCDFPGNNIHIIEEMHILGGSNDGAGDVEQGYVIRGGRMLNDEAYENLWELLSTIPSIDHPGQ-SVREEIIAFDTANPTHSNARLIDRNGE-VQDVLSMGFDMSDRLAMGKLIITPEEAMGKARINDWFG--PHFFTTNFWYMWATTFAFQPWHSAVEFKRYMLRFMHEFPRIQTLEGVTRTPYNQYDSIILPLQKYLEPHGVDFSLKCTVTDLQFK-DCDGITVTGMNVTRMG-VEELIEVKEGDLVIVTNGSMTESASLGSM-TSAPE-----LNGK--GSSWKLWENIAGKKPG-LGNPSPFADYVDESKW-ESFTVTFQDSVF-FDLMEKFTRNRAG-----TGALVTFKDSSWFMSVVLAFQPHFRGQPEHVKVFWGYGLYPDKVGDFVNKRMCDCTGEEIMQELIGHLHF-EAHRDEIMAT--ANCIPCMMPYITSQFMPRLGTDRPEVVPDGSTNLAFISQFCEIPDDVVFTEEYSVRAARIAVYTLLGMGRPVEPIHQYHYDVRTLFASFVTSFR----------------------------------------------------------------

>Paenibacillus uliginis

---------------------------------------------------MEVTSMKKEYGNRQVYFVGGGLASLAGASYLVRDCDFPGENIHILEGMHILGGSNDGAGDTTNGFVCRGGRMLNEETYENTWELLSSIPSIEQEGV-SVCDEILAFDHAHPTHANARLINKDGE-VLDVMSMGFNTADRLALGRLMITPEEKLDNLRICDWFAETPHFFETNFWYMWQTTFAFQKWSSLFEFRRYMNRMVFEFSRIQTLEGVTRTPYNQYDSIILPIKKYLDGFGVDFSLKYTVTDLDFE-EGDGITVTAIHYEQDG-KPGIIQLREGDLCIVTNGCMTDNATLGDLNTPAKM-----VPEN--PMSGNLWAKIAAKKEG-LGNPAPFFGNAEETNW-ESFTVTCKGDKL-LKMIEKYSRNKPG-----SGALMTFKDSNWLMSIVVAAQPHFKNQPADTTIFWGYGLYTDKEGDYVKKPMRDCTGKEMLVELLHHLHM-EEDMDEIMDS-VVNVIPCMMPYIDSQFQPRAMSDRPKVVPDGSTNLAMISQFVEIPEDMVFTEEYSIRAARIAVYTLLELNKKVCPVTPHKYDVRTLLKALNTSYR----------------------------------------------------------------

>Paenibacillus woosongensis

----------------------------------------------------------MGTYSNQVYFVGGGIASLAGAAFLIRDCGFPGRQIHIIEELKVLGGSNDGAGDRLHGYVIRGGRMLNDETYENLWDLLSSIPSLDDPSQ-SVRDEIMAFDTVHPTHSRARLVNKDGE-VVDAASMGFDMGDRMDMAKLIITPEEQLGRLRINEWFG--AHFFQTNFWYMWATTFAFQPWHSAVELKRYMIRFMHEFPRIHTLEGVTRTPYNQYDSIILPLHRYLQEHGVDFEMNCTVTDLDFQ-AGDGITVTRMHYVKDG-VERALDLTEEDLVIVTNGSMTESSSLGSM-TAPPQ-----LYGK--GSSWQLWDRIAAKKPGLLGNPASFDDHIDESKW-ESFTVTCQGSRF-FDRMEKFSRNIAG-----SGALVTFKDSSWFMSIVLAHQPHFRNQPEDVKVFWGYGLYPDHVGDYVKKRMCDCTGEEILTELLHHLKF-ENELDELIRS--ANCIPCMMPYITAQFMPRTPGDRPQVVPEGSTNLALIGQFCEIPDDVVFTEEYSVRTARIAVYQLLGVNKPIQPINRYQYDIRTLLQSAAASFR----------------------------------------------------------------

>Paenibacillus wynnii

--------------------------------------------------------VKKEYGNQQVYFVGGGIASLAGAAYLVRDCDFPGQNIHIIEEMKILGGSNDGAGDVEEGYVIRGGRMLNDEAYENLWELLLTIPSIDHPGM-SVREEIIAFDTANPTHSNARLVNSKGE-VEDVLSMGFDMADRMALGKLILTPEDKMGKARINDWFA--PHFFETNFWYMWATTFAFQPWHSAVEFKRYMIRFIHEFPRIQTLEGVTRTPYNQYDSIILPMHKYLEPFGVDFTLKCTVTDLEFK-DGDGITVTKMNVLREG-VEDVIHVNEGDLVIVTNGSMTEGSSLGSM-TSAPQ-----LNGK--GSSWKLWENIAAKKPG-LGNPSSFDDHVDGSKW-ESFTVTFQDSKF-FDLMEKFSRNRAG-----TGALVTFKDSSWFMSIVLAFQPHFRNQPEHVKVFWGYGLYPDKVGDFVKKRMCDCTGEEIMEELIGHLHF-EEHKDEIMAT--ANCIPCMMPYITSQFMPRLNSDRPKVVPEGSTNLAFVGQYCEIPDDVVFTEEYSVRAARIAVYTLLGINRPIEPINQHQFDVRTLFTSFVTSFR----------------------------------------------------------------

>Paenibacillus xylanexedens

------------------------------------------------------MIVKKEHGNKQVYFVGGGIASLAGAAYLVRDCGFPGEHIHIIEEMPILGGSNDGAGNPDQGYIIRGGRMLNDEAYENLWELLASIPSIDRPGI-SVRQEITEFDDANPTHSNARLINRDGK-VEDVLSMGFDMADRLTMGKLIITPEDTLGKLRINDWFG--PHFFKTNFWYMWATTFAFQPWHSAVEFKRYMLRFFHEFPRIQTLEGVTRTPFNQYDSIILPLHNYLEPFGVDFTLKCTVTDLDFK-DGDGITVSRMHVLRGG-EEEVIDILEGDLVIVTNGSMTEGADIGSM-TSAPK-----LNGK--GSSWKLWENIAAKKPL-LGNPSSFNDHVDESKW-ESFTVTFQDSVF-FDLMEKFTRNRAG-----TGALVTFKDSSWFMSVVLAFQPHFRGQPEHVNVFWGYGLSPDNVGDYVKKRMCDCTGEEIMQELIGHLHF-QEHQEDIMAT--ANCIPCMMPYITAQFMPRLNSDRPKVVPEGSTNLAFISQFCEIPDDVVFTEEYSVRAARIAVYTLLGENRPIEPINKYQYDVRTLFSSFVTSFR----------------------------------------------------------------

>Paenibacillus zanthoxyli

--------------------------------------------------------VIQEYENKQVYFVGGGIASLAGAAFLIRDCDFPGSGIHIIEEMSILGGSNDGAGSEEQGYVIRGGRMLNDETYENLWDLLMSIPSLDHPGK-SVREEIIAFDNANPTHSNARLVNASGE-VVDVLSMGFDMADRLAIGKLIITPEEQMGKARISDWFG--PHFFTTNFWYMWATTFAFQPWHSAVELKRYMIRFMHEFPRIQTLEGVTRTPYNQYESIILPMKKYLEDHGVDFTLRCTVTDLDFK-EGDGITVTGLHVVRNG-AEEHIAVKEEDLVIITNGSMTESSSLGSM-TSAPR-----LNEK--GSSWKLWERIAVKKPG-LGNPSSFDDHIDGSKW-ESFTVTFSDTVF-FDLMEQFSRNRAG-----TGALVTFKDSSWLMSIVLAYQPHFRNQPEHVRVFWGYGLYPDKEGDFVKKKMSDCTGEEIMTELLGHLHF-EEHKEAIMAT--ANCIPCMMPFITAQFMPRAIGDRPKVVPDGSTNLAFIGQFCEIPDDVVFTEEYSVRTARIAVYTLLGVNKPITPINHYQYDVRTLLASLVTSFR----------------------------------------------------------------

>Saccharibacillus alkalitolerans

-----------------------------------------MYYSSGNYEAFARPMKPEGVDGKSAYLVGAGLASLAAACFLIRDGQMKGSRIHILEELDIAGGALDGGEEEHKGFVIRGGREM-EDHFECLWDLFHSIPSLEAEGA-SVLDEFYWLNKKDPNYSLMRATMNRGEDAHTDGKFTLSEKASMEIVKLFLTRDEDLYDLKITDVFT--EDFFASNFWLYWRTMFAFEDWHSALEMKLYIQRFIHHIGGLPDFSALKFTKYNQFESLVQPMLKYLEAHGVDFQYKTQVTNVVFEHHGTRKIAAKIQCLRAG-QPESIDLTEDDLVFVTNGSCTENSSLGDHHHAPVL-----NTQPGGGGCWELWRNIAAQDES-FGRPDKFCMNIEASKW-ESATITTLDDRI-PPYIEKICKRDPFSGKVVTGGIVSVKDSSWMLSWTLNRQPHFKKQPKDQLVVWFYALFTDVPGDYIKKPMQDCTGEEITAEWLYHMGVPEADIPEMAAR-SANCIPCMMPYITAFFMPRTAGDRPKVVPDGCVNFAFIGQFADTERDTVFTTEYSVRTAMESVYTLLNVDRGVPEVFASEFDIRVLLDSTSKMMDGKKLTDL-KQPVLLKLLEQGALKKASGTVIGDLLKKYGIV------------------------

>Saccharibacillus deserti

-----------------------------------------MYYSSGNYEAFARPMKPEGVDGKSAYLVGAGLASLAAACFLIRDGQMKGSHIHILEELKIPGGALDGSEEAGKGFVIRGGREM-EDHFECLWDLFHSIPSLEVENA-SVLDEFYWLNKKDPNYSLMRATVNRGEDAHTDGKFTLSEKASMEIVKLFLTRDEDLYDLKITDAFT--EEFFASNFWLYWRTMFAFEDWHSALEMKLYIQRFIHHIGGLPDFSALKFTKYNQFESLVQPMLKYLEEHGVQFVYDTRVTNVVFERRGTRKVAAKIECMRGG-QPESIDLTEDDLVFVTNGSCTENSSLGDHHHPAVL-----KTEP--GGCWELWRSIAAQDES-FGRPDKFCTNIDETKW-ESATITTLDGRI-PPYVEKICQRDPFSGKVVTGGIVSVKDSSWMLSWTLNRQPHFKKQPKDQLVVWFYALYTDVPGDYIKKPISACTGEEITAEWLYHMGVPEADIPEMAAN-SANCIPCMMPYITAFFMPRTAGDRPKVVPDGCVNFAFIGQFADTERDTVFTTEYSVRTAMESVYTLLGVDRGVPEVFASEYDIRVLLDSTSKMMDGRKLAEL-KQPMLLKLLEQGALKKASGTVVGELLKKYGIV------------------------

>Saccharibacillus qingshengii

-----------------------------------------MYYSSGNYEAFARPVKPEGVDGKSAYLVGAGLASLAAACFLIRDGQMKGSHIHILEELKMPGGALDGSEEAGKGFVIRGGREM-EDHFECLWDLFHSIPSLEVENA-SVLDEFYWLNKKDPNYSLMRATVNRGEDAHTDGKFTLSEKASMEIVKLFLTRDEDLYDLKITDVFT--EEFFASNFWLYWRTMFAFEDWHSALEMKLYIQRFIHHIGGLPDFSALKFTQYNQFESLVQPMLKYLEEHGVQFVYDTRVTNVVFEQHGTRKVAAKIECMRGG-QPESIDLTEDDLVFVTNGSCTENSSLGDHRHPAVL-----KTEP--GGCWELWRSIAAQDES-FGRPDKFCTNIDETKW-ESATITTLDDRI-PPYIEQICQRDPFSGKVVTGGIVSVKDSSWMLSWTLNRQPHFKKQPKDQLVVWFYALYTDVPGDYIKKPISACTGEEITAEWLYHMGVPEADIPEMAAN-SANCIPCMMPYITAFFMPRTAGDRPKVVPDGCVNFAFIGQFADTERDTVFTTEYSVRTAMESVYTLLGVDRGVPEVFASEYDIRVLLDSTSKMMDGKKLTEL-KQPMLLKLLEQGALRKVSGTVVGELLKKYGIV------------------------

>Salipaludibacillus agaradhaerens

--------------------------------------------------------MTKRNDERHVYFVGGGLASLAGAAYLIRDCGFKGENIHVLEGLSVLGGSNDGGGSPIKGFVCRGGRMLNEETYENFWELFSSIPSLEEEGM-SVTEEILAFDDAHPTHSNARLINKQGK-ILDVMSMGFNNEDRIALGKLLLTPEEKLDHLKISDWFG--THFFETHFWYMWQTTFAFQKWSSLFEFKRYMNRMIFEYSRIHTLEGVTRTKYNQYESVILPLKTFLDNHGVDFSLKCTVTDLDFA-NTQEITVSTIHYTTEK-GEGTISLKEKDLCIVTNGCMTDCATLGDLHTPAPF-----NAED--PISGNLWANIAAKKQG-LGNPKPFFSDPGQTNW-QSFTVTLKGNKL-LKRIEAFSRNVPG-----SGALMTFKDSSWLMSIVVAHQPHFKGQTEDETIFWGYGLYTDQLGDYVKKPMRECTGEEILIELLHHLHF-DKDVADILAD-VVNVIPCIMPYIVAQFQPRAMTDRPKVVPEGSTNFAMISQFVEIPEDMVFTEEYSVRAARIAVYELLGITKKICPVTPHQYDIRTLFKAINTTFR----------------------------------------------------------------

>Sporolactobacillus pectinivorans

---------------MVKKRTGILAAGAAVIGITYAAKKWQQSIKSMKEKTADEEIKSRYYGEKQVYFIGGGIASLAGAAFLVRDAHFDGHNIHVIEGMPVLGGSNDGAGSAAQGFVCRGGRMLNEETYENFWDLFKSIPSLAMPGK-SVTEEILNFDHLHPTHAQARLIDKERN-ILDAHSMGFDNDDRIAMIRLLATKENKLDNLTIQDWFG--PHFFETNFWYMWQTTFAFQKWSSLFELRRYMNRMILEFSRIDTLEGVTRTPYNQYESLILPLKAYLDKYGVDFSINRTVTDVDFK-DDDTITATALHFDDGS----SLKLKEGDIVIMTNACMTDSATLGDWDVPAPI-----PEER--PISGELWYKIAHKKAN-LGNPEPFFTHEKETNW-ESFTVTCKGSKL-LKRIEKFTDNIPG-----SGALMTFKDSNWLMNIVIAAQPHFKAQDADTTIFWGYGLYTDRVGDYVKKPMKECSGEEILYELICHLKW-EEDWNEIKEE-VVNVIPCYMPYIDAQFQPRAMSDRPSVVPEGSTNFAMISQFVEIPKDMVFTEEYSVRAARIAVYSLFHIDKEICHVTPYNRSPKVLAKAIQTMYR----------------------------------------------------------------

>Staphylococcus agnetis

-----------------------------------------MYYSKGNYEAFARPKKPENVEKKSAYLVGSGLASLAAACFLIRDGQMEGTQIHILEEMSKPGGSLDGDYLPVKGYVVRGGREM-ENHFECLWDLFRSIPSLEVKGA-SVLDEFYWLNKEDPNYSRCRVIEKRGQRLATDGDFTLTPTALKEIVKLCLTNEADLDDVSITEVFS--KDFFDSNFWIYWKTMFAFEPWHSAMEMRRYLLRFIHHINGLADFSSLKFTKYNQYESLVMPMIHYLRAHQVQFDYNVEVEDIKVDVTTSQKVARELLIKRDG-QRETLKLTPDDLIFVTNGSITESSTYGDNFTPAPP-----SHEL--GGSWKLWQNLAKQSPE-FGNPEKFFKNIPPKSWFVSATATTNHEKV-IRQIETLCKRDPLAGKTVTGGIITVHDSSWQLSFTVNRQQQFKSQPEDQVSTWIYALYSDVKGDYIKKPITECSGHEICQEWLYHMGMPEDQIEQLAKD-EINTIPVYMPYITAYFMPRALGDRPRVVPHQSRNLAFIGNFAETERDTVFTTEYSVRTAMEAVYQLLNIDRGVPETIGTEFDVRELMKAMYHLNDRKGLSEITKTNKVQRTLFKGIKRKIKGTYIETLLKTYHLL------------------------

>Staphylococcus argensis

-----------------------------------------MYYTNSNYEAFARPKKPENVDKKSAYLIGSGLASLAAACFLIRDGQMKGKNIHILEELDISGGSLDGINMENHGFVVRGGREM-ENHFECLWDLFRSIPSLEQPDA-SVLDEFYWLNKEDPNYSKCRAIYSGGKRIDTDGDFTLSKKAIKEILSLCMKKEEDLEDVKISEVFT--EDFLNSNFWLYWKTMFAFEPWHSAMEMRRYLMRFVHHIGGLADFSALKFTKYNQYESLVLPMVEYLKSHGVHFEYGVQVDNILVDSTSSTKIAREMLVNKNG-KTESIPLTLDDLVFVTNGSITESSTYGDNDHPAPP-----TKAL--GGSWTLWKNLANQSPE-FGRPEKFCDHIPDKSWLVSATTTTNNKKV-ISYIEKLSKRDVLSGRTVTGGIVTVADSSWQLSFTVNRQQQFKEQPKDQVSVWIYALYSDVKGDFIKKPITECTGSEICQEWLYHMGVPQDELVELAQT-ECNTIPVYMPYVTAYFMPRAYKDRPLVVPNGSKNLAFIGNFAETERDTVFTTEYSVRTAMEAVYQLLEVDRGVPEVYASEFDARVLMDAYYQLNDRKSLPELVNNNFIKRSVLKNAMKKIQGTFIEELLRKHKLL------------------------

>Staphylococcus aureus

-----------------------------------------MYYSYGNYEAFARPKKPENVENKSAYLIGSGLASLAAACFLIRDGQMEGSKIHILEELPKAGGSLDGENMPLKGYVVRGGREM-ENHFECLWDLFRSIPSLEIDNA-SVLDEFYWLNKEDPNYSRCRVIEKQGQRLVTDGDFTLTKTAIKEILDLCLTNEEDLDDVKITDVFS--DDFFNSNFWIYWKTMFAFEPWHSAMEMRRYLMRFVHHISGLADFSALKFTKYNQYESLVLPMVEYLKSHGVQFEYDVKVEDIKIDVTTSQKIAREILIDRNG-NAESIKLTINDLVFVTNGSITESSTYGDNDTPAPP-----TDEL--GGSWTLWKNLARQSPE-FGNPDKFCQNIPKKSWFVSATSTTNNKEI-IDTIESICKRDPLAGKTVTGGIITINDSAWQMSFTINRQQQFKDQPENEISTWIYALYSDVNGDYIKKPITECSGNEICQEWLYHLGVSTDKIEDLAKH-ASNTIPVYMPYITSYFMTRAIGDRPLVVPHQSQNLAFIGNFAETERDTVFTTEYSVRTAMEAVYQLLNIDRGIPEVINSPFDLRVLMDAIYELNDHQDLREITKDSKMQKLALAGFLKKIKGTYIESLLKEHKLL------------------------

>Staphylococcus borealis

-----------------------------------------MYYSNGNYEAFARPKKPENVAHKSAYLIGSGLASLAAACFLIRDGQMDGSKIHVLEELSKPGGSLDGDQLPLKGYVVRGGREM-ENHFECLWDLFRSIPSLEMDNA-SVLDEFYWLNKEDPNYSRCRVIEKRGHQLPTDGDFTLTPQAIKEIIHLCLMKEEALNDVTITDVFS--NDFMHSNFWIYWKTMFAFEPWHSAMEMRRYLMRFVHHIGGLADFSALKFTKYNQYESLVLPMIAYLESHGVQFQYDVQVMNIKVDITTTEKVAREIQLKRSG-KDEVIPLTPNDLVFVTNGSITESSTYGDNNTPAPP-----TKDI--GGSWTLWRNLAKQSPE-FGCPEKFYRHLPDKSWFVSATATTNNKTI-INTIEHICKRDPLAGKTVTGGIVTVNDSKWQLNFTVNRQQQFKSQPDDEVSVWIYALYSNVNGDFINKPITECSGNEICQEWLYHMGVPINQIEDLAKN-QCNTIPVYMPYICSYFMPRAVGDRPLVVPKNARNIAFIGNFAETERDTVFTTEYSVRTAMEAVYQLLGIDRGVPEVVATEFDLRILMDALYELTDRKNLLELTEHNKIQQLALNTFLKKIKGTYIEALLQQHKLL------------------------

>Staphylococcus chromogenes

-----------------------------------------MYYSKGNYEAFARPKKPENVEKKSAYLVGSGLAALAAACFLIRDGQMQGSQIHILEELPKPGGSLDGDQLPMKGYVVRGGREM-ENYFECLWDLFRSIPSLEVKDA-SVLDEFYWLNKEDPNYSRCRLIEKQGHELPTDGDFKLTSKAQKEILKLCLTNEEDLNDVKITDVFT--QDFFDSNFWTYWKTMFAFEPWHSAMEMRRYLMRFVHHIGGLSDFSALKFTKYNQFESLVMPMVHYLRDHNVQFEYDVEVEDIKVDITTSQKVAREIVMKRDG-KRESISLTPDDLVFVTNGSITESSTYGDNDTPAPP-----TEEI--GGSWQLWQNLAKQSPE-FGNPEKFYRNIPKKSWFVSATATTSNKTV-IQAIEKLCKRDPLAGKTVTGGIITVDDSNWQMSFTVNRQQQFKSQPKDQVSTWIYALYSDVNGDYIKKPITECSGHEICQEWLYHMGVPVDQIEQLAKE-EVNTIPVYMPYITAYFMPRAQGDRPLVVPHQSRNLAFIGNFAETERDTVFTTEYSVRTAMEAVYQLLNIDRGVPETIGTEFDVRELMNAMYHLNDRQSLDEVMQDSKVQRTLLKGVKRKIKGTYVETLLKDYHLL------------------------

>Staphylococcus coagulans

-----------------------------------------MYYSKGNYEAFARPRKPENVENKSAYLVGSGLAALAAACFLIRDGQMKGSQVHILEELPKPGGSLDGDELPNKGYVVRGGREM-ENHFECLWDLFRSVPSLEVENA-SVLDEFYWLNKEDPNYSRCRMIENRGHRVDTDGDFTLTQKAIKEILDLCLMNEEDLDDVKITDVFS--EDFLKSNFWVYWKTMFAFEPWHSAMEMRRYLMRFVHHIGGLADFSALKFTKYNQYESLVLPMVEYLKSHDVQFEYNIKVEDIKVDVTTSQKLAREILIKRDG-KDDVIQLTPNDLVFVTNGSITESSTYGDNDTPAPI-----TDDI--GGSWLLWNNLAKQSPE-FGNPEKFYKDLPKRSWFISATATTNNKKI-IGAIERLCKRDPLSHKTVTGGIITVNDSSWQMSFTVNRQQQFKSQPDDQVSTWIYALYSDVDGDYIKKPITECSGSEICQEWLYHMGVPTEEIEYLATE-EANTIPAYLPYITSYFMPRAVGDRPLVVPHQSRNLAFIGNFAETERDTVFTTEYSVRTAMEAVYQLLDIDRGVPEVIASEFDLRVLMDAAYELNDRKSLQEITENSKIQKVALSGFLRKIKGTYIETLLKDYHLL------------------------

>Staphylococcus condimenti

-----------------------------------------MYYTNSNYEAFARPKKPENVDKKSAYLIGSGLASLAAACFLIRDGQMKGENIHILEELNISGGSLDGINMEHPGFVVRGGREM-ENHFECLWDLFRSIPSLEQPDA-SVLDEFYWLNKEDPNYSKCRAIYSGGKRIDTDGDFTLSKKAIKEILSLCMKKEEDLEDVKISEVFT--EDFLNSNFWLYWKTMFAFEPWHSAMEMRRYLMRFVHHIGGLADFSALKFTKYNQYESLVLPMVEYLKSHGVHFEYGVQVDNILVDSTSSTKIAREILVNKNG-KTESIPLTLDDLVFVTNGSITESSTYGDNDHPAPP-----TKAL--GGSWTLWKNLANQSPE-FGRPEKFCDHIPDKSWLVSATTTTNNKKV-ISYIEKLSKRDVLSGRTVTGGIVTVADSSWQLSFTVNRQPQFKEQPKDQVIVWIYALYSDVKGDFIKKPITECTGSEICQEWLYHMGVPQDQLVELAQS-ECNTIPVYMPYVTAYFMPRAYKDRPLVVPNGSKNLAFIGNFAETERDTVFTTEYSVRTAMEAVYQLLEVDRGVPEVYASEFDVRVLMDAYYQLNDRKSLPELVNKNFLKRSVLKKAMEKIHGTFIEELLRKHKLL------------------------

>Staphylococcus croceilyticus

-----------------------------------------MYYSNGNYEAFARPKKPENVEGKSAYLIGSGLASLAAACFLIRDGQMDGSKIHVLEELSKPGGSLDGAELPMKGYVVRGGREM-ENHFECLWDLFRSIPSLEIEGA-SVLDEFYWLNKEDPNYSRCRVIEKRGHQLATDGDFTLTKQAIKEIVALCLKKEEELNDVKISDVFS--NDFMHSNFWIYWKTMFAFEPWHSAMEMRRYLMRFVHHIGGLADFSALKFTKYNQYESLVRPMIAYLKSHGVQFQYDVQVLDIKVDVTTKEKVAREIQLKRQG-QLETITLTPDDFVFVTNGSITESSTYGDNDTPASP-----NHQP--GGSWNLWRNLAKQSPE-FGCPEKFYQDLPDKSWFVSTTITTNNKTI-IDTIERICKRNPLSGKTVTGGIITVNDSNWQMSFTINRQQQFKTQPKDEMSVWVYALYSNVEGDYIHKPIVECSGSEICQEWLYHIGISDNLIENLANN-HCNSIPVYMPYICSYFMPRAVGDRPLVVPKDSRNLAFIGNFAETERDTVFTTEYSVRTAMEAVYQLLDIDRGVPEVVASEFDLRVLMDALYELTDRKDLTELVEQNRIQQFALNAFLKKIKNTYIEEMLQQHKLL------------------------

>Staphylococcus durrellii

-----------------------------------------MYYTNSNYEAFARPKKPENVDKKSAYLIGSGLASLAAACFLIRDGQMKGENIHILEELNISGGSLDGINMEHHGFVVRGGREM-ENHFECLWDLFRSIPSLEQPEA-SVLDEFYWLNKEDPNYSKCRAIYSGGKRIETDGDFTLSKKAIKEILSLCMKKEEDLEDVKISEVFT--EDFLNSNFWLYWKTMFAFEPWHSAMEMRRYLMRFVHHIGGLANFSALKFTKYNQYESLVLPMIEYLKSYGVHFEYGVQVDNILVDSTSSKKIARELLINKNG-KTESIPLTLDDLVFVTNGSITESSTYGNKDQPAPP-----TKAL--GGSWTLWKNLANQSPE-FGRPEKFCDYIPNKSWLVSATTTTNNKKV-ISYIEKLSKRDVLSGRTVTGGIVTVADSSWQLSFTVNRQQQFKEQPKDQASIWIYALYSDVKGDFIKKPITECTGSEICQEWLYHMGVPQDELVELAQT-ECNTIPVYMPYVTAYFMPRAYKDRPLVVPNGSKNLAFIGNFAETERDTVFTTEYSVRTAMEAVYQLLEVDRGVPEVYASEFDVRVLMEAYYQLNDRKSLPELVNKNFLKRSVLKKAMEKIHGTFIEELLRKHKLL------------------------

>Staphylococcus epidermidis

-----------------------------------------MYYTNSNYEAFARPKKPENVDNKSAYLIGAGLASLAAACFLIRDGQMKGENIHILEELDISGGSLDGINMEHHGYVVRGGREM-ENHFECLWDLFRSIPSLEQTNA-SVLDEFYWLNKEDPNYSKCRAIESGGKRIDTDGDFTLTKKAIKEILNLCLMKEEDLDDVKITDVFS--RDFLNSNFWLYWKTMFAFEPWHSAMEMRRYLMRFVHHIGGLADFSALKFTKYNQYESLVLPMIEYLKSHSVNFEFGVQVNNILVDATPSTKIAREIILTRDD-KEESIPLTVNDLVFVTNGSITESSTYGDNDHPAPI-----THSL--GGSWTLWKNLANQSPE-FGRPEKFCDHIPAKSWFVSATATTDNKKI-ISYIEQLCKRDVLSGRTVTGGIISVANSSWQLSFTVNRQQQFKKQPKNQVSVWIYALYSDEKGDFIKKPITECTGSEICQEWLYHMGVPQEEIVELAQS-ECNTIPVYMPYVTAYFMPRAYKDRPLVVPNGSKNLAFIGNFAETARDTVFTTEYSVRTAMEAVYQLLDVDRGVPEVYASEFDARVLMDAFYELNDRKSLHELVNKNFIKRSVLNTVLKKIRGTFIEELLRKHKLL------------------------

>Staphylococcus haemolyticus

-----------------------------------------MYYSNGNYEAFARPEKPENVEVKSAYLIGSGLASLAAACFLIRDGQMDGSKIHVLEELSKPGGSLDGDELPLKGYVVRGGREM-ENHFECLWDLFRSIPSLEMDNA-SVLDEFYWLNKEDPNYSRCRVIEKRGHQLSTDGDFTLTPQAIKEIIHLCLMKEETLNDVKITDVFS--NDFLHSNFWIYWKTMFAFEPWHSAMEMRRYLMRFVHHIGGLADFSALKFTKYNQYESLVLPMIAYLESHGVQFQYDVQVLNIKVDITTKEKVAREIQLKRSG-KDDIIPLTPDDLVFVTNGSITESSTYGDNNTPAPP-----TKEI--GGSWTLWRNLAKQSPE-FGCPEKFYHRLPDKSWFVSATATTNNMTI-INTIEQICKRDPLAGKTVTGGIVTVNDSKWQMSFTVNRQQQFKSQPEDEVSVWIYALYSNINGDYIHKPITECSGNEICQEWLYHMGVPINQIEDLAKT-QCNTIPVYMPYICSYFMPRSIGDRPLVVPENARNIAFIGNFAETERDTVFTTEYSVRTAMEAVYQLLDIDRGVPEVVATEFDLRILMDALYELNDGKNLSELSEHNKIQKLALNAFLKKIKGTYIEELLHQHKLL------------------------

>Staphylococcus hominis

-----------------------------------------MYYSNGNYEAFARPKKPEHVEQKSAYLIGSGLASLAAACFLIRDGQMDGSNIHILEELSKSGGSLDGTELPMKGYVMRGGREM-ENHFECLWDLFRSIPSLEIEDA-SVLDEFYWLNKEDPNYSRCRVIEERGQRLPTDGDFTLTKQAMKDILQLCLMKEEDLNDVTISDVLS--EDFMNSNFWIYWKTMFAFEPWHSAMEMRRYLMRFVHHIGGLADFSALKFTKYNQYESLVRPMVAYLTSHGVQFEYNVQVLDVKVDVTTKDKVAKTIELKRNG-NKETIQLTPDDLVFITNGSITESSTYGDNDTPAPP-----TKDI--GGSWNLWRNLAKQSPE-FGNPEKFYKDLPDKSWFVSATVTTNNKTV-IEAIERLCKRDPLAGKTVTGGIITVNDSKWQMSFTVNRQQQFKQQPKDEVSVWVYALYSDEKGDYIHKPIVECSGHEICQEWLYHMGLPIDQIECLATQ-HCNTIPVYMPYICSYFMPRAIGDRPLVVPQHSRNLAFIGNFAETERDTVFTTEYSVRTAMEAVYQLLDIDRGVPEVMATEFDIRVLLDAMYELNDRKDLKTLAEQNKIQQVALHTFLKKIKGTFVEELLKQHKLL------------------------

>Staphylococcus hyicus

-----------------------------------------MYYSKGNYEAFARPKKPENVEKKSAYLVGSGLASLAAACFLIRDGQMEGTQIHILEEMSKPGGSLDGDYLPVKGYVVRGGREM-ENHFECLWDLFRSIPSLEVKGA-SVLDEFYWLNKEDPNYSRCRVIEKRGQRLATDGDFTLTPTALKEIVKLCLTNEADLDDVSITEVFS--KGFFDSNFWIYWKTMFAFEPWHSAMEMRRYLLRFIHHINGLADFSSLKFTKYNQYESLVMPMIHYLRAHQVQFDYNVEVEDIKVDVTTSQKVARELLIKRDG-QRETLKLTPDDLVFVTNGSITESSTYGDNFTPAPP-----SHEL--GGSWKLWQNLAKQSPE-FGNPEKFFKNIPPKSWFVSATATTNHEKV-IRQIETLCKRDPLAGKTVTGGIITVHDSSWQLSFTVNRQQQFKSQPEDQVSTWIYALYSDVKGDYIKKPITECSGHEICQEWLYHMGMPEDQIEQLAKD-EINTIPVYMPYITAYFMPRALGDRPRVVPHQSRNLAFIGNFAETERDTVFTTEYSVRTAMEAVYQLLNIDRGVPETIGTEFDVRELMKAMYHLNDRKGLSEITKTNKVQRTLFKGIKRKIKGTYIETLLKTYHLL------------------------

>Staphylococcus lugdunensis

-----------------------------------------MYYSNGNYEAFARPKKPENVEQKSAYLIGSGLASLAAACFLVRDGQMDGAKIHVLEELAKPGGSLDGDELPMKGYVVRGGREM-ENHFECLWDLFRSIPSLEVEGA-SVLDEFYWLNKEDPNYSRCRVIEKRGHQLPTDGDFTLTKQAIKEMIDLCLMTEKDLNDVMIFEVFS--KDFMNSNFWIYWKTMFAFEPWHSAMEMRRYLMRFVHHIGGLADFSALKFTKYNQYESLVLPMIAYLESHGVQFEYDVQVLDIKVDVTTTEKVAREMKLKRQG-KEETMLLTPNDLVFVTNGSITESSTYGDNEIPAPP-----TKVT--GGSWNLWRNLAKQSPE-FGHPEKFYQDLPEKSWFVSATATTNNKII-IDTIESICKRDPLAGKTVTGGIITVNDSAWQMSFTVNRQQQFKAQPKDEVSVWIYALYSNVKGDYINKPIVECSGNEICQEWLYHMGVPTNQIKDLAQN-QCNSIPVYMPYICSYFMPRAIGDRPLVVPHQSRNLAFIGNFAETERDTVFTTEYSVRTAMEAVYQLLNIDRGVPEVVATEFDLRVLMDAMYELNDGKGLVELVEQKKLKQVALNAFLKKIKGTYIEQLLKQHKLL------------------------

>Staphylococcus nepalensis

-----------------------------------------MYYTNSNYEAFARPKKPENVDKKSAYLIGSGLASLAAACFLIRDGQMKGENIHILEELNISGGSLDGINMEHHGFVVRGGREM-ENHFECLWDLFRSIPSLEQPEA-SVLDEFYWLNKEDPNYSKCRAIYSGGKRIETDGNFTLSKKAIKEILSLCMKKEEDLEDVKISEVFT--EDFLNSNFWLYWKTMFAFEPWHSAMEMRRYLMRFVHHIGGLANFSALKFTKYNQYESLVLPMIEYLKSYGVHFEYGVQVDNILVDSTSSKKIARELLINKNG-KKESIPLTLDDLVFVTNGSITESSTYGNNDHPAPP-----TKAL--GGSWTLWKNLANQSPE-FGRPEKFCDYIPNKSWLVSATTTTNNKKV-ISYIEKLSKRDVLSGRTVTGGIVTVADSSWQLSFTVNRQQQFKEQPKNQASIWIYALYSDVKGDFIKKPITECTGSEICQEWLYHMGVPQDELVELAQT-ECNTIPVYMPYVTAYFMPRAYKDRPLVVPNGSKNLAFIGNFAETERDTVFTTEYSVRTAMEAVYQLLEVDRGVPEVYASEFDVRVLMDAYYQLNDRKSLPELVNKNFLKRSVLKKAMEKIHGTFIEELLRKHKLL------------------------

>Staphylococcus petrasii

-----------------------------------------MYYSNGNYEAFARPKKPENVEGKSAYLIGSGLASLAAACFLIRDGQMDGSKIHVLEELSKPGGSLDGTELPMKGYVVRGGREM-ENHFECLWDLFRSIPSLEIEGA-SVLDEFYWLNKEDPNYSRCRVIEKRGHQLATDGDFTLTKQAIKEIVALCLKKEEDLNDVKISDVFT--NDFMHSNFWIYWKTMFAFEPWHSAMEMRRYLMRFVHHIGGLADFSALKFTKYNQYESLVRPMIAYLKSHGVQFQYDVQVLDIKVDVTTKEKVAREIQLKRQG-QLETIALTPNDLVFVTNGSITESSTYGDNDTPAPP-----NHQP--GGSWNLWRNLVKQSPE-FGCPEKFYQDLPDKSWFVSATVTTNNKTI-IDTIERICKRNPLSGKTVTGGIITVNDSNWQMSFTINRQQQFKTQPKDEMSVWVYALYSNVEGDYIHKPIVECSGSEICQEWLYHIGIPENLIENLANI-HCNSIPVYMPYICSYFMPRAVGDRPLVVPKDSRNLAFIGNFAETERDTVFTTEYSVRTAMEAVYQLLDIDRGVPEVVASEFDLRVLMDALYELNDRKDLTELAEQNRIQQFALNAFLKKIKNTYIEELLQQHKLL------------------------

>Staphylococcus pragensis

-----------------------------------------MYYSNGNYEAFARPKKPENVEGKSAYLIGSGLESLAAACFLIRDGQMDGSKIHVLEELSKPGGSLDGAELPMKGYVVRGGREM-ENHFECLWDLFRSIPSLEIEGA-SVLDEFYWLNKEDPNYSRCRVIEKRGHQLATDGDFTLTKQAIKEIVALCLKREADLNDVKISDVFS--NDFMHSNFWIYWKTMFAFEPWHSAMEMRRYLMRFVHHIGGLADFSALKFTKYNQYESLLRPMIAYLKSHDVQFQYDVQVLDIKVDVTTKEKVAREIQLKRQG-QLETITLTPNDFVFMTNGSITESSTYGDNDTPASP-----DHQP--GGSWNLWRNLAKQSPE-FGYPEKFYQDLPDKSWFVSATVTTNNKTI-IDTIERICKRNPLSGKTVTGGIITVNDSNWQMSFTINRQQQFKTQPKDEMSVWVYGLYSNVEGDYIHKPIIECSGSEICQEWLYHIGIPDNQIENLAHN-HCNSIPVYMPYICSYFMPRAVGDRPLVVPKDSRNLAFIGNFAETERDTVFTTEYSVRTAMEAVYQLLDIDRGVPEVVASEFDIRVLMEALYELNDRKNLTELAEQNKIQQFALNAFLKKIKNTYIEELLQQHKLL------------------------

>Staphylococcus schleiferi

-----------------------------------------MYYSKGNYEAFARPRKPENVENKSAYLVGSGLAALAAACFLIRDGQMKGSQVHILEELPKPGGSLDGDELPNKGYVVRGGREM-ENHFECLWDLFRSVPSLEVENA-SVLDEFYWLNKEDPNYSRCRMIENRGHRVDTDGDFTLTQKAIKEIVDLCLMNEEDLNDIKITDVFS--EDFLKSNFWVYWKTMFAFEPWHSAMEMRRYLMRFVHHIGGLADFSALKFTKYNQYESLVLPMVEYLKSHDVQFEYNIKVEDIKVDVTTSQKLAREILIKRDG-KDDVIPLTPNDLVFVTNGSITESSTYGDNDTPAPI-----TDDI--GGSWLLWNNLAKQSPE-FGNPEKFYKDLPKRSWFISATATTNNKKI-IGAIERLCKRDPLSHKTVTGGIITVNDSSWQMSFTVNRQQQFKSQPDDQVSTWIYALYSDVDGDYIKKPITECSGSEICQEWLYHMGVPTEEIEYLATE-EANTIPAYLPYITSYFMPRAVGDRPLVVPHQSRNLAFIGNFAETERDTVFTTEYSVRTAMEAVYQLLDIDRGVPEVIASEFDLRVLMDAAYELNDRKSLQEITENSKIQKVALSGFLRKIKGTYVETLLKDHHLL------------------------

>Staphylococcus schweitzeri

-----------------------------------------MYYSYGNYEAFARPKKPENVENKSAYLIGSGLASLAAACFLIRDGQMEGSKIHILEELPKAGGSLDGENIPLKGYVVRGGREM-ENHFECLWDLFRSIPSLEIDNA-SVLDEFYWLNKEDPNYSRCRVIEKQGQRLVTDGDFTLTKKAIKEILDLCLTNEEDLDDVKITDVFS--DDFFNSNFWIYWKTMFAFEPWHSAMEMRRYLMRFVHHIGGLADFSALKFTKYNQYESLVLPMVEYLKSHGVQFEYDVKVEDIKVDVTTSQKIAREILVHRNG-KAESIKLTVNDLVFVTNGSITESTTYGDNNTPAPP-----TDEL--GGSWTLWKNLARQSPE-FGNPDKFCQNIPQKSWFVSATSTTNNKDI-IDTIESICKRDPLAGKTVTGGIITINDSTWQISFTINRQQQFKDQPKDEISTWIYALYSDVNGDFIKKPITECSGNEICQEWLYHLGVPTDKIEDLAKH-ASNTIPVYMPYITSYFMTRAIGDRPLVVPHQSQNLAFIGNFAETERDTVFTTEYSVRTAMEAVYQLLNIDRGVPEVINSTFDLRVLMDAVYELNDHQDLREITKDSKMQKLALAGFLKKVKGTYIETLLKEHKLL------------------------

>Viridibacillus soli

-----------------------------------------MYYSNGNYEAFARPKKPEGVDEKSAYLIGSGLASLSAACFLIRDGQMKGENIHILEELDISGGSLDGILNPTRGFIIRGGREM-EDHFECLWDLFRSIPSLEIENA-SVLDEFYWLNKEDPNYSKCRLIEKRGQRLEDDGKFTLSDKSSEEMIKLFFTPEEKLEDKKITDVFS--EEFFESNFWLYWSTMFAFEKWHSAMEMRRYIVRFIHHIGGLPDLSALKFTKYNQYESLVLPMINYLEKHNVDFQYNTVVENVLVDRVGDKKVAHTLVLKQNG-EKKNIELTENELVFVTNGSITESTTYGDNNTPAPI-----STDL--GGSWSLWKNIASQDSE-FGRPEKFCDDLPEESWFVSATLTTLDAKV-APYIEKISKRDPYAGKVVTGGIVTATDSNWMLSYTLNRQPHFKDQPKDQLVVWIYGLLSNKPGDFIKKSITECSGSEIAQEWLYHMGVPVDEIPDLAQN-SCNTIPCYMPYITSYFMPRAMGDRPLVVPNGSVNLAFMGNFSETERDTVFTTEYSVRTAMEAVYQLLDIDRGVPEVFASTYDIRTLLASTARLLDGKKLTDV-DAPFILKQIGKFGIHKTKETIIYDLLKESKLI------------------------

>Paenibacillus agri2

---------------------------------------------MGTYQRI-HPVMPEGIALRKAYLVGGGIGSLSAAAFLIRDGHMPGKNIHILEESPVYGGSMDGAGNAKDGYSARGGREI-EEHFECFMELYGFIPSLKNPDR-SVLDEFREFNLAEPVESHCRLVENAGQ-KADFSTLGLSTADSLQLGKLTMLTEERLGATTIEEFFD--PGFLETNFWYFWRSMFAFEHWHSVVEVKRYMERFMHLISGMNQLKGILHTEYNQYDSLILPLIKWLEGKGVNFDKGHQVVDLDLDLSSGEKTVTGIHVLVGG-ASKLIKVSRSDLVLVTNGSMTENSTLGDFNHPAIL-----NRSVSERGCWSLWEKMAAKSPD-FGRPEVFCGDIDKTKW-LSFTITLTDDDILFPHLLEWTGDKPG-----MGGVVTIKDSSWLMSWTAPTQPHFINQPDNVKVLWAYGLFPDAEGDYIKKKMSDCTGRELFEELLHHMGL-GNRIPEILSH-TTNVIPCMMPYITSQFMPRVMGDRPQVIPQGSTNLAFLGQFAEVPDDCVFTVEYSVRTAMMAVYGLLKLEKEVIPVHPSKYDVRVLLTAVGTCLGNKPLP-----------LDKTLGQILGGTVIGKLL------------------------------

>Paenibacillus helianthi2

--------------------------------------------------------MKKEYGNSQVYFVGGGIASLAGAAYLVRDCDFPGQNIHIIEEMKILGGSNDGSGSGEQGYVIRGGRMLNDETYENLWELLSTIPSIDHPGQ-SLREEITAFDDANPTHSNARLINREGE-VEDVLSMGFDMADRLAMGKLIITPEDKMGKARINDWFG--PHFFQTNFWYMWATTFAFQPWHSAVEFKRYMIRFMHEFPRIQTLEGVTRTPYNQYDSIILPLHKYLEPFGVDFTLKCTVTDLDFK-DGEGITVTKMHVLRQG-VPDVIEIGEEDRVIITNGSMTEGSSLGSM-TTAPG-----LNGK--GSSWKLWENIAAKKPG-LGNPSPFDDHVDGSKW-ESFTVTFQDSAF-FDLMEKFTRNRAG-----TGALVTFKDSSWFMSVVLAFQPHFRNQPEHVKVFWGYGLYPDKVGDFVKKRMCDCTGEEIMQELIGHLHF-EAHKEEIMAT--VSCIPCMMPYITSQFMPRLNSDRPQVVPAGSTNLAFVGQYCEIPDDVVFTEEYSVRAARIAVYTLMGVNRPVEPIKQHHYDVRTLFTSFVTSFR----------------------------------------------------------------

>Paenibacillus monticola2

---------------------------------------------MGTYQRI-NPLVQEGIASKKAYLVGGGIASLSAAAFLIRDGHMPGKNIHILEQSAIYGGSMDGAGNAKDGYSARGGREI-EEHFECFMELYGFIPSLTNPDR-TVLDEFRELNLAEPIESHCRLVENEGQ-KADFSSLGLSTAHALQLGKLTLATEEKLGAVTIEQFFD--PTFLETNFWYFWRSMFAFENWHSVVEVKRYMERFMHLISGMNQLKGILHTEYNQYDSLILPLLKWLEAQGVNFDKGHQVVDLDYDFANGETTVTGIQLLVNG-SPKTISTTRNDLVMVTNGSMTENSTRGDMDHPAIL-----NRSLTERGCWSLWEKLAAKSPD-FGHPEVFCGDIDKTKW-LSFTMTFTDDEIVFPYLLELTGDLPG-----MGGVVTIKDSNWMMSWTAPKQPHFINQPENVKVLWAYGLFPDTEGNFIKKKMADCTGSELLQELCYHIGL-KDRIPEILEH-TTNVIPCMMPYITSQFMPRVLGDRPEVVPQGSTNFAFLGQFAEVADDCVFTVEYSVRSAMMASYQLLNLEKEVIPVHPSKYDIRVLLTALRTCMGNKTLP-----------LDKTLGQLLSGTVIAKLLL-----------------------------

>Paenibacillus phocaensis2

---------------------------------------------MGTYQRI-HPRVPEGIAAKKAYLVGGGIGSLSAAAFLIRDGHMPGRNIHILEESAIYGGSMDGAGNAKDGYSARGGREI-EEHFECFMELFGFIPSLTDPDR-TVLDEFRELNLAEPIESHCRLVENAGQ-QADFSTLGLSTAHALQLGKLAMLTEEKLGRTTIEQFFD--ASFLETNFWYFWRSMFAFENWHSVVEVKRYMERFMHLISGMNRLKGILHTEYNQYDSLILPLVKWLEAQGVHFDKGHQVVDLELDMTGGEKVVTAIHLKVNG-AAKTIPVSRGDLVMVTNGSMTENSTKGDLNRPAVL-----NRSVEERGCWSLWEKLAAKSPD-FGRPEVFCGDIDKTKW-LSFTVTLTDDDIVFPHLLELTGDLPG-----MGGVVTIKDSNWLMSWTAPKQPHFLNQPDNVKVLWAYGLFPDAVGNYVGKKMADCTGRELLEELCYHMGL-KDRIPEILAH-TTNVIPCMMPYITSQFMPRVAGDRPQVVPEGSVNLAFLGQFAEVPDDCVFTVEYSVRTAMTAVYRLLKLEKEVIPVHPSRYDVRVLLTAVRTCLGNKPLP-----------LNRSLGELLSGTEIAKLLNG----------------------------

>Paenibacillus phytohabitans2

------------------------------MIPSTAALYYTRFRYSNQANLWRRQSVKKEYGNEQVYFVGGGIASLAGAAYLVRDCGFPGQNIHIIEEMKILGGSNDGSGNEEHGYVIRGGRMLNDETYENLWELLNTIPSIDQPGL-TLREEITQFDDANPTRSKARLVDSKGE-IQDVNSMGFDMADRLALGKLIITPEAKMGKARINDWFG--PHFFTTNFWYMWATTFAFQPWHSAVELKRYMIRFIHEFPRIQTLEGVTRTPYNQYDSIILPLHQYLEPFGVDFTLKCTVTDLEFK-EGDGITVTKMKVSRQG-VEDVIEINEGDRVIITNGSMTEGSSLGSM-TSAPR-----LNGK--GSSWKLWENIAAKKPG-LGNPSSFDDHVDESKW-ESFTVTFQDSKF-FDLMEDFTRNRAG-----TGALVTFKDSSWLMSVVLAFQPHFRNQPEHVKVFWGYGLYPDKVGDYVHKKMCDCTGEEIMKELIGHLHF-EAHKEEIMAT--ANCIPCMMPYITSQFMPRLNSDRPQVVPEGSSNLAFVGQYCEIPDDVVFTEEYSVRAARIAVYTLFGINRPVEPIKEYQHDVRTLLSSLVTSFR----------------------------------------------------------------

>Paenibacillus silagei2

---------------------------------------------MGTYQRI-HPQVQEGIASRKAYLVGGGIGSLSAAAFLIRDGHMPGRNIHILEQSSIYGGSMDGAGNAKDGYSARGGREI-EEHFECFMELFGFIPSLTNPDW-TVLDEFRELNLAEPIESHCRLVEKQGT-PADFSSLGLSTAHALQLGKLTLATEERLGTVTIEQFFD--PSFLETNFWYFWRSMFAFENWHSVVEVKRYMERFMHLISGMNQLKGILHTEYNQFDSLILPLMKWLEREGVHFDKGHEVTDLELSINGDEKVITAIQVQVNG-SPKTIPVTRADLVMVTNGSMTENSTLGDLDHPAVL-----NRSVTERGCWSLWEKLAAKSPD-FGRPEVFCGDIDKSKW-LSFTMTFTDDEIVFPYLLELTGDAPG-----MGGVVTIKDSSWMMSWTAPKQPHFINQPDNVKVLWAYGLFPDAEGDYIKKKMSDCTGRELLEELCYHMGL-VDRIPEILEH-TTNVIPCMMPYITAQFMPRALGDRPQVVPQGSVNLAFLGQFAEVPDDCVFTVEYSVRSAMMAVYELLKLEKEVIPVHPSKYDVRVLLTALRTCLGNKPLP-----------LDKTLGELLAGTVLSKLI------------------------------

>Paenibacillus timonensis2

---------------------------------------------MGTYQRI-HPRVPEGIAAKKAYLVGGGIGSLSAAAFLIRDGHMPGKNIHILEESAVYGGSMDGAGNAKDGYSARGGREI-EEHFECFMELFGFIPSLTNPDR-TVLDEFRELNLAEPIESHCRLVENAGQ-QADFSTLGLSTAHALQLGKLAMLTEEKLGRTTIEQFFD--LSFLETNFWYFWRSMFAFENWHSVVEVKRYMERFMHLISGMNRLKGILHTEYNQYDSLILPLVKWLEAQGVRFNKGHQVVDLELDVSGGEKIVTAIHLKVNG-AAKTIPVSRGDLVMVTNGSMTENSTKGDLNRPAVL-----NRSVDERGCWSLWEKLAVKSPD-FGRPEVFCGDIDKTKW-LSFTITLTDDEIVFPHLLELTGDLPG-----MGGVVTIKDSNWLMSWTAPKQPHFLNQPDNVKVLWAYGLFPDAVGNYVGKKMADCTGRELLEELCYHMGL-KDRIPEILAH-TTNVIP-------------------------------------------------------------------FPV-----------------------------------------------------------------------------------

>Paenibacillus turicensis2

-------------------------------------------------------MIHMKHTDRQVYFVGGGLASLAGAAYLVRDCKMEGKNIHILESMHLLGGSNDGIGTPEGGFVARGGRMLNEETYENFWELFYTIPSIHQEGM-SVSEEIIAFDKKHPTHSNARLVDKDGQ-VVDVMSMGFDKHDRIAMAKLIVTPEEKLDYVKISDWFS--PHFFTTNFWYMWQTTFAFQEWSSLFEFKRYMLRMMFEYSRIQTLEGVTRTPYNQYDSVILPLQTYLEKAGVDFSIRCTVTDLDFA-DGDGITVTAIHCEEEN-GHKIIKLQPDDLCIVTNGCMTDAATLGNYKTPAPY-----SPEN--PISGMLWTKIANKKQG-LGNPQPFFGNPEETNW-ESFTVTLNGNKL-LKKIEKFSRNVPG-----SGALMTFKDSSWRLSIVVAAQPHFINQPEDQTIFWGYGLYTDRIGDYVKKPMRDCTGEEIFTELMHHLHF-DDELEEIKKD-IVNVIPCMLPYTDAQFQPRAMSDRPKVVPEGSTNLALISQFVEIPEDMVFTEEYSIRAARIAVYTLMGVNRPVCPVTPHASNVNTLLKALVTSYR----------------------------------------------------------------

>Paenibacillus brevis2

-----------------------------------------MYYSNGNYEAFARPEKPKNVERKSAYLVGSGLASLAAACFLVRDGQMKGEHIHILEEMSLPGGACDGIKDAQKGFIIRGGREM-ENHFECLWDLFRSIPSIETKDL-SVLDEFYRLNKHDPNYSLMRASVNRGEDAHTDGKFTLSEKASMQIVKLFMTRDEDLYDVTIDDVFD--EEFYASNFWLYWQTMFAFEKWHSALEMKLYLQRFIHHIGGLPDFSALKFTKYNQYESLILPMVKFLEDHGVHFQYDTRVTNVIFDIRDGKKTAKQLICIHNG-KEETIDLIEDDLVFVTNGSCTENSTLGDDDHAPVM-----NTQPGEGGCWQLWKNIAAQDPS-FGRPDKFCTNIPATNW-ESATVTTLDHRI-PRYIEKMSKRDPFSGKVVTGGIITVKDSSWLMSYTINRQPHFKEQPKDQVVVWVYGLYTDVPGDFVKKPMKECTGREIVEEWLYHMGVPEAEIRELAAT-GAHCIPCMMPYITAFFMPRTEGDRPKVVPEGSVNFAFIGQFADTVRDTVFTTEYSVRTAMEAVYTLLDVDRGVPEVFASCYDVRVLLDSTAKMLDGKKLTDL-KLPFIAKLVEKQLLKKASGTIIGELLERYKLI------------------------

>Elizabethkingia meningoseptica

MNTITSKFDKVLNASPDYGHVNHEPDSSTEQQCNTPQKSMPFSDQIGNYQRN-KGIPTKSYADSKIYIVGSGIAGMSAAYYFIRDGHVPAKNIVFLEQLHIDGGSLDGAGNAKDGYIIRGGREM-DMTYENLWDMFQDIPALEMPAPYSVLDEYRLINDNDSNYSKARLIHNKGI-IKDFSKFGLNKKDQLAIIRLLLKNKEELDDLSIQDYFS--ESFLNSNFWTFWRTMFAFENWHSLLELKLYMHRFLHAIDGLNDLSSLVFPKYNQYDTFVKPLRNFLKEKGVTIELNTLVKDLDIHVNTEGKVVEGIITEQDG-KEVKIPVRENDYVIVTTGSMTEDTFYGDNKKAPVIGIDNSTSGQ--SSGWKLWKNLAAKSEV-FGKPEKFCSNIEKSAW-ESATLTCKPSAL-IDKLKEYSVNDPYSGKTVTGGIITITDSNWLMSFTCNRQPHFPEQPDDILVLWVYALFMDKEGNYIKKTMPECTGDEILAELCHHLGI-TDQLENVQEN--TIVRTAFMPYITSMFMPRAKGDRPRVVPEGCKNLGLVGQFVETNNDVVFTMESSVRTARIAVYELLNLNKQVPDINPLQYDIRHLLKAAKTLNDDQPFI-----------GEGLLRKILKGTYFEHVLPGTAKEEEEHESFVMEQINRFKDWMKGIRG
