# Supplementary material for: Independent evolution of oleate hydratase clades in Bacillales reflects molecular convergence
Source: Front Mol Biosci. 2024 Dec 12;11:1485485. doi: 10.3389/fmolb.2024.1485485 (PMC11669549; doi:10.3389/fmolb.2024.1485485)
Supplement: Supplementary file 6 [file Image1.pdf]

# Supplemental Figures

## Independent evolution of oleate hydratase clades in Bacillales reflects molecular convergence

Robert J. Neff, Priscila C. Lages, Shannon K. Donworth, James D. Brien, Christopher D. Radka

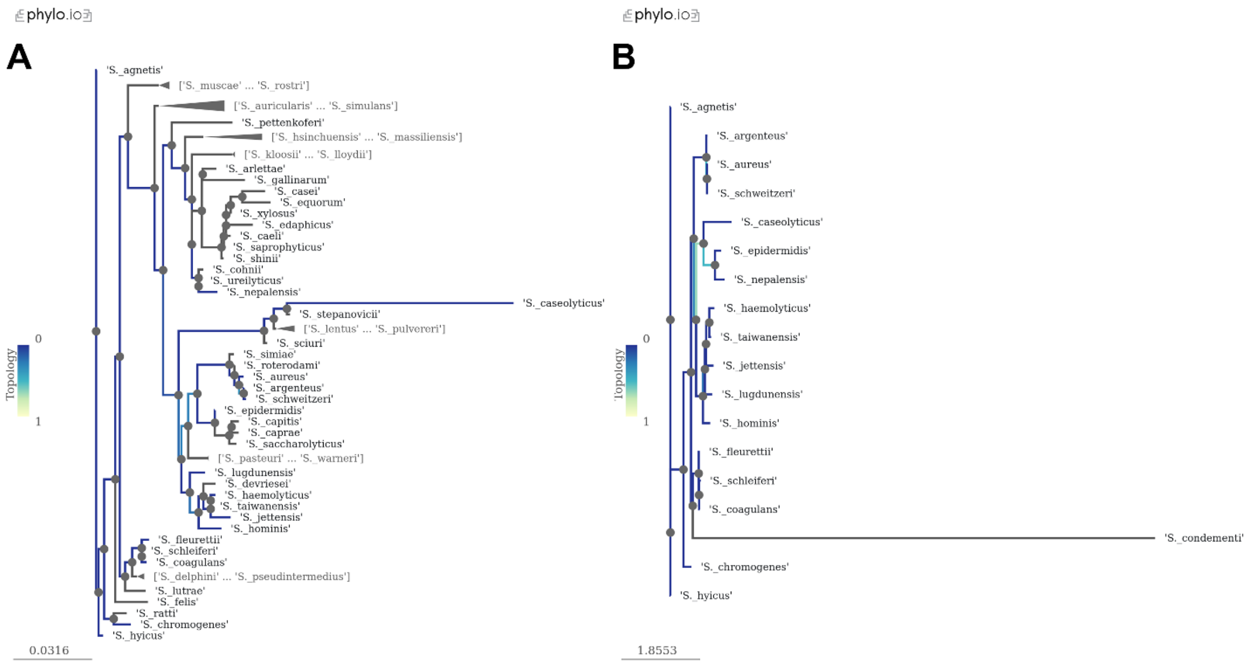


**Supplemental Figure 1. Comparison of *Staphylococcal* phylogenetic trees using phylo.io.** Phylogenetic tree based on *Staphylococcal* 16S rRNA sequences (*A*) compared phylogenetic tree based on *Staphylococcal ohyA* gene (*B*). Topological similarity between the two trees is quantified on a scale from 0 to 1, where 0 indicates maximum similarity and 1 indicates minimum similarity. Tree branches are color-coded with a gradient to represent the similarity scores for each branch.

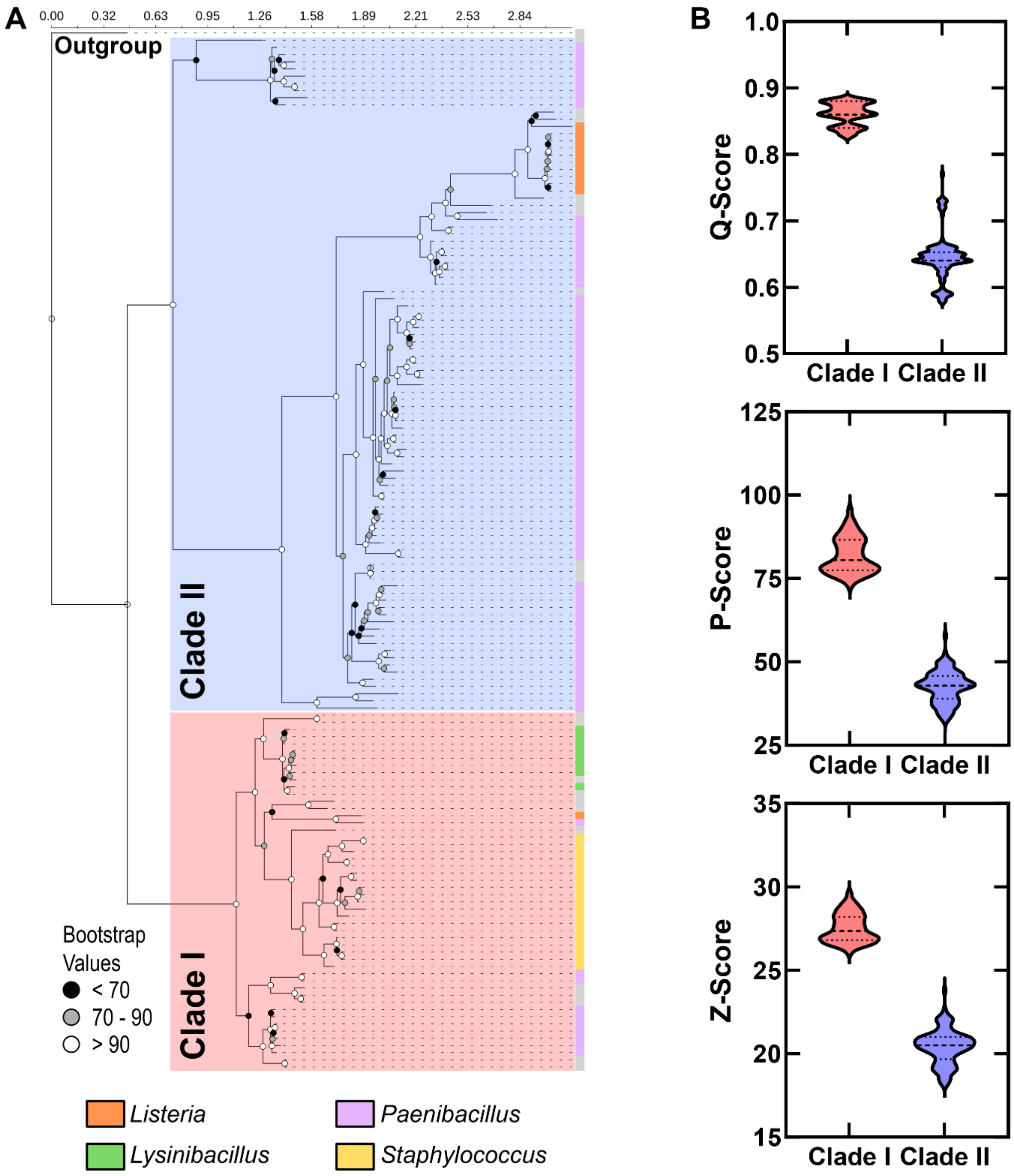


**Supplemental Figure 2. The membrane binding sequence does not determine OhyA clade classification.** *A*, Phylogram with outgroup rooting derived from 145 OhyA amino acid sequences, excluding the amino acids that comprise the membrane binding domain in clade I OhyAs. The total length of the multiple sequence alignment is 605 residues. The phylogram reveals two clades: clade I (red shading) and clade II (blue shading), consistent with those shown in Fig. 3. Bootstrap values supporting the nodes of the consensus tree are represented by spheres. The coloration on the crust of the tree indicates the distribution of species belonging to the four major *ohyA* genera, as labeled next to the tree. *B*, Statistical evaluation of alignment quality to Clade I *Staphylococcus aureus* OhyA using PDBeFOLD. AlphaFold models for all

Bacillales OhyA were employed for alignment. The distinct separation between clade I and Clade II models suggests significant structural differences between the two clades. Dotted lines indicate the median and quartiles.
